# First-Breath-Induced Type 2 Pathways Shape the Lung Immune Environment

**DOI:** 10.1016/j.celrep.2017.01.071

**Published:** 2017-02-21

**Authors:** Simona Saluzzo, Anna-Dorothea Gorki, Batika M.J. Rana, Rui Martins, Seth Scanlon, Philipp Starkl, Karin Lakovits, Anastasiya Hladik, Ana Korosec, Omar Sharif, Joanna M. Warszawska, Helen Jolin, Ildiko Mesteri, Andrew N.J. McKenzie, Sylvia Knapp

**Affiliations:** 1 CeMM Research Center for Molecular Medicine of the Austrian Academy of Sciences, Vienna 1090, Austria; 2 Department of Medicine I, Laboratory of Infection Biology, Medical University of Vienna, Vienna 1090, Austria; 3 MRC Laboratory of Molecular Biology, Francis Crick Avenue, Cambridge CB2 0QH, UK; 4 Institute of Pathology Überlingen, Überlingen 88662, Germany

**Keywords:** newborn, lung, immune homeostasis, alarmin, first breath, alveolar macrophage, pneumoniae, S. pneumoniae

## Abstract

From birth onward, the lungs are exposed to the external environment and therefore harbor a complex immunological milieu to protect this organ from damage and infection. We investigated the homeostatic role of the epithelium-derived alarmin interleukin-33 (IL-33) in newborn mice and discovered the immediate upregulation of IL-33 from the first day of life, closely followed by a wave of IL-13-producing type 2 innate lymphoid cells (ILC2s), which coincided with the appearance of alveolar macrophages (AMs) and their early polarization to an IL-13-dependent anti-inflammatory M2 phenotype. ILC2s contributed to lung quiescence in homeostasis by polarizing tissue resident AMs and induced an M2 phenotype in transplanted macrophage progenitors. ILC2s continued to maintain the M2 AM phenotype during adult life at the cost of a delayed response to *Streptococcus pneumoniae* infection in mice. These data highlight the homeostatic role of ILC2s in setting the activation threshold in the lung and underline their implications in anti-bacterial defenses.

## Highlights

• The first breath triggers IL-33 induction by AEC2 in lungs of newborn mice
• IL-33 promotes the perinatal expansion and activation of ST2-expressing ILC2s
• ILC2-derived IL-13 polarizes newborn’s AMs into an M2 phenotype
• This homeostatic type 2 pathway delays antibacterial effector responses

## Graphical Abstract

**Figure:**
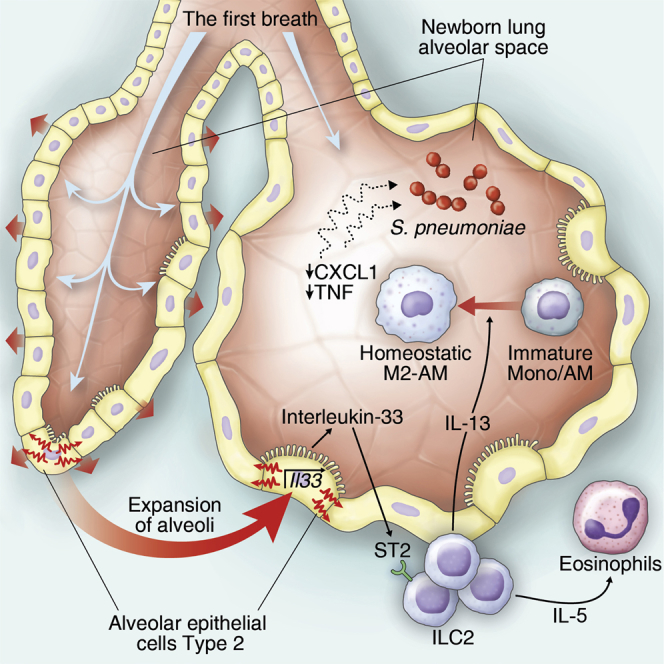

## Teaser

The postnatal lung immune environment is largely unexplored. Saluzzo et al. find that birth is associated with the induction of an IL-33/ILC2/IL-13-driven immune response in mice. This type 2 response is preserved throughout life and maintains alveolar macrophages in an M2 phenotype at the expense of a delayed response to bacterial infections.

## Introduction

The integrity of the alveolar-capillary barrier is essential to ensure sufficient blood oxygen levels, and the mechanisms driving its maintenance, renewal, and protection are flourishing fields of research (Beers and Morrisey, 2011, Chiu and Openshaw, 2015, Hogan et al., 2014, Hussell and Bell, 2014, Kopf et al., 2015, Peng et al., 2015). Lung development begins at embryonic day 9 (E9) in mice and proceeds through stages of branching morphogenesis, giving rise to pre-alveolar spaces at the saccular stage and the differentiation of type 1 and type 2 airway epithelial cells (AEC1s and AEC2s) by E18.5 (Mund et al., 2008, Woik and Kroll, 2015). At birth, alveolar sacs are suddenly exposed to the external environment and subjected to the mechanical forces of spontaneous ventilation (Orr et al., 2006, Wirtz and Dobbs, 2000). It is after the previously sterile lung tissue has been exposed to the outside environment, around postnatal day 4 (P4), when the process of alveologenesis continues with the formation of primary septa (Hogan et al., 2014). These postnatal adaptations are paralleled by the development of the early innate immune environment. Alveolar macrophages (AMs) differentiate on P3 from CD11b^hi^F4/80^int^Ly6C^hi^ fetal monocyte progenitors into long-lived, self-renewing cells (Guilliams et al., 2013, Murphy et al., 2008). Since tissue-derived signals were found to govern the gene expression signature of macrophages (Lavin et al., 2014, Okabe and Medzhitov, 2014), the lung cytokine milieu in newborns likely determines the phenotype of AMs during this delicate developmental period. However, the postnatal immunological environment in lungs is largely unexplored, as are the innate immune signals that influence the function of AMs early in development and during homeostasis.

Under different pathological conditions, AMs have the ability to assume either an interferon-γ (IFN-γ) and Toll-like receptor (TLR) ligand-induced inflammatory phenotype (M1) or an interleukin-4 (IL-4)-, IL-13-, or IL-10-induced wound healing and tissue remodeling phenotype (M2) (Gordon and Martinez, 2010). M1 macrophages are potent producers of inflammatory cytokines such as tumor necrosis factor (TNF) and CXCL1 (Guery et al., 2011, Mantovani et al., 2004). CXCL1 is a chemokine that critically determines the early recruitment of neutrophils (De Filippo et al., 2013), thereby exerting a protective role in bacterial lung infections (Schliehe et al., 2015, Warszawska et al., 2013). M2 macrophages, phenotypically defined by the expression of *Retnla* (referred to here as *Fizz1*), *Mrc1*, *Chil3* (referred to here as *Ym1*), and *Arg1* are less efficient in triggering inflammatory responses to bacterial pathogens than M1 macrophages (Warszawska et al., 2013).

IL-33 is an alarmin belonging to the IL-1 family of cytokines best known for its capacity to drive type 2 immune responses (Liew et al., 2010, Schmitz et al., 2005) and is increasingly recognized as an important mediator of homeostasis and tissue tolerance (Molofsky et al., 2015). Upon mechanical strain or cell necrosis (Kakkar et al., 2012, Lamkanfi and Dixit, 2009, Sanada et al., 2007), IL-33 is released from cells, activating the ST2 receptor expressed on several lung cell types, including regulatory T cells (T_reg_), dendritic cells (DCs), mast cells, group 2 innate lymphoid cells (ILC2s), natural killer (NK) cells, and AMs (Lu et al., 2015). At steady state, lung resident ILC2s are the most abundant ST2-expressing cells and are found in close proximity to bronchovascular structures (Halim et al., 2014, Nussbaum et al., 2013), where they can be rapidly activated by IL-33 to secrete IL-13, IL-5, IL-6, IL-9, granulocyte-macrophage colony-stimulating factor (GM-CSF), and amphiregulin (Roediger and Weninger, 2015). ILC2s are involved in host protection against parasitic helminths and promotion of airway hyperreactivity in asthma or upon influenza infection and are important for adipose tissue homeostasis (Barlow et al., 2012, Brestoff et al., 2015, Chang et al., 2011, Molofsky et al., 2013, Monticelli et al., 2011, Neill et al., 2010).

Human lungs are highly susceptible to bacterial infections. Pneumonia caused by *Streptococcus pneumoniae* is the primary cause of death by an infectious disease in Western countries (van der Poll and Opal, 2009). Notably, risk factors for developing community-acquired pneumonia are asthma and influenza (Chien et al., 2009, Talbot et al., 2005), which are both characterized by IL-13-induced airway hyperreactivity (Kim et al., 2012, Lambrecht and Hammad, 2015) and the presence of M2 polarized AMs (Chen et al., 2012). Here, we investigated the physiological role of the IL-33/ILC2/IL-13 axis in shaping the pulmonary immune environment from birth to adult life and the consequences of these pathways on the innate defense against *S. pneumoniae*.

## Results

### Postnatal Lung Inflation Is Associated with the Upregulation of IL-33 by AEC2

**Figure 1:**
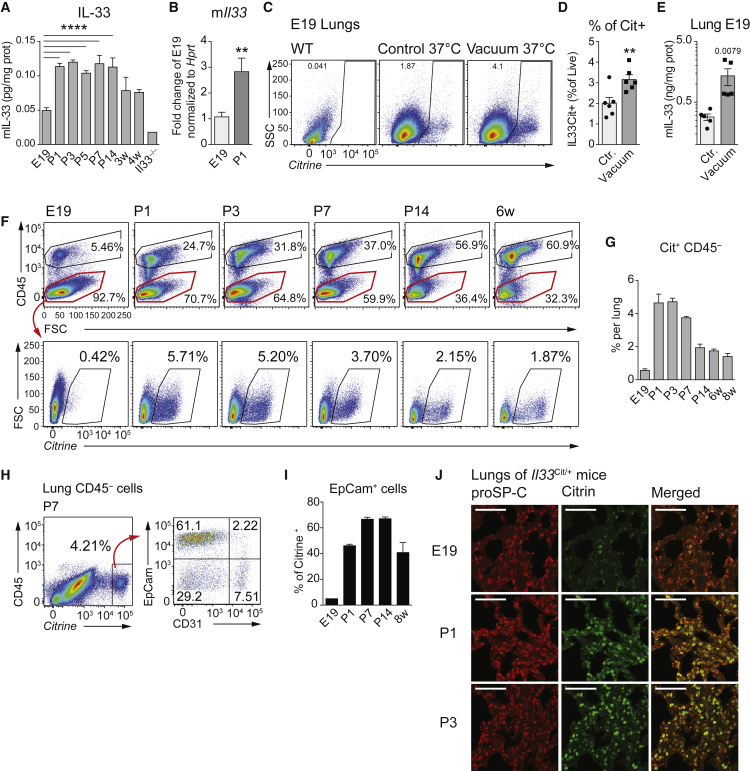
Type 2 Alveolar Epithelial Cells Induce IL-33 at Birth (A) Whole-lung IL-33 quantification by ELISA at E19; postnatal days 1, 3, 5, 7, and 14 (P1–P14); and 3 and 4 weeks (3–4w) after birth. (B) qRT-PCR of pulmonary *Il33* expression in WT mice at E19 and P1. (C) FACS analysis of viable citrine^*+*^ cells from *Il33*^*Cit/+*^ mice at E19 exposed to vacuum or atmospheric pressure (control) for 6 hr. (D) Quantification of (C). (E) Whole-lung IL-33 quantification by ELISA of WT lungs at E19 exposed to vacuum or atmospheric pressure (control) for 6 hr. (F) FACS analysis of lung CD45 and citrine expression in *Il33*^*Cit/+*^ reporter mice at the indicated time points (gates are set using WT as controls). (G) Percentage of Cit^*+*^CD45^−^ cells among lung cells, gated as in (F). (H) Flow cytometry of viable CD45^−^ lung cells from *Il33*^*Cit/+*^ mice at P7, stained for EpCam and CD31. (I) Quantification of the *Cit*^*+*^ proportion of EpCAM^+^ cells between E19 and 8 weeks of age. (J) Micrographs of lung sections at E19, P1, and P3 from *Il33*^*Cit/+*^ reporter mice. Red, surfactant protein C (SP-C); green, IL-33-driven citrine. Scale bars represent 75 μm. Data are representative of two independent experiments with three to five mice per time point, and graph bars represent mean ± SEM. ^∗∗^p < 0.01 and ^∗∗∗∗^p < 0.0001.

With the first breath, a number of profound changes occur in the newborn’s lung. We hypothesized that the sudden inflation of the previously liquid-filled lungs may cause considerable mechanical stress and potential tissue injury, which could result in IL-33 induction (Kakkar et al., 2012). We discovered a substantial increment in pulmonary IL-33 on P1 compared to E19 at both the protein (Figure 1A) and mRNA levels (Figure 1B). To investigate if an abrupt exposure to negative pressure, occurring upon spontaneous breathing in the alveolar space, might cause the induction of *Il33*, we placed the lungs of E19 *Il33*^*Cit/+*^ reporter (Hardman et al., 2013) and WT mice in a vacuum chamber (Figure S1A) and discovered a significant induction of Citrine^*+*^ viable cells (Figures 1C, 1D, S1B, and S1C) and IL-33 protein (Figure 1E) in lungs 6 hr post-exposure to negative pressure as compared to ambient atmospheric pressure.

To study the cellular origin of pulmonary IL-33 over time, we analyzed lungs of *Il33*^*Cit/+*^ reporter mice by flow cytometry. We observed a strong upregulation of *Il33* among the CD45^−^ cell fraction starting on P1 (Figures 1F, 1G, S1D, and S1E). Approximately 60% of CD45^−^ citrine^*+*^ cells were further classified as EpCam^+^CD31^−^ cells (Figures 1H and 1I). Immunohistochemistry revealed that AEC2 (surfactant protein C^+^) was the most abundant cell population upregulating *Il33* in the first few days after birth (Figure 1J). Postnatally infiltrating CD45^+^ cells (Figure 1F, top, and Figure S1F) did not show substantial *Il33* expression (Figure S1G), except for few citrine^+^ cells in the fetal macrophage fraction (CD45^+^F4/80^+^CD11b^+^CD11c^−^ SiglecF^−^) (Figures S1H and S1I). In summary, we determined that postnatal lung inflation or exposure to abrupt changes in pressure was associated with the immediate induction of IL-33.

### IL-33 Shapes the Neonatal Lung Environment

**Figure 2:**
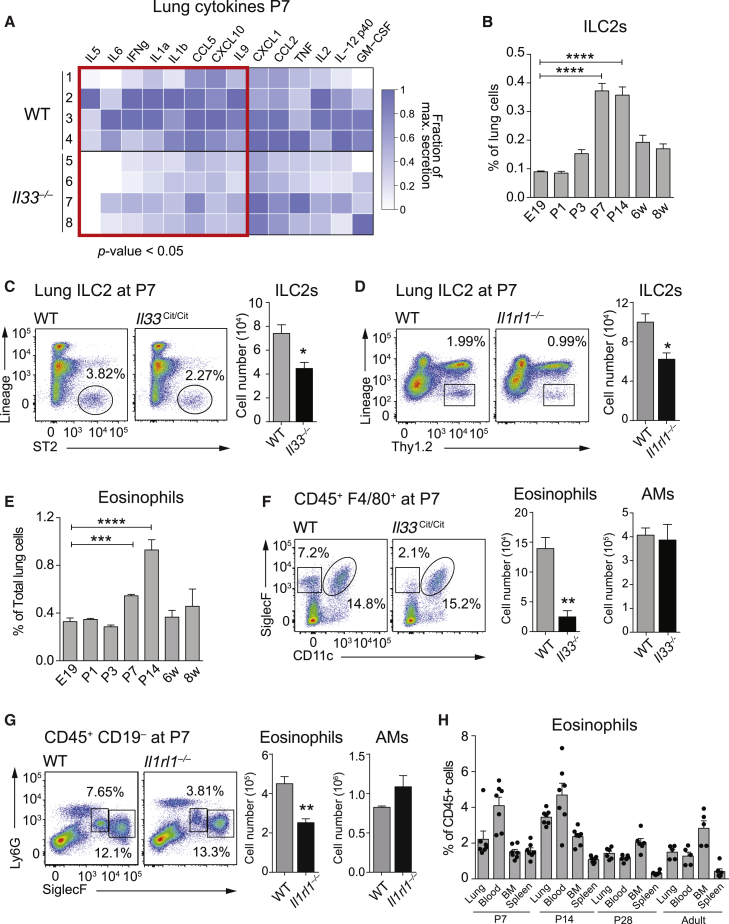
IL-33 Drives a Type 2 Immune Environment in Lungs of Newborns (A) Heatmap representation of cytokine levels in whole lung homogenates comparing WT and *Il33*^*Cit/Cit*^ mice at P7. Original values (see Figure S2A) were rescaled between zero and the maximum value detected for each cytokine and are presented as the fraction of maximum secretion. (B) Percentage of lung ILC2s (Lin^−^ ST2^+^ Thy1.2^+^ CD25^+^ ICOS^+^) analyzed by FACS at the indicated time points. (C) FACS analysis of lung ILC2s (Lin^−^ ST2^+^) in WT and *Il33*^*Cit/Cit*^ mice at P7, further gated for CD25^+^ and ICOS^+^ and quantified (right). (D) FACS analysis of lung ILC2s (Lin^−^ Thy1.2^+^) in WT and *Il1rl1*^−/−^ mice at P7, further gated for CD25^+^ and ICOS^+^ and quantified (right). (E) Percentage of lung eosinophils (F4/80^+^ CD11b^+^SiglecF^+^CD11c^−^) analyzed by FACS at the indicated time points. (F and G) FACS analysis of lung eosinophils (CD11b^+^SiglecF^+^CD11c^−^) and AMs (CD11b^−^SiglecF^+^CD11c^+^) at P7 in WT and *Il33*^*Cit/Cit*^ mice (F) and WT and *Il1rl1*^−/−^ mice (G). (H) Lung, blood, bone marrow, and spleen cells were analyzed by FACS for eosinophils (CD11b^+^SiglecF^+^F480^+^CD11c^−^) in P7, P14, P28, and adult (6–8 weeks) *Il5*^*Cer/+*^ mice. Data are representative of one (A and H) or two (B–G) independent experiments with four mice per group. Graph bars represent mean ± SEM. ^∗^p < 0.05, ^∗∗^p < 0.01, ^∗∗∗^p < 0.001, and ^∗∗∗∗^p < 0.0001. For flow cytometry, all cells were pre-gated on viable, single, CD45^+^. E, embryonic; p, postnatal; w, week.

To understand IL-33-dependent effects on the immune environment in neonatal lungs, we first analyzed a panel of pulmonary cytokines and chemokines at P7 in wild-type (WT) and IL-33-deficient (*Il33*^*Cit/Cit*^) mice. *Il33*^*Cit/Cit*^ mice showed reduced expression of the type 2 cytokines IL-5 and IL-9 and of inflammatory mediators like IL-6, IFN-γ, IL-1α, IL-1β, CCL5, and CXCL10 (Figures 2A and S2A). Since ST2^+^ ILC2s are major producers of IL-5 and Il-9 and considered the primary targets of IL-33 in the lung (Halim et al., 2014, Kearley et al., 2015), we analyzed newborn lungs for the presence of ILC2s (Lin^−^ CD127^+^ST2^+^ICOS^+^). We detected few ILC2s at E19 but markedly increased numbers by P7 that stabilized by week 6 (Figures 2B and S2B). We found IL-33 to be contributory in populating lungs with ILC2s, as illustrated by reduced ILC2 numbers in *Il33*^*Cit/Cit*^ (Figure 2C) and ST2-deficient (*Il1rl1*^−/−^) mice at P7 (Figure 2D). In accordance with the ability of ILC2s to regulate eosinophil homeostasis via IL-5 secretion (Nussbaum et al., 2013), eosinophils populated the lungs a few days after ILC2s (Figures 2E and S2C), with clear reductions in the absence of IL-33 (Figure 2F) or ST2 (Figure 2G). IL-5+ ILC2s expanded locally in the lungs (Figures S2E–S2G), whereas eosinophil numbers increased systemically (Figure 2H). The numbers of AMs, polymorphonuclear (PMNs) cells, B cells, and T cells were not changed in *Il33*^*Cit/Cit*^ mice (Figures 2F, 2G, and S2D). Importantly, the postnatal alveolarization process (Hogan et al., 2014) was not affected by the absence of IL-33 (Figure S2H).

Collectively, these data indicate a critical role for IL-33 in shaping the immune cell infiltrate in the neonatal lung by promoting the appearance of ILC2s and eosinophils. This early period, in which the lung immunological environment is being established, may have subsequent effects on adult lung homeostasis and host defense.

### AM Development in Neonatal Lungs Coincides with ILC2 Activation

**Figure 3:**
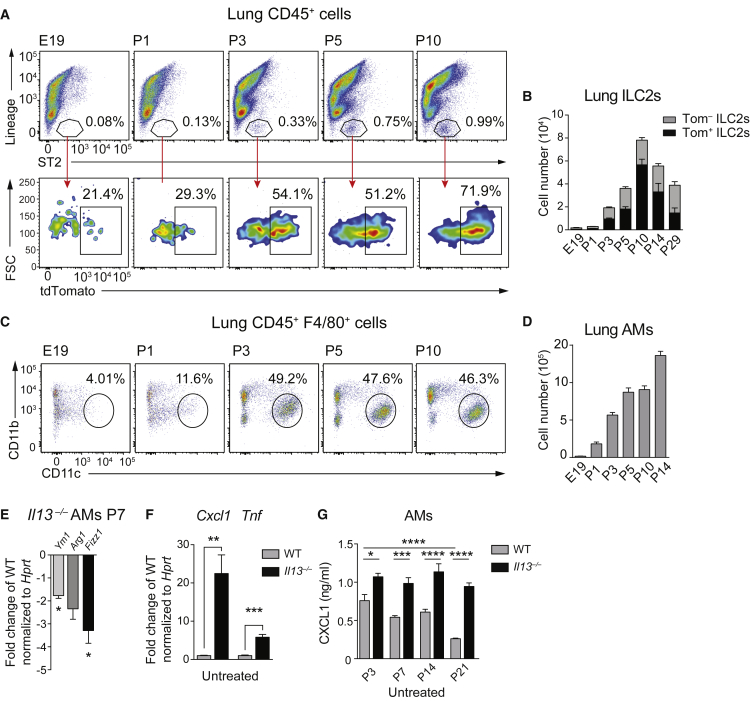
Lung ILC2s Expansion and Activation Coincides with AM Differentiation and M2 Polarization (A) Representative FACS profiles of expanding lung ILC2s (Lin^−^ ST2^+^) (top) and proportion of *Tom*^+^ cells (bottom) in *Il13*^*Tom/+*^ mice between E19 and P10. (B) Quantification of absolute numbers of lung Lin^−^ ST2^+^ Thy1.2^+^ Tom^+/−^ cells at the indicated time points. (C) FACS plots illustrating percentages of AMs (F4/80^+^CD11b^−^CD11c^+^) at the indicated time points. (D) Absolute numbers of AMs gated as in (C) between E19 and P14. (E) AMs (F4/80^+^CD11b^−^CD11c^+^SiglecF^+^) were sorted on P7 from WT and *Il13*^*Tom/Tom*^ mice and M2 markers were assessed by RT-PCR. (F) AMs from WT and *Il13*^*Tom/Tom*^ (IL-13 deficient) mice on P7 were isolated as in (E) and cultured for 6 hr, and *Cxcl1* and *Tnf* gene induction was assessed by RT-PCR. Values were normalized to *Hprt* and are expressed as fold change versus WT. (G) AMs from WT and *Il13*^*Tom/Tom*^ (IL-13 deficient) mice on P3, P7, P14, and P21 were isolated as in (E) and cultured for 6 hr, and spontaneous CXCL1 secretion was quantified by ELISA. Data are representative of three (A–D) or two (E–G) independent experiments with three or four mice per group. Values were normalized to *Hprt* and are expressed as fold change versus the indicated control. Bars represent mean ± SEM; ^∗^p < 0.05, ^∗∗^p < 0.01, ^∗∗∗^p < 0.001, and ^∗∗∗∗^p < 0.0001.

We next examined the activation state of postnatally expanded ILC2s in lungs using *Il13* tdTomato (*Il13*^*Tom/+*^) reporter mice (Barlow et al., 2012). IL-13-expressing ILC2s began to expand at P3, peaked at 70% on P10, and started to decline by P14 (Figures 3A, 3B, and S3A–S3C). Perinatal IL-13 expression was restricted to Lin^−^ cells (Figure S3D) and depended on the presence of IL-33 (Figure S3E). The expansion of activated ILC2s coincided with the emergence of AMs (Figures 3C, 3D, and S3F).

Considering the critical role of IL-13 in driving the alternative activation of macrophages, we tested to what degree this postnatal wave of ILC2-derived IL-13 might contribute to the immediate polarization of newly differentiated AMs (Gordon and Martinez, 2010). We discovered reduced expression levels of the M2 markers *Ym1*, *Arg1*, and *Fizz1* in *Il13*^−/−^ and *Il1rl1*^−/−^ AMs (Figures 3E and S3G). Further, we found elevated spontaneous expression levels of *Cxcl1* and *Tnf* in *Il13*^−/−^ as compared to WT AMs on P7 (Figures 3E and 3F). Remarkably, the amount of CXCL1 released by WT AMs declined with age, whereas AMs from *Il13*^−/−^ mice continued to produce high levels of CXCL1 until P21 (Figure 3G). These data demonstrate that postnatal AMs exhibit an M1 phenotype and that IL-13 promotes the deactivation and M2 polarization of AMs over time.

### IL-13 Maintains Adult Resident AMs in an M2 State

**Figure 4:**
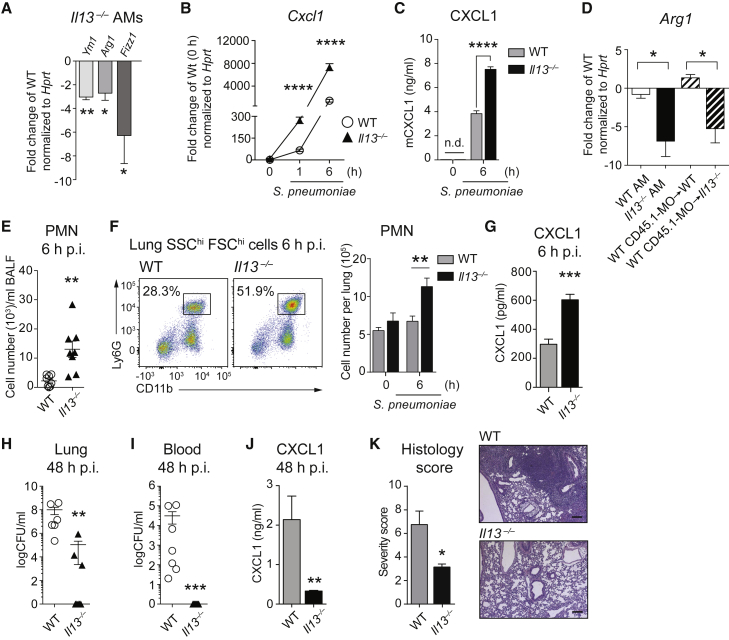
AMs from IL-13-Deficient Mice Present a Pro-inflammatory Phenotype and Improved Defenses against *S. pneumoniae* (A) AMs from adult WT and *Il13*^−/−^ mice were isolated by bronchoalveolar lavage and analyzed for expression of M2 polarization markers by RT-PCR. (B and C) AMs isolated as in (A) were in vitro stimulated with *S. pneumoniae* (MOI 100). The induction of *Cxcl1* was quantified by RT-PCR (B), and supernatant protein levels were determined by ELISA (C). (D) CD45.1 WT monocytes were intra-tracheally transferred to WT and *Il13*^−/−^ CD45.2 recipients, and bronchoalveolar cells were harvested by lavage 2 weeks later. FACS-sorted recipient AMs and monocyte-derived AMs were analyzed for expression of M2 polarization markers by RT-PCR. (E–G) WT and *Il13*^−/−^ mice were i.n. infected with *S. pneumoniae* and sacrificed after 6 hr. PMN numbers in BALF were assessed on cytospins (E) and in lungs by FACS analysis (CD45^+^SSC^hi^FSC^hi^CD11b^+^ Ly6G^+^) (F). Lung CXCL1 was quantified by ELISA (G). (H–K) WT and *Il13*^−/−^ mice were i.n. infected with *S. pneumoniae* and sacrificed after 48 hr. CFU counts in lung homogenates (H) and blood (I). Lung CXCL1 was quantified by ELISA (J). H&E-stained lung sections were scored by a pathologist (see Experimental Procedures) (K, left). Representative H&E lung sections (K, right). Scale bars represent 180 μm. Data are representative of at least three independent experiments with four (A–C) and seven or eight (E–K) mice per group. Data in (D) are from a single experiment with six mice per group. PCR values were normalized to *Hprt* and expressed as fold change versus indicated control. Mean ± SEM are depicted; ^∗^p < 0.05, ^∗∗^p < 0.01, ^∗∗∗^p < 0.001, and ^∗∗∗∗^p < 0.0001. BAL, bronchoalveolar lavage; CFU, colony-forming units; p.i., post-infection; PMN, polymorphonuclear cells.

AMs are long-lived cells with local self-renewal capacity, which are, like other macrophages, strongly influenced by the environment they inhabit (Guilliams et al., 2013, Lavin et al., 2014, Murphy et al., 2008). We reasoned that the need for an unremittingly quiescent lung environment throughout life would favor an M2 AM phenotype and discovered that pulmonary IL-13 continued to affect the M2 polarization of AMs in adult mice (Figure 4A). Adult *Il13*^−/−^ AMs stimulated with the respiratory pathogen *S. pneumoniae* or the TLR2 ligand lipoteichoic acid (LTA) consistently induced higher levels of CXCL1 than WT AMs (Figures 4B, 4C, and S4A). WT monocytes adoptively transferred to the lungs of WT or *Il13*^−/−^ mice differentiated toward a SiglecF^+^ AM phenotype within 2 weeks (Figure S4B) and upregulated M2 markers in WT, but not *Il13*^−/−^, recipient animals (Figures 4D and S4C). These results confirm that a tissue-derived source of IL-13 is required to polarize and maintain AMs in an M2 state in adult mice. Of note, IL-33 itself was not sufficient to shape the polarization and activity of AMs from adult mice, as *Il1rl*^−/−^ AMs did not differ from WT AMs in their expression of M2 markers or response to *S. pneumoniae* (Figures S4D–S4F). In summary, endogenous IL-13 contributes to the M2 phenotype of resident and monocyte-derived AMs in healthy adult mice and is required to suppress potentially excessive inflammation.

### Pulmonary IL-13 Is Detrimental upon Pneumococcal Infection

We hypothesized that the IL-13-driven M2 polarization of AMs might impact on innate defenses against *S. pneumoniae*. Upon infection of *Il13*^−/−^ and WT mice with *S. pneumoniae*, we observed a more pronounced early (6 hr) influx of neutrophils in bronchoalveolar lavage fluid (BALF) and lung (Figures 4E and 4F) and enhanced amounts of lung CXCL1 (Figure 4G) in *Il13*^−/−^ mice. This augmented early inflammatory response in the absence of *Il13* translated into an improved bacterial clearance from lungs 48 hr post-infection and completely prevented the systemic spread of bacteria (Figures 4H and 4I). In accordance with the reduced bacterial burden, we found decreased CXCL1 levels (Figure 4J), lower numbers of infiltrating monocytes (Figure S5A), and less pronounced lung infiltrates at 48 hr post-infection in *Il13*^−/−^ as compared to WT animals (Figure 4K). To assess the broader relevance of these findings, we investigated the contribution of pulmonary IL-13 to host defense against *Staphylococcus aureus* as well as upon induction of lipopolysaccharide (LPS)-induced acute lung injury. Similar to our observations in pneumococcal pneumonia, we discovered an augmented early inflammatory response to LPS (Figures S5B and S5C) and an improved clearance of *S. aureus* associated with a reduced disease-associated temperature drop in *Il13*^−/−^ animals (Figures S5D and S5E). Together, these results support the notion that pulmonary IL-13 shapes the immune environment in the lung, which upon infection delays the induction of innate defenses against pathogens.

We could not detect any baseline differences in immune cells involved in the defense against bacteria (NK, T, B, or PMN cells and monocytes; Figure S5F; data not shown) or in ILC2 levels (Figure S5G), except for an increased number of eosinophils in *Il13*^−/−^ mice (Figure S5H). To exclude the possibility that eosinophils contributed to the phenotype, we repeated the infection studies in *Il5*^−/−^ mice, which have severely reduced pulmonary eosinophilia (Figure S5I), and could not identify any differences in bacterial counts (Figure S5J) or the inflammatory response elicited by AMs in vitro (Figure S5K).

**Figure 5:**
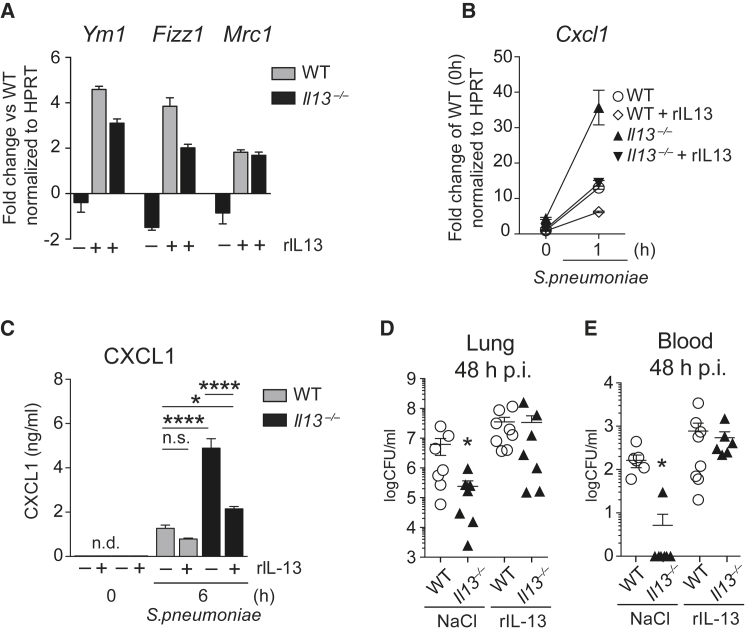
Intranasal rmIL-13 Treatment Reversed the Inflammatory Phenotype of AMs in *Il13*^−/−^ Mice and the Responses to *S. pneumoniae* (A–C) WT and *Il13*^−/−^ mice were treated daily with rmIL-13 (6 ng in 50 μL NaCl) i.n., and AMs were isolated by bronchoalveolar lavage on day 3. M2 markers were assessed by RT-PCR (A). Cultured AMs were stimulated with *S. pneumoniae* (MOI 100), and fold induction of *Cxcl1* was measured by RT-PCR (B). AMs were treated as in (B), and CXCL1 protein was quantified by ELISA (C). (D and E) WT and *Il13*^−/−^ mice were treated with mrIL-13 as in (A)–(C), infected i.n. with *S. pneumoniae* on day 3, and sacrificed after 48 hr. CFU counts in lung homogenates (D) and blood (E) are shown. Data in (A)–(C) are representative of two independent experiments with four mice per group. Data in (D) and (E) are from a single experiment with eight mice per group. Mean ± SEM are depicted; ^∗^p < 0.05 and ^∗∗∗∗^p < 0.0001. BALF, bronchoalveolar lavage fluid; CFU, colony forming units; i.n., intranasal; p.i., post-infection.

A short-term in vivo exposure to rmIL-13 was sufficient to “re-polarize” resident AMs from IL-13-deficient mice toward an M2 phenotype (Figure 5A) and reduce CXCL1 releases induced by *S. pneumoniae* (Figures 5B and 5C). Finally, the intranasal administration of rmIL-13 to *Il13*^−/−^ mice impaired bacterial clearance in lungs and blood, with bacterial counts being comparable to WT controls (Figures 5D and 5E). In summary, these data demonstrate that the lung immune environment at homeostasis is profoundly shaped by IL-13 at the expense of impaired anti-bacterial defenses.

### Resident ILC2s Are the Sole Source of IL-13 in Healthy Adult Lungs

**Figure 6:**
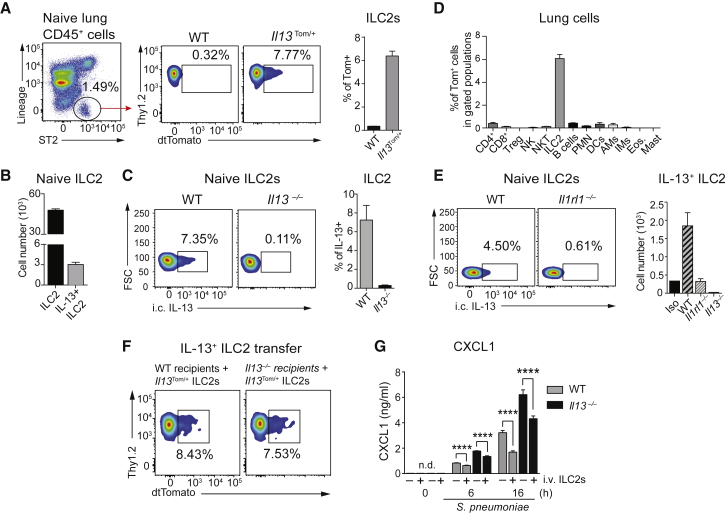
ILC2 Are the Only Cells Producing IL-13 in the Lung at Homeostasis (A) IL-13 expression in ILC2s (Lin^−^ ST2^+^ ICOS^+^ Thy1.2^+^ CD25^+^) assessed by flow cytometry in adult, naive *Il13*^*Tom/+*^ mice. Representative plots and percentage of tdTomato^+^ ILC2s are shown. (B and C) ILC2s and IL-13^+^ ILC2s were quantified by flow cytometry and intracellular staining for IL-13 in naive WT lungs. (B) Absolute numbers of total lung ILC2s (Lin^−^ ST2^+^ ICOS^+^ Thy1.2^+^ CD25^+^) and IL-13^+^ ILC2s. (C) Representative plots of IL-13^+^ ILC2s gated as in (A) and percentage of IL-13-producing ILC2s (right). (D) Lung cell populations were tested by FACS for IL-13 expression in healthy adult *Il13*^*Tom/+*^ mice. Gating strategies are shown in Table S2. (E) IL-13 production by lung ILC2s in WT, *Il1rl1*^−/−^, and *Il13*^−/−^ assessed by intracellular staining using flow cytometry (Iso, isotype control); representative plots and absolute numbers are depicted. (F and G) ILC2s were first expanded in lungs of *Il13*^*Tom/+*^ mice via i.n. administration of rmIL-33 (0.5 μg/50 μL for 5 days), and then sorted Tom^+^ ILC2s were transferred intravenous to WT and *Il13*^−/−^ mice. (F) Representative FACS plots showing the homing of *Il13*^*Tom/+*^ ILC2s in lungs 5 days after adoptive transfer. (G) AMs were isolated by bronchoalveolar lavage from WT and *Il13*^−/−^ recipients 5 days after adoptive transfer and in vitro stimulated with *S. pneumoniae* (MOI 100), and CXCL1 release was assessed by ELISA in supernatants. Data are representative of three (A–C), two (E–G), and one (D) independent experiments with four mice per group. Mean ± SEM are depicted; ^∗∗∗∗^p < 0.0001. BAL, bronchoalveolar lavage; i.c., intracellular; i.v., intravenous; FSC, forward scatter.

To determine the potential contribution of ILC2s to the AM phenotype, we evaluated the activity of lung ILC2s in adult mice using *Il13*^*Tom/+*^ reporter mice and intracellular cytokine staining. We found that ∼7%–8% of ILC2s in the lungs of naive adult mice (∼3 × 10^3^ cells) produced IL-13 (Figures 6A–6C). Notably, we excluded Th2 cells, eosinophils, mast cells, macrophages, NK cells, natural killer T (NKT) cells, and invariant natural killer T (iNKT) cells, which have all been shown to produce IL-13 in different lung pathological conditions (Kim et al., 2008, Price et al., 2010, Rijavec et al., 2011) as the source of IL-13 at steady state in healthy adult lungs (Figures 6D and S6A). In fact, ILC2s were the only cells expressing *Il13* in healthy adult lungs at homeostasis (Figure 6D), a finding we confirmed by intracellular staining for IL-13 (Figure S6B).

Constitutive IL-13 production by lung resident ILC2s did not depend on T or B cells in adult mice (Figures S6C–S6G). However, homeostatic IL-13 production depended on ST2 (Figure 6E) and less so on IL-25, another cytokine capable of inducing IL-13 production by ILC2s via IL17rb (Roediger and Weninger, 2015) (Figure S6H). The absolute number of lung ILC2s did not change in the absence of ST2 or IL17rb (Figure S6I). In summary, lung resident ILC2s are a constant and unique source of pulmonary IL-13 in healthy adult lungs at steady state.

To test if IL-13-producing ILC2s alone were sufficient to determine the responsiveness of AMs to *S. pneumoniae*, we adoptively transferred IL-33-expanded lung Tom^+^ ILC2s to WT and *Il13*^−/−^ mice (Figure 6F). We observed a significant reduction of *S.-pneumoniae*-induced CXCL1 secretion by AMs isolated from *Il13*^−/−^ recipients that received Tom^+^ ILC2s (Figure 6G). Of note, adoptively transferred ILC2s were also able to reduce the responsiveness of WT AM to *S. pneumoniae* in vitro. Collectively, these data show that pulmonary ILC2-derived IL-13 maintains lung resident AMs in an M2 state in healthy adult mice.

### ILC2s Maintain the M2 Polarization of AMs Early in Development and in Adult Lungs

**Figure 7:**
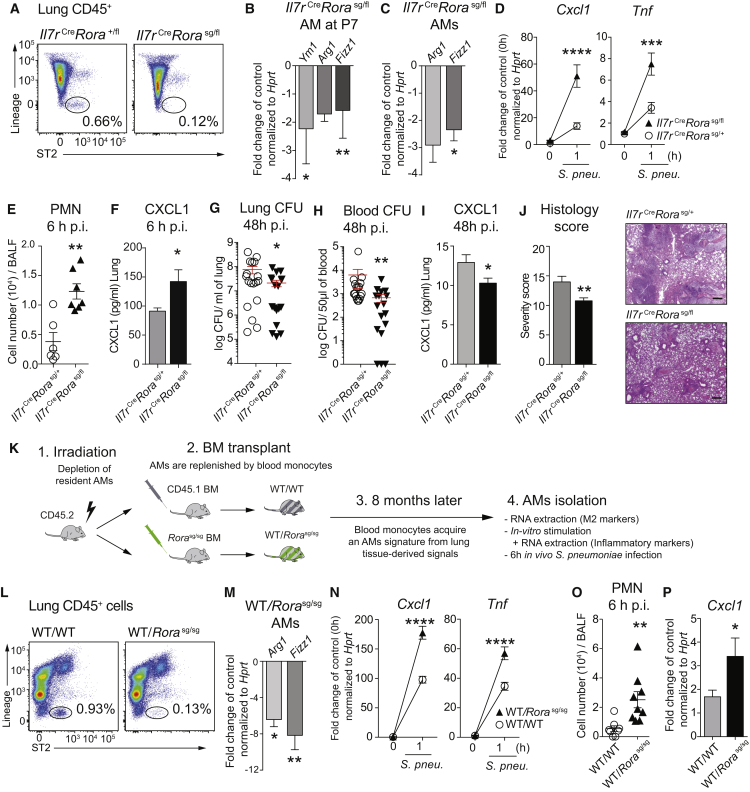
Lung Resident ILC2s Polarize Tissue Resident AMs toward an M2 Phenotype and Dampen Early Inflammatory Responses against Bacteria (A) Flow cytometry plots of ILC2s in naive *Il7r*^Cre^*Rora*^*sg/fl*^ mice and *Il7r*^*Cre*^*Rora*^*+/fl*^ controls. (B) AMs isolated by flow cytometry from healthy *Il7r*^*Cre*^*Rora*^*sg/fl*^ mice and controls at P7 and M2 markers evaluated by RT-PCR. (C and D) AMs isolated by bronchoalveolar lavage from healthy adult *Il7r*^*Cre*^*Rora*^*sg/fl*^ mice and controls. (C) M2 markers evaluated by RT-PCR. (D) Primary AMs stimulated for 1 hr with *S. pneumoniae* (MOI 100) to assess the induction of *Cxcl1* and *Tnf*. (E–J) *Il7r*^*Cre*^*Rora*^*sg/fl*^ mice and controls were i.n. infected with *S. pneumoniae* (10^5^ CFUs) and sacrificed after 6 hr (E and F) or 48 hr (G–J). (E) PMN influx on BALF cytospins. (F) CXCL1 induction in whole-lung homogenate. (G and H) CFUs in lung (G) and in blood (H). (I) CXCL1 induction in whole-lung homogenate. (J) H&E-stained lung sections were scored by a pathologist (see Experimental Procedures) (J, left). Representative H&E lung sections (J, right). Scale bars represent 180 μm. (K–N) CD45.2 recipients were lethally irradiated and transplanted with WT or *Rora*^*sg/sg*^ bone marrow and sacrificed 8 months later. (K) Experimental setup. (L) Representative FACS plots of ILC2s in healthy WT/WT and WT/*Rora*^*sg/sg*^ bone marrow chimeras. (M) AMs isolated via bronchoalveolar lavage and assessed for M2 markers by RT-PCR or (N) stimulated with *S. pneumoniae* (MOI 100) to evaluate *Cxcl1* and *Tnf* induction. (O and P) WT/WT and WT/*Rora*^*sg/sg*^ chimeras were infected with 10^5^ CFUs *S. pneumoniae* and sacrificed after 6 hr to assess (O) PMN influx and (P) *Cxcl1* induction in lung tissue. Data are representative of at least two independent experiments with four (A–D, M, and N) and seven or eight (E–J, O, and P) mice per group. Data in (G) and (H) are pooled from two independent experiments. Mean ± SEM are depicted; ^∗^p ≤ 0.05, ^∗∗^p < 0.01, ^∗∗∗^p < 0.001, and ^∗∗∗∗^p < 0.0001. BALF, bronchoalveolar lavage fluid; CFU, colony-forming units; p.i., post-infection; PMN, polymorphonuclear cells.

We then asked if the congenital absence of ILC2s would mirror the phenotype observed in *Il13*^−/−^ mice. AMs extracted from newborn (P7) and adult *Il7r*^*Cre*^*Rora*^*sg/fl*^ mice, congenitally deficient in lung resident ILC2s (Oliphant et al., 2014) (Figure 7A), showed a reduced expression of M2 markers (Figure 7B, 7C and S7A) and increased *Cxcl1* and *Tnf* expression when stimulated with *S. pneumoniae* (Figure 7D). Infection of *Il7r*^*Cre*^*Rora*^*sg/fl*^ mice with *S. pneumoniae* resulted in increased neutrophil influx and higher lung CXCL1 levels 6 hr post-infection (Figures 7E, 7F, and S7B). This translated into an improved bacterial clearance with reduced systemic dissemination of pneumococci (Figures 7G and 7H), lower pulmonary CXCL1 levels (Figure 7I), and less severe lung infiltrates in *Il7r*^*Cre*^*Rora*^*sg/fl*^ mice 48 hr after infection (Figure 7J). We concluded that the congenital absence of ILC2s impacted on the M1 versus M2 polarization of AMs in neonatal and adult mice, with implications on the ability to fight bacterial lung infections.

We then asked to which degree ILC2s might contribute environmental signals to shape the functionality of bone-marrow-derived AMs (Lavin et al., 2014) and generated bone marrow chimeras using ILC2-deficient *Rora*^*sg/sg*^ mice as donors (Wong et al., 2012) (Figures 7K and 7L). AMs isolated from WT/*Rora*^*sg/sg*^ chimeras expressed lower levels of the M2 markers *Arg1* and *Fizz1* (Figure 7M) and higher levels of *Cxcl1* and *Tnf* in response to *S. pneumoniae* (Figure 7N). In vivo, WT/*Rora*^*sg/sg*^ chimeras exhibited an augmented early inflammatory response upon pneumococcal infection (Figures 7O, 7P, and S7C). Collectively, lung ILC2s convey important cues that maintain quiescence by shaping the functional state of lung macrophages at homeostasis.

## Discussion

With the first breath, lungs are suddenly exposed to the external environment, therefore requiring regulatory forces in place to avoid continuous inflammatory reactions to environmental stimuli. Here, we show a perinatal wave of IL-33-mediated expansion and activation of ILC2s, resulting in an IL-13-driven polarization of newly differentiating AMs to an M2 phenotype. This exerts important homeostatic functions that contribute to a quiescent lung environment shortly after birth and throughout adult life.

AEC2 are the main source of IL-33, as shown earlier (Hardman et al., 2013, Pichery et al., 2012) and further confirmed by a recent study of developing AEC2 in embryonic lungs (Treutlein et al., 2014). Even though the mode of homeostatic IL-33 release remains to be elucidated, the mechanical stress induced by physiological ventilation possibly contributes to pulmonary IL-33 (Martin and Martin, 2016), and we discovered that exposing E19 lungs to negative pressure was sufficient to induce IL-33. Moreover, the release of bioactive IL-33 from living cells upon encounter of environmental allergens, extracellular ATP, or mechanical stress has been reported (Chen et al., 2015, Kakkar et al., 2012, Kouzaki et al., 2011, Sanada et al., 2007).

IL-33 and ILC2s are increasingly recognized as fundamental regulators of tissue homeostasis (Molofsky et al., 2015, von Moltke and Locksley, 2014). As such, recent reports described an IL-33-driven, ILC2-dependent mechanism for adipose tissue homeostasis, which involves the presence of eosinophils and M2 macrophages (Lee et al., 2015, Qiu et al., 2014). Excitingly, perinatal IL-33 induction was recently found to license adipocytes for uncoupled respiration and thermoregulation after birth (Odegaard et al., 2016).

The IL-33- and ILC2-dependent physiological type 2 milieu that we describe might play a role in the reportedly exaggerated airway hyperreactivity upon house dust mite exposure in newborns and strengthens the concept of a “window of immune development” (Gollwitzer et al., 2014). In fact, while this article was under revision, a report demonstrated a casual link between perinatal IL-33 induction and asthma (de Kleer et al., 2016). Here, we propose a unique and homeostatic role for ILC2s in shaping the lung immune environment in early life, as the appearance of activated ILC2s around P3 gradually de-activated AMs.

While type 2 responses, as seen upon helminth infections, are known to impair defenses against mycobacteria (Monin et al., 2015, Salgame et al., 2013), we now report that even homeostatic type 2 conditions impact on lung immunity, illustrated by reduced lung inflammation upon LPS challenge and a delayed clearance of medically important lung pathogens such as *S. pneumoniae*.

Our experiments in mice congenitally deficient in ILC2s corroborated the concept that ILC2s affected the AM phenotype from birth until adult life. Analysis of bone marrow chimeras using *Rora*^*sg/sg*^ mice further demonstrated that pulmonary ILC2s provided essential, tissue-specific signals to even polarize bone marrow precursors that arrive in lungs. Supporting our notion that ILC2s contribute to the in vivo phenotype of AMs, a recent publication identified tissue-specific transcriptional signatures of resident macrophages, and found AMs to be characterized by two IL-13- and IL-5-inducible genes, namely *Ym1* (*Chi3l3*) and *Car4*, respectively (Lavin et al., 2014).

In conclusion, we show that IL-33-driven ILC2 activation dominates the lung milieu early after birth by inducing a type-2 immune environment. Lung resident ILC2s are major contributors to the phenotype and function of tissue resident AMs at homeostasis, favoring a quiescent immune environment. While this effect might prove beneficial at steady state and upon sterile lung injury, it comes at the expense of a delayed response to the common lung pathogen *S. pneumoniae.*

## Experimental Procedures

### Mice

*Il13*^−/−^ (McKenzie et al., 1998), *Il13* ^*tdTomato/+*^ (Barlow et al., 2012) (referred as *Il13*^*Tom/+*^)*, Il1rl1*^−/−^ (Townsend et al., 2000), *Il5*^−/−^ (Kopf et al., 1996), *Rag2*^−/−^ (Shinkai et al., 1992), *Il7r*^*Cre*^ (Schlenner et al., 2010), *Rora*^*+/fl*^ (Oliphant et al., 2014), and Staggerer *Rora*^*sg/+*^ mice (Jackson Laboratories) were on a C57BL/6 background. We obtained *Il7r*^*Cre*^*Rora*^*sg/fl*^ mice (experimental) or *Il7r*^*Cre*^*Rora*^*+/fl*^ littermate controls by crossing *Il7r*^*Cre*^ with *Rora*^*+/fl*^ and *Rora*^*sg/+*^. *Il33*^*Cit/+*^ (Hardman et al., 2013) mice and *Il5*^*Cer/+*^ mice (Saunders et al., 2016) were on a BALB/c background. Mice were bred in a specific pathogen-free (SPF) facility and all mice were matched for age, gender and background in individual experiments. All animal experiments were approved by the Austrian Federal Ministry of Sciences and Research (BMWFW-66.009/0122-II/3b/2013) and the UK Home Office.

### Isolation, Culture, and Stimulation of AMs

AMs from newborn mice were isolated by cell sorting using an FACSAria II (BD Biosciences) by gating on viable CD45^+^F4/80^+^CD11b^low^CD11c^+^ SiglecF^+^ Ly6C^−^ cells. In adult mice, AMs were isolated by bronchoalveolar lavage followed by cell adhesion. Purity of isolated AM with both methods was consistently >95%. AMs were stimulated in RPMI containing 3% fetal calf serum (FCS) with heat-inactivated *S. pneumoniae* at a MOI 100 or *S. aureus* LTA (10 μg/mL). In Figures 3E–3G, 4A–4D, 5A–5C, 7B–7D and 7M, and 7N, cells were pooled from three or four mice per group and analyzed in technical quadruplicates.

### Cytokine Administration

Recombinant mouse IL-13 and IL-33 were purchased from BioLegend. Anesthetized mice were treated daily with rmIL-13 (6 ng/50 μL NaCl for 3 consecutive days) or rmIL-33 (0.5 μg/50 μL NaCl for 5 consecutive days). Mice were sacrificed 1 hr after the last administration.

### Adoptive Transfer of ILC2s

Lung ILC2s were FACS purified as defined by lineage^−^ (CD3a, CD4, CD8a, CD19, CD11c, CD11b, Gr1, FcεR1, CD49b), Thy1.2^+^ ST2^+^ ICOS^+^ and Tom^+^ from *Il13*^Tom/+^ mice that had been treated intranasally (i.n.) with rmIL-33 for 5 days. Cells were transferred intravenously to *Il13*^−/−^ or WT mice recipients (1 × 10^5^ cells per mouse) and assessed for localization in lungs 5 days later.

### Generation of Bone Marrow Chimeras

6-week-old CD45.2 *Rora*^*sg/sg*^ or WT littermates served as bone marrow donors. CD45.1 recipients were irradiated (9 Gy) and reconstituted on the same day with 2 × 10^6^ bone marrow cells per recipient by intravenous injection. Mice were analyzed for reconstitution and absence of lung resident ILC2s after 8 months.

### Murine Pneumonia Model

Mice were infected i.n. with 10^5^ CFUs *S. pneumoniae* serotype 3 (ATCC 6303) as described (Sharif et al., 2014, Warszawska et al., 2013), or with 5 × 10^7^ CFUs *S. aureus* (USA300). Acute lung injury was induced by administration of 100 ng LPS i.n. (*E. coli* O55:B5). BALF was collected, cells were counted with an automated cell counter (Z2 Coulter Counter, Beckman), and Giemsa-stained cytospin preparations were used for differential cell counts. Lung tissues were homogenized in sterile saline using a Precellys 24TM (Peqlab), and lung colony-forming units (CFUs) were determined by 10-fold serial dilutions of homogenates on blood agar plates. An aliquot of lung homogenates was incubated in RA1 buffer (Macherey-Nagel) containing 10% of beta-mercaptoethanol (Calbiochem) and stored at −80° for RNA extraction. The remaining lung homogenates were incubated in Greenberger lysis buffer as described previously (Sharif et al., 2014), and supernatants were stored at −20°C until cytokines were assayed.

### Pneumonia Severity Score

Paraffin-embedded lung sections were stained with H&E and scored by a trained pathologist who was blinded to experimental groups. The final pneumonia score was the sum of the following parameters: severity of pleuritis, interstitial inflammation, edema, and thrombi formation were scored as 0 = absent, 1 = mild, 2 = moderately severe, 3 = severe; bronchitis was scored as 1 if present; endotheliitis was scored as 0 = absent, 2 = present, 3 = present with endothelial wall necrosis; the existence of a lobar confluent infiltrate was scored as 1, and a score of 0.5 was added for every infiltrate covering 10% of the lung area.

### Statistical Analysis

Data are expressed as mean ± SEM. Statistical significance in two-group comparisons was assessed with an unpaired Student’s t test. When indicated, a Mann-Whitney *U* test was used for analysis of nonparametric data. For multivariable comparisons we performed a one-way ANOVA followed by Sidak’s multiple comparison test. Results were analyzed with Graph Pad Prism software version 6, and a p < 0.05 was regarded as statistically significant.

## Author Contributions

S. Saluzzo and S.K. conceived the study. A.N.J.M. hosted S. Saluzzo, contributed to experimental design, and provided critical reagents. S. Saluzzo, A.-D.G., B.M.J.R., R.M., S. Scanlon, P.S., K.L., A.H., O.S., A.K., J.M.W., and H.J. performed the experiments or contributed to experimental design, reagents, and analysis. I.M. scored histological slides. S. Scanlon performed the immunofluorescence experiments. R.M. analyzed the newborns alveolarization. A.-D.G. and B.M.J.R. contributed equally to this work. S. Saluzzo and S.K. wrote the manuscript with contributions from A.N.J.M., B.M.J.R., O.S., and S. Scanlon. A.N.J.M and S.K. are joint senior authors.

## Supplemental Information

Document S1. Supplemental Experimental Procedures, Figures S1–S7, and Tables S1–S3

Document S2. Article plus Supplemental Information
