# Supplementary material for: First-Breath-Induced Type 2 Pathways Shape the Lung Immune Environment
Source: Cell Rep. 2017 Feb 21;18(8):1893–905. doi: 10.1016/j.celrep.2017.01.071 (PMC5329122; doi:10.1016/j.celrep.2017.01.071)
Supplement: Document S1. Supplemental Experimental Procedures, Figures S1–S7, and Tables S1–S3 [file mmc1.pdf]

Cell Reports, Volume *18*

Supplemental Information

First-Breath-Induced Type 2 Pathways

Shape the Lung Immune Environment

Simona Saluzzo, Anna-Dorothea Gorki, Batika M.J. Rana, Rui Martins, Seth Scanlon, Philipp Starkl, Karin Lakovits, Anastasiya Hladik, Ana Korosec, Omar Sharif, Joanna M. Warszawska, Helen Jolin, Ildiko Mesteri, Andrew N.J. McKenzie, and Sylvia Knapp

Supplemental Figures and Legends

Figure S1

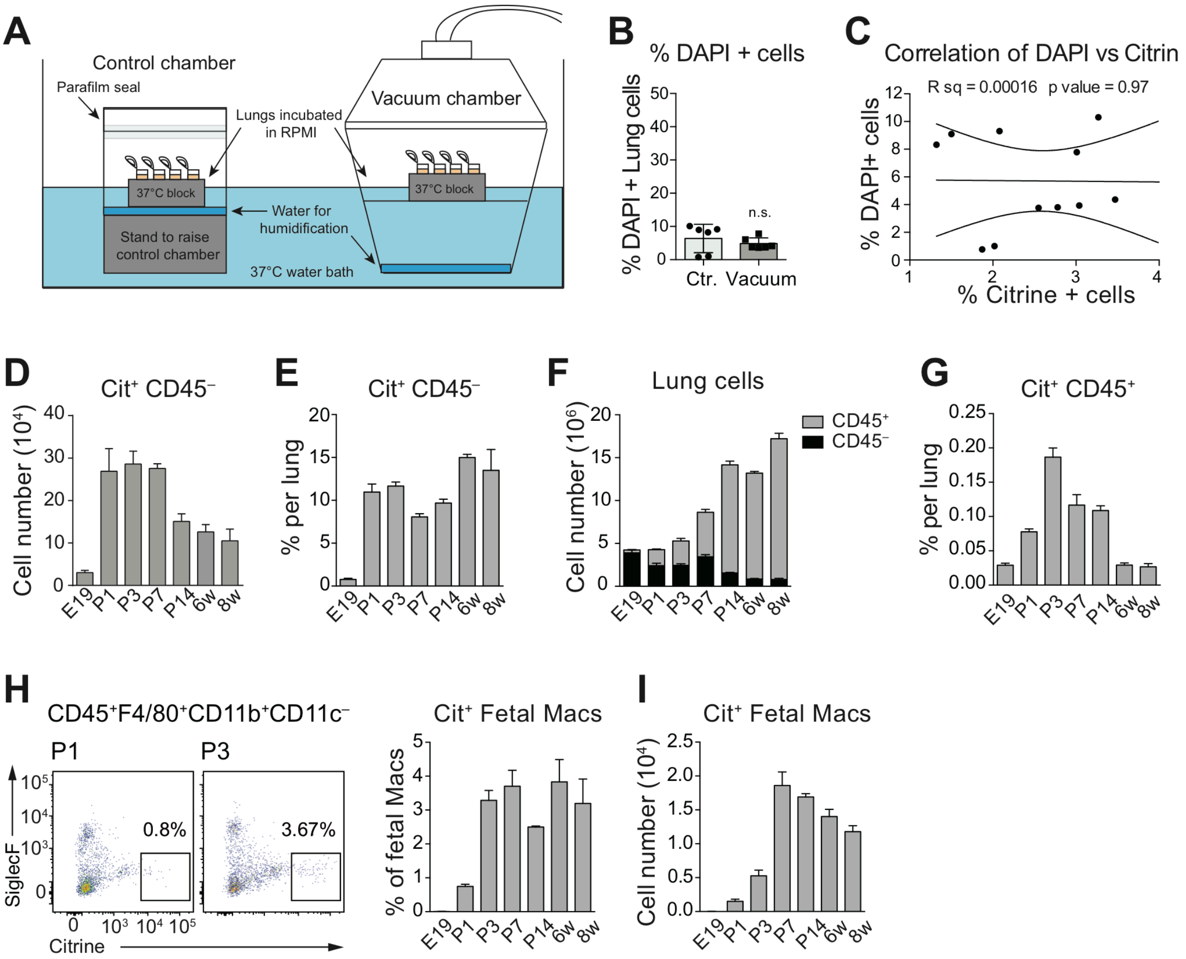

Figure S1. Integrated analysis of Citrine expression in lung cells of newborns and upon negative pressure. Related to Figure 1.

(A) Experimental set-up. Embryonic lungs (P19) were placed at 37°C and subjected to a negative pressure for 6h (see experimental procedures) (referring to Figure 1C, D, E).

(B) Viability of lung cells assessed by DAPI staining (referring to Figure 1C, D, E).

(C) Linear regression analysis of viability and IL-33 expression (Citrin) of embryonic lung cells subjected to negative pressure (vacuum) for 6h (referring to Figure 1C, D, E).

(D) Absolute numbers of Cit^{+} $CD45^{-}$ lung cells (referring to Figure 1F lower panel and 1G).

(E) Proportion of $CD45^{-}$ cells that up-regulate Citrine at indicated time points (referring to Figure 1F lower panel)

(F) Relative contribution of $CD45^{+}$ and $CD45^{-}$ cells to total lung cell numbers from E19 to 8 weeks of age (referring to Figure 1F upper panel).

(G) Quantification of Citrine expression among CD45+ cells in newborn mice.

(H) Left: blots showing expression of Citrine among fetal macrophages gated as $CD45^{+}$ $F4/80^{+}$ $CD11b^{+}$ $CD11c^{-}$ $SiglecF^{-}$. Right: quantification of Citrine expression among fetal macrophages.

(I) Absolute numbers of lung fetal macrophages gated as in (H) at indicated time points.

Graph bars represent mean ± SEM. Data are representative of two independent experiments with 3-4 mice per time point.

Figure S2

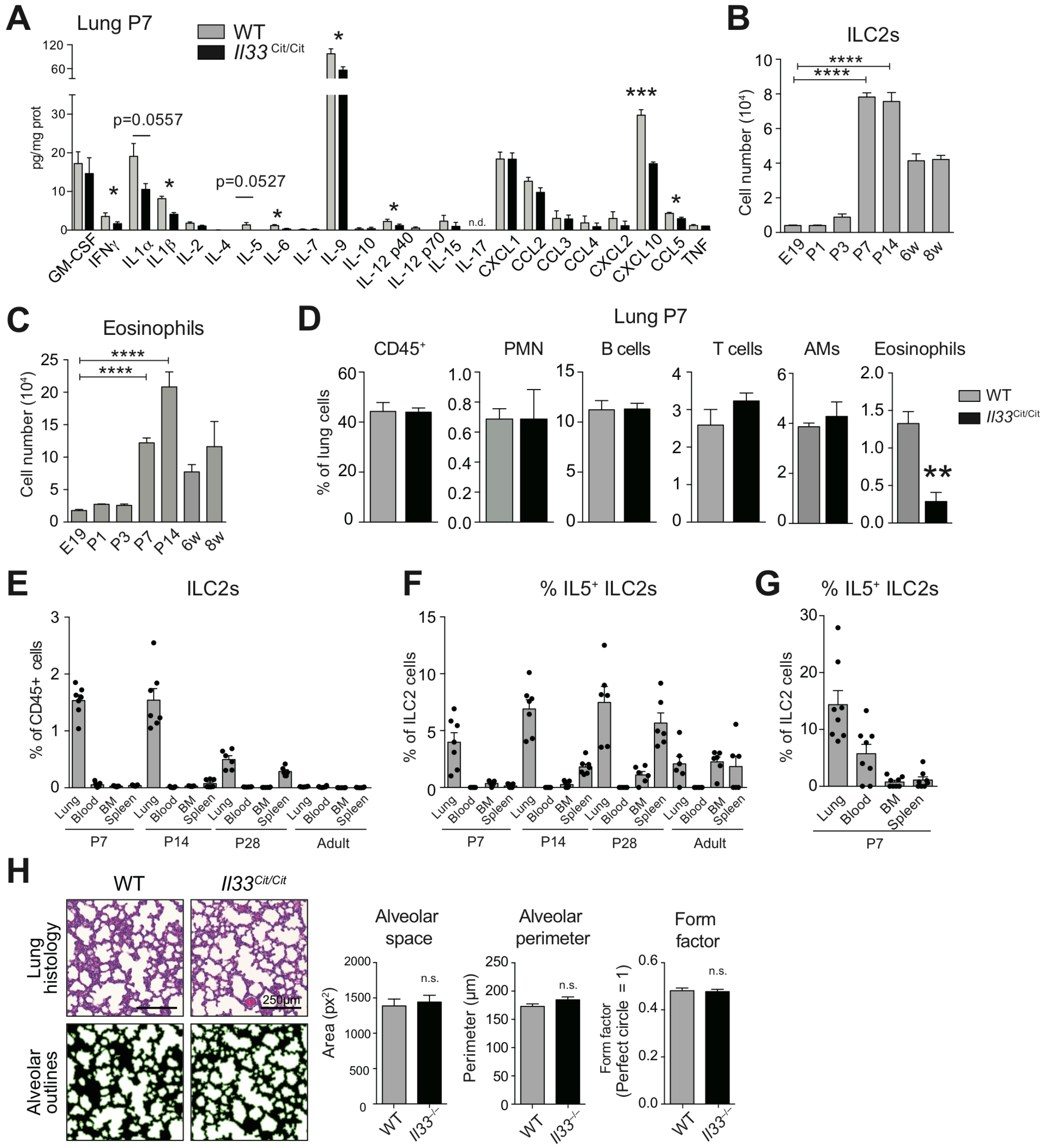

Figure S2. Integrated analysis of *Il33*$^{Cit/Cit}$ mice on P7, postnatal eosinophil expansion and perinatal alveolarization. Related to Figure 2.

(A) Absolute values of multiplex ELISA (referring to Figure 2A).

(B) Absolute numbers of lung ILC2s between E19 and week 8, (referring to Figure 2B).

(C) Absolute numbers of lung eosinophils between E19 and week 8, (referring to Figure 2E).

(D) Analysis of lung immune cell populations in WT and *Il33*$^{Cit/Cit}$ mice, depicted is the percentage of total lung cells, (referring to Figure 2F). PMN = polymorphonuclear cells gated as CD45$^+$ Ly6G$^+$; B cells = CD45$^+$ CD19$^+$; T cells = CD45$^+$ CD3$^+$; AMs = CD11b$^-$ SiglecF$^+$ CD11c$^+$; eosinophils = CD11b$^+$ SiglecF$^+$ CD11c$^-$.

(E, F) Proportion of ILC2s (among CD45$^+$cells) and IL5-expressing ILC2s (*Il5*$^{Cer/+}$ mice) in indicated body compartments between P7 and adulthood.

(G) Quantification of IL5 production by ILC2s assessed by i.c. cytokine stain, (referring to Figure 2H).

(H) Lung alveolarization was quantified on H&E stained lung sections from WT and *Il33*$^{Cit/Cit}$ mice at P7 using the automatic image analysis software CellProfiler. For details see Supplemental experimental procedures. Graph bars represent mean ± SEM. *$p < 0.05$, ***$p<0.001$, ****$p < 0.0001$. Data are representative of two independent experiments with 4 mice per group and/or time point.

Figure S3

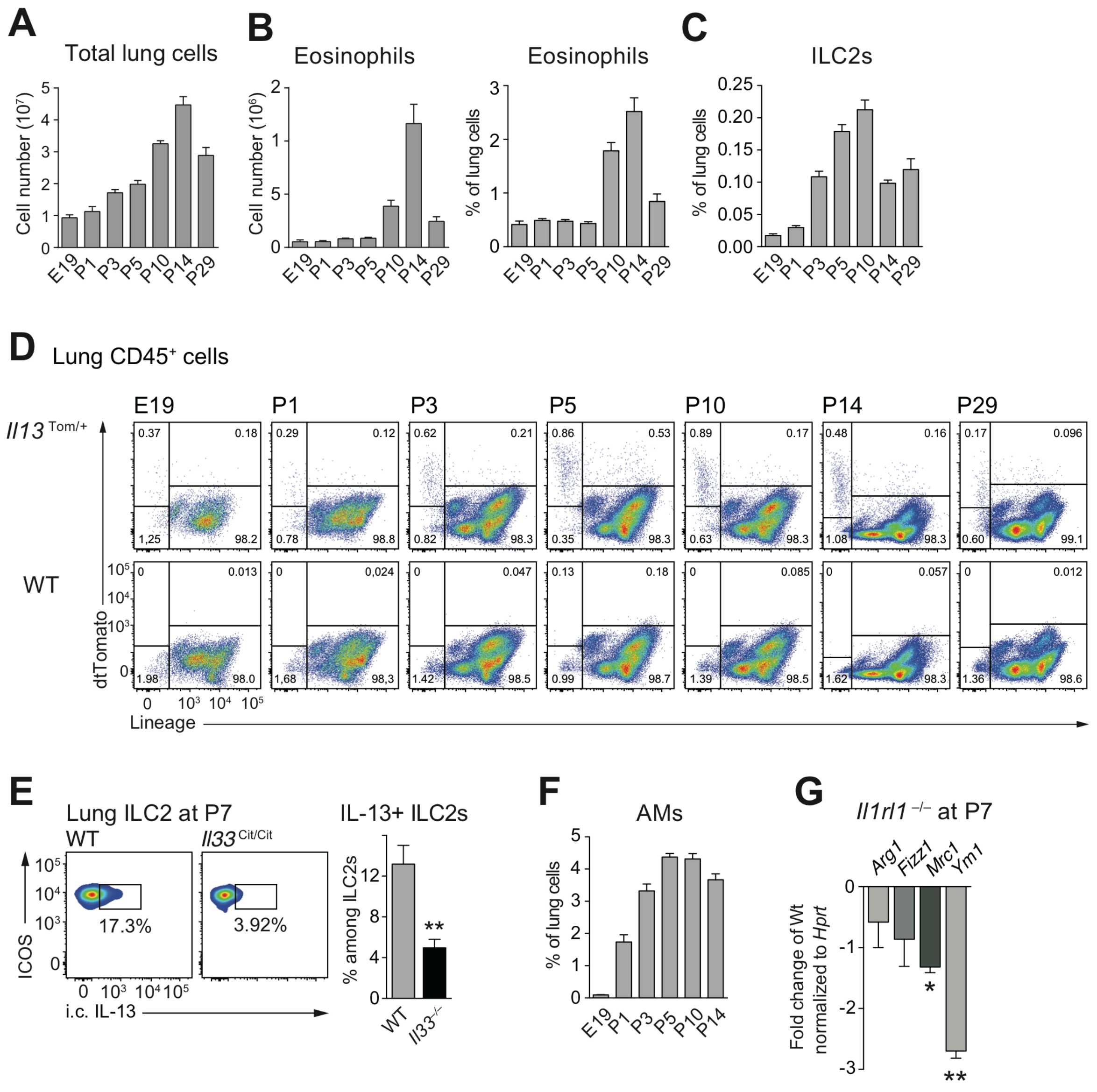

Figure S3. Lin⁻cells are the only IL-13 expressing cells in the first two weeks after birth. Related to Figure 3.

(A-C) Quantification of lung ILC2s and eosinophils at the indicated time points in *Il13*$^{Tom/+}$ mice. Of note, *Il13*$^{Tom/+}$ mice, which are on a C57BL/6 background, show the same cell influx dynamic as *Il33*$^{Cit/+}$ mice, which are on a Balb/c background, (referring to Figure 3A-D). (A) Absolute number of total lung cells. (B) Absolute numbers and percentages of eosinophils gated as in Figure 2E. (C) Percentage of ILC2s, gated as in Figure 2B.

(D) FACS of lung cells gated for Lineage (CD3, CD4, CD8, CD19, F4/80, CD11b, CD11c, FceRI, NK1.1, Ly6C/G) and *Il13*^{dtTomato} expression at indicated time points.

(E) IL-13 expression in lung ILC2s at P7 quantified by i.c. cytokine stain.

(F) Percentage of AMs gated as in Figure 3C, (referring to Figure 3C-D).

(G) AMs (F4/80^{+}CD11b^{-}CD11c^{+}SiglecF^{+}) were sorted on P7 from WT and *Il1rl1*$^{-/-}$ mice and M2 markers were assessed by RT-PCR.

Data are representative of two (A-F) and one (G) independent experiments with 3-4 mice per group. Bars represent mean ± SEM; *$p < 0.05$; **$p < 0.01$.

Figure S4

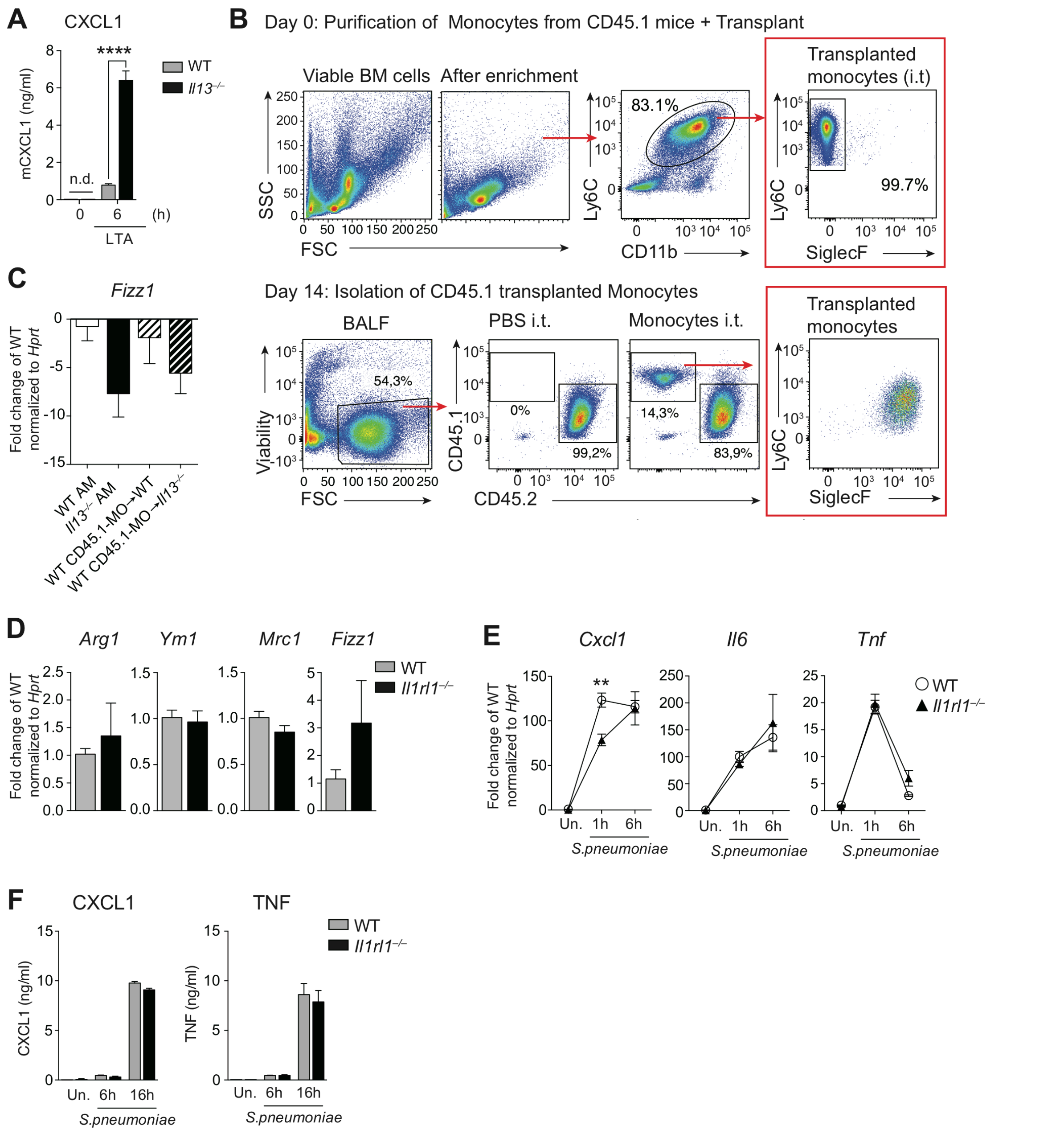

Figure S4. BM derived monocytes assume an AMs phenotype 2 weeks upon intratracheal transplant. *Il1rl1* alone plays no significant role in AM polarization in adult mice. Related to Fig 4.

(A) AMs from WT and *Il13*$^{-/-}$ mice were isolated by BAL and *in vitro* stimulated with LTA (10µg/ml). CXCL1 was quantified in supernatants, (refers to Figure 4B-C).

(B) FACS plots representing bone marrow derived monocytes isolated from WT CD45.1 mice and transferred i.t. into WT or *Il13*$^{-/-}$ mice. Two weeks later transferred monocytes showed upregulation of SiglecF, (refers to Fig 4D).

(C) *Fizz1* expression was quantified in FACS-sorted AMs upon monocytes transplant, (refers to Fig 4D.

(D, E) AMs isolated via BAL from WT and *Il1rl1*$^{-/-}$mice analyzed for M2 markers by RT-PCR (D), or *in vitro* stimulated with *S. pneumoniae* (MOI 100) and analyzed for cytokine expression by RT-PCR (E). Values were normalized to HPRT and are expressed as fold-change versus WT at t = 0h.

(F) AMs were isolated as in (D), and stimulated *in vitro* with *S. pneumoniae* (MOI 100) and CXCL1 and TNF protein levels were quantified in supernatants by ELISA.

Data are representative of at least two independent experiments with four (A and D-F) and 6-7 (B, C) mice per group and/or time point. Mean ± SEM are depicted; $**p < 0.01$, $****p < 0.0001$. I.t. = intra-tracheally; MOI = multiplicity of infection; BAL = bronchoalveolar lavage.

Figure S5

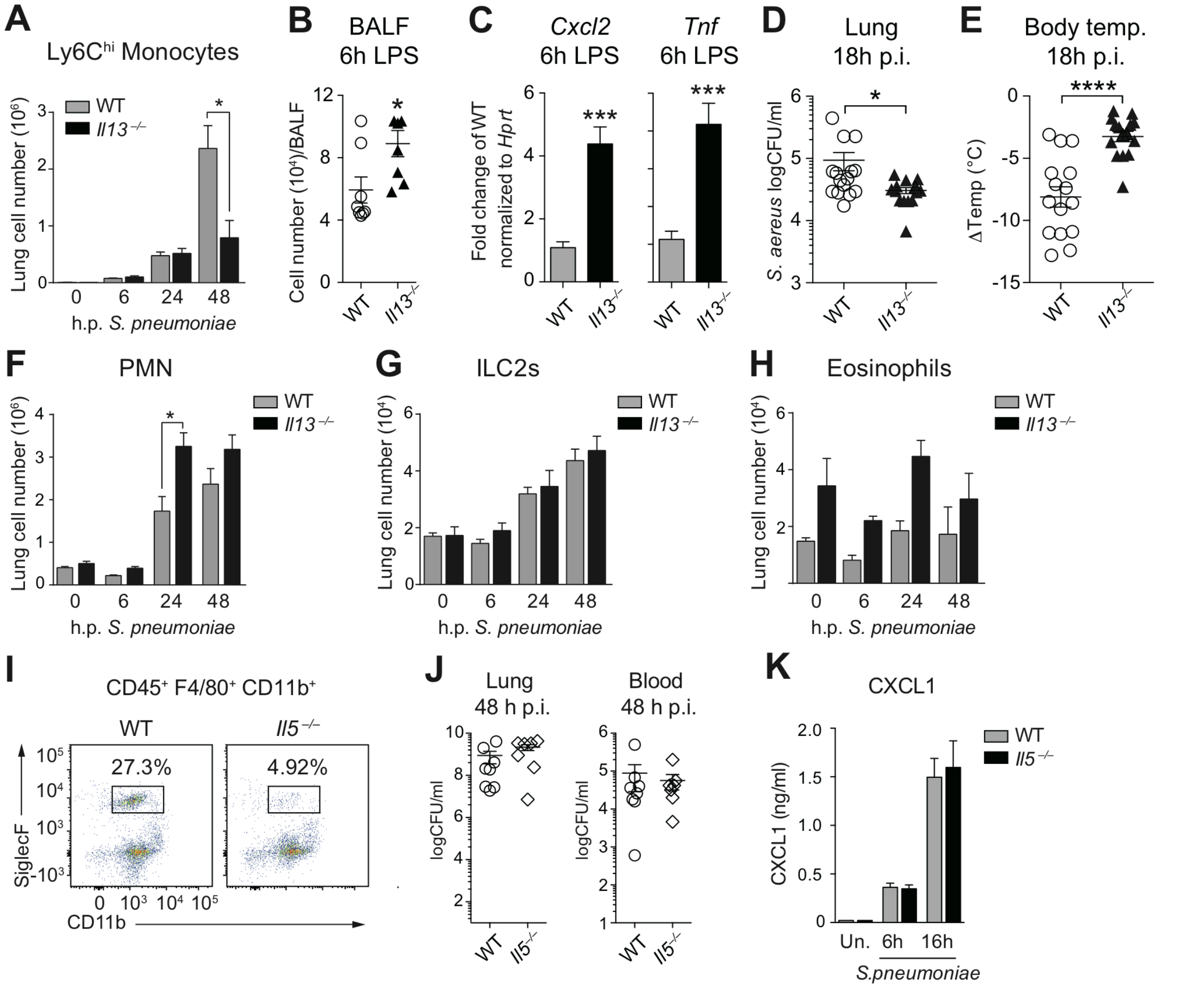

Figure S5. Broader role of IL-13 in the inflammatory response to LPS and *S. aureus*. Eosinophils do not impact on AM polarization, or responses to *S. pneumoniae*. Related to Fig 4.

(A) Numbers of lung Ly6C^{hi} monocytes (F4/80^{+} CD11b^{+} Ly6C^{hi}) during *S. pneumonia* infection, (refers to Figure 4E-K).

(B-C) Mice were treated i.n. with LPS (100ng/50µl) and harvested 6h later to assess cell influx in BALF (B) and cytokine expression in BALF (C).

(D-E) Mice were infected i.n. with *S. aureus* and assessed 18h later for body temperature (E), and harvested to quantify lung CFUs (D) .

(F-H) Absolute numbers of (F) PMN (SSC^{hi} FSC^{hi} CD11b^{+} Ly6G^{+}), (G) ILC2s (Lin^{-} ST2^{+} ICOS^{+} CD25^{+} Thy1.2^{+}), (H) eosinophils (CD11b^{+} F4/80^{+} SSC^{hi} CD11c^{-} SiglecF^{+}) during the course of *S. pneumoniae* infection; (refers to Figure 4E-K).

(I) FACS plots illustrating lung eosinophil numbers in healthy WT and *Il5*$^{-/-}$ mice.

(J) WT and *Il5*$^{-/-}$ mice were i.n. infected with 10^5 CFU *S. pneumoniae* and sacrificed after 48h. CFU counts were assessed in lung homogenates (left) and blood (right).

(K) BAL isolated AMs from WT and *Il5*$^{-/-}$ mice were *in vitro* stimulated with *S. pneumoniae* (MOI 100). CXCL1 was quantified in supernatants.

Graph bars represent mean ± SEM. *p < 0.05, ***p < 0.001, ****p < 0.0001. Data are representative of two independent experiments with 4 (A, I and K) and at least 8 (B-H, and J) mice per group and/or time point.

Figure S6

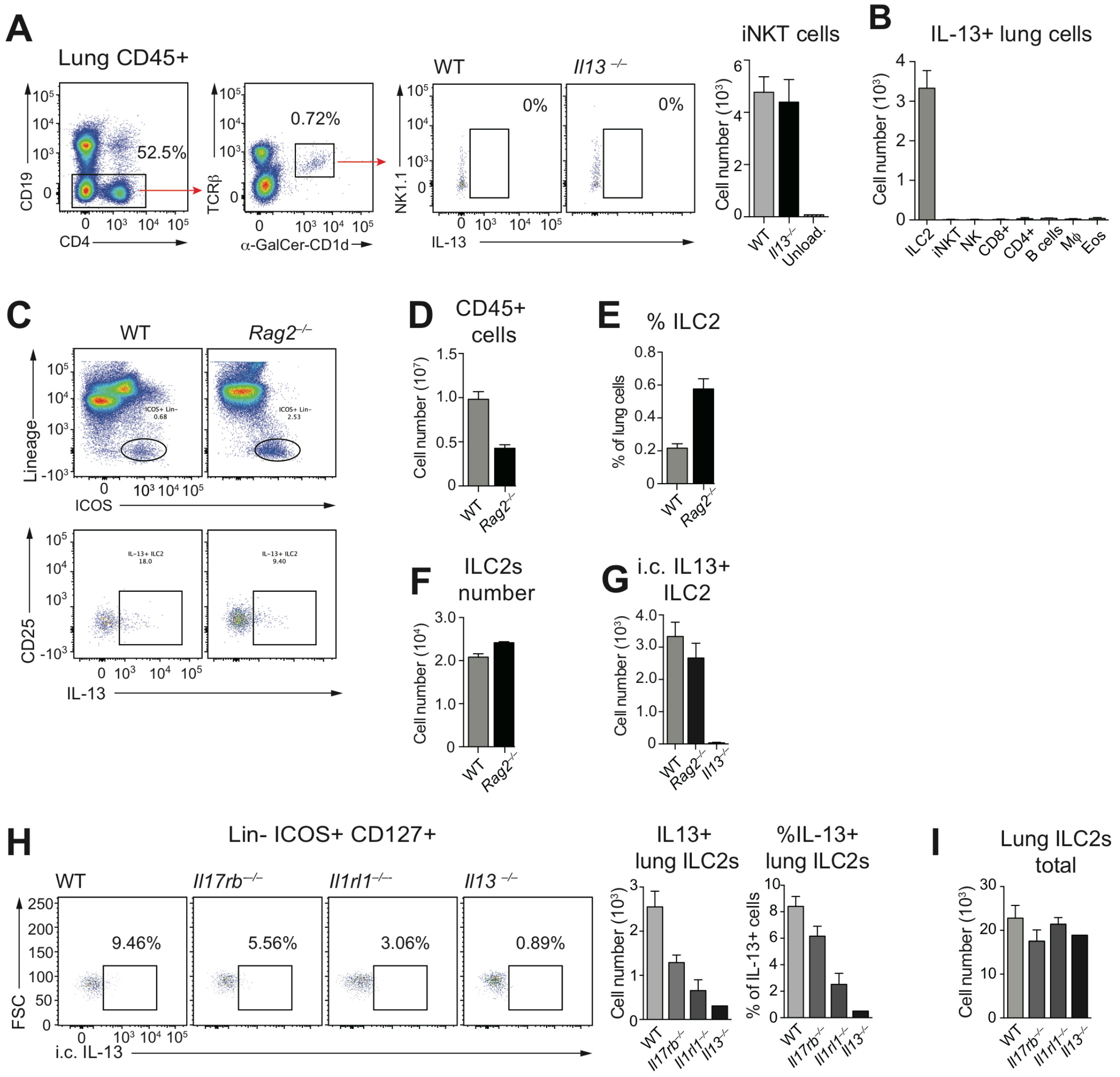

Figure S6. iNKT cells are not a source of IL-13 at homeostasis. Baseline ILC2-derived IL-13 expression does not depend or on B and T cells, but partially on *Il17rb*. Related to Figure 6.

(A) FACS gating strategy for iNKT cells ($CD19^{-}CD4^{+/-}TCR\beta^{+}$ α-GalCer-CD1d $dimer^{+}NK1.1^{+}$). Unloaded CD1d dimer was used as negative control. Right panel: absolute number of iNKT cells in naive adult lungs. IL-13 production was assessed by i.c. staining.

(B) Absolute number of IL-13 secreting lung cells at homeostasis (cells gated as in Figure 6D). IL-13 production was assessed by i.c. staining. iNKT cells were gated as in Figure S6A.

(C-G) ILC2s were assessed for intracellular IL-13 production in naive WT and *Rag2*$^{-/-}$ mice at homeostasis. (C) Representative plots of ILC2s gating strategy. (D) Absolute lung cell number. (E) Percentage of ILC2s out of total lung cells. (F) Absolute number of ILC2s. (G) Absolute number of IL-13 expressing ILC2s.

(H) Representative FACS plots of ILC2s ($Lin^{-}ST2^{+}ICOS^{+}Thy1.2^{+}CD25^{+}$) assessed for IL-13 expression via i.c. staining in indicated mouse strains at homeostasis. Right: Absolute number and percentage of IL-13 secreting ILC2s.

(I) Absolute number of lung ILC2s at steady state in indicated mouse strains.

Single cells were pre-gated for viability and CD45+ expression. Graph bars represent mean ± SEM. Data are representative of at least two independent experiments with 4 mice per group. i.c. = intracellular.

Figure S7

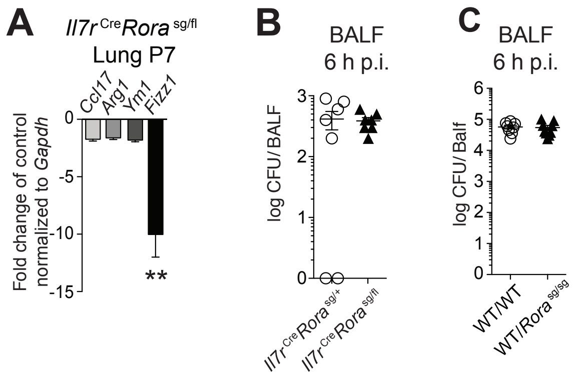

Figure S7. Reduced M2 marker expression in lungs of neonatal ILC2-deficient mice. Related to Fig 7.
(A) M2 markers were assessed by RT-PCR in whole lung homogenates of naive *Il7r*^{Cre}*Rora*$^{sg/fl}$ mice as compared to *Il7r*^{Cre}*Rora*$^{+/fl}$ controls on postnatal day 7 (P7), (refers to Figure 7B).
(B, C) BALF CFU in indicated mouse strains 6h post i.n. infection with *S. pneumoniae*, (refers to Figure 7E-J, and Figure 7O-P, respectively).
Graph bars represent mean ± SEM. **p < 0.01. Data are representative of at least two independent experiments with 4 mice per group (A) or 7-8 mice per group (B-C).

TABLES

Table S1. List of anti-mouse antibodies used in the study. Related to Figure 1-4 and 6-7.

Ab	Fluorochrome	Clone ID	Manufacturer	Isotype
B220	PerCP/Cy5.5	RA3-6B2	eBioscience	Rat IgG2a
CD45	V500	30-F11	BD Biosciences	Rat IgG2b
CD45	BV510	30-F11	Biolegend	Rat IgG2b
CD45.1	Pacific Blue	A20	Biolegend	Mouse IgG2a
CD45.2	PE	104	Biolegend	Mouse IgG2a
CD3	FITC, eFluor450	17A2	Biolegend	Rat IgG2b
CD3	PE/Cy7	145-2C11	Biolegend	Hamster IgG
CD4	FITC, PE/Cy7	GK1.5	eBioscience	Rat IgG2a
CD4	AF700, PE/Cy7, eFluor450	RM4-5	eBioscience	Rat IgG2a
CD8a	FITC, eFluor450, PE/Cy7	53-6.7	Biolegend or eBioscience	Rat IgG2a
CD11b	APC, FITC, AF700, PercP/Cy5.5, PE/Cy7, Pacific Blue	M1/70	Biolegend or eBioscience	Rat IgG2b
CD11c	FITC	HL3	BD Biosciences	Hamster IgG1
CD11c	AF700, PE/Cy7, BV421, AF647	N418	Biolegend or eBioscience	Hamster IgG
CD19	FITC, PE/Cy7	eBio1D3	eBioscience	Rat IgG2a
CD19	PE/Cy7, BV421, AF700	6D5	Biolegend	Rat IgG2a
CD31	PE/Cy7	390	eBioscience	Rat IgG2a
CD49b	FITC, Pacific Blue, PE/Cy7	DX5	Biolegend or eBioscience	Rat IgM
CD127	APC, PE/Cy7	A7R34	Biolegend	Rat IgG2a
CD138	APC	281-2	Biolegend	Rat IgG2a
c-Kit	AF700	ACK2	eBioscience	Rat IgG2b
EpCam	PE/Cy7	G8.8	Biolegend	Rat IgG2a
F4/80	BV421, BV785, FITC, PE/Cy7, APC, PerCp/Cy5.5	BM8	Biolegend or eBioscience	Rat IgG2a
FceRIa	FITC, Pacific Blue, PE/Cy7, PE	MAR-1	Biolegend or eBioscience	Hamster IgG
Foxp3	APC	FJK-16s	eBioscience	Rat IgG2a
Gr-1	FITC, BV421, PE/Cy7	RB6-8C5	Biolegend or eBioscience	Rat IgG2b
ICOS	APC, PE, PE/Cy7, BV421	C398.4A	Biolegend or eBioscience	Hamster IgG
IL5	APC	TRFK5	Biolegend	Rat IgG1
IL13	PE	eBio13A	eBioscience	Rat IgG1

Ly6C	BV605	HK 1.4	Biolegend	Rat IgG2c
Ly6G	PE, PE/Cy7	1A8	Biolegend	Rat IgG2a
MHCII	FITC	2G9	BD Biosciences	Rat IgG2a
MHCII	Pacific Blue, BV510	M5/114.15.2	Biolegend	Rat IgG2b
NK1.1	eFluor450	PK136	eBioscience	Rat IgG2a
RORγt	PE	AFKJS-9	eBioscience	Rat IgG2a
Siglec-F	APC, AF647	E50-2440	BD Biosciences	Rat IgG2a
Sca-1	APC	D7	Biolegend	Rat IgG2a
ST2	Biotin, PerCp/Cy5.5, FITC	DJ8	MD Bioproducts	Rat IgG1
ST2	eFluor710	RMST2-2	eBioscience	Rat IgG2a
Ter-119	BV421, PE/Cy7	Ter-119	Biolegend	Rat IgG2b
Thy1.2	eFluor450	53-2.1	eBioscience	Rat IgG2a

Table S2. Gating strategy for lung cells in the study. All cells were gated on viable CD45+ cells. Related to Figure 1-4 and 6-7.

**Cell population**	**Gating strategy**
B cells	CD19+ MHCII+
CD4+ T cells	CD3+/CD4+ CD8-
CD8+ T cells	CD3+/CD4- CD8+
T regs	CD3+ CD4+ /CD25+ Foxp3+
ILC2	Lin-(CD3-CD4-CD5-CD8-CD19-CD49b-FceRIa-CD11b-Cd11c-F4/80-Gr1-) Thy1.2+ICOS+ST2+CD25+
NK cells	CD3- /NK1.1+ CD49b+
NKT cells	CD3+ /NK1.1+CD49b+
DCs	CD8-/F4/80-CD11c^{hi} /CD11b^{lo} MHCII^{hi}
AMs	F4/80+ CD11c+ /CD11b^{lo} SiglecF+
PMN	CD19-F4/80- Ly6G+
Eosinophils	CD19-F4/80+ CD11b+ CD11c- SiglecF+ SSC^{hi}
Mast cells	CD19- CD11b+ cKit+ FceRIa+

Table S3. List of primers used in the study. Related to Figure 1, 3-5 and 7.

**Gene target**	**NM name**	**Fragment size**	**Sequence**
mArg1	NM_007482	158bp	F: CAGTGTGGTGCTGGGTGGAG<br>R: ACACAGGTTGCCCATGCAGA
mYm1	NM_009892.1	191bp	F: TCTGGGTACAAGATCCCTGAACTG<br>R: GCTGCTCCATGGTCCTTCCA

mMrc1	NM_008625	116bp	F: TCTGGGCCATGAGGCTTCTC<br>R: CACGCAGCGCTTGTGATCTT
mFizz1	NM_020509	197bp	F: TCCAGCTGATGGTCCCAGTG<br>R: AAAGCCACAAGCACACCCAGT
mIL33	NM_001164724.1	174bp	F: CCCTGGTCCCGCCTTGCAAAA<br>R: AGTTCTCTTCATGCTTGGTACCCGA
mCXCL1	NM_008176	235bp	F: GACCATGGCTGGGATTCACC<br>R: TCAGAAGCCAGCGTTCACCA
mTNF	NM_013693	200bp	F: GAACTGGCAGAAGAGGCACT<br>R: GGTCTGGGCCATAGAACTGA
mHPRT	NM_013556	96bp	F: GTTAAGCAGTACAGCCCCAAAATG<br>R: AAATCCAACAAAGTCTGGCCTGTA

Supplemental Experimental Procedures:

Vacuum-induced lung stress. Lungs were harvested, stored on ice and transferred to either a pre-heated 37°C humidified control chamber or a pre-heated humidified vacuum chamber (Vacuubrand model MZ2C) (see Fig S1A). The vacuum was initiated and both groups were incubated for 6hs. Lungs were then dissociated using scissors and digested in collagenase 1 (750U/ml Invitrogen) and DNase1 (0.31mg/ml Roche) in RPMI (Life technologies) for 45 mins at 37°C. Tissues were passed through a 70µM filter using PBS with 2% FCS and processed for flow cytometry.

Lung flow cytometry and cell sorting. Lung single cell suspensions were prepared by incubating finely minced lung tissue for 1h at 37°C in RPMI containing 5% FCS, collagenase I (ThermoFischer), and DNase I (Sigma-Aldrich), homogenized with a glass homogenizer and then passed through a 70µm strainer. Cells were incubated for 5 min on ice in red blood cell lysis buffer (Sigma-Aldrich), washed and finally passed through a 40µm cell strainer. Single cell suspensions were counted with a hemocytometer and 2x10^{6} cells/stain were incubated with anti-mouse Fc receptor blocking antibody CD16/CD32 (eBioscience) and stained with a mix of fluorochrome labeled antibodies (**Supplementary Methods Table 1**). The alpha-GalCer/CD1d loaded dimer and unloaded control were provided by the NIH tetramer core facility. DAPI or fixable viability dye (eBioscience 65-0865) was added to the surface antibody mix to allow dead cells exclusion by flow cytometry. For gating strategies see **Supplementary Methods Table 2**. For intra-cellular (i.c.) staining, lung cell suspensions were further purified using Percoll (Sigma), incubated with PMA (60ng/ml), ionomycin (500ng/ml) and 1x protein transport inhibitor (eBioscience), or with brefeldin A (GolgiPlug™, BD Biosciences) for 4h at 37°C. Cells were then stained with surface antibodies, fixed and permeabilized before addition of anti mouse IL-13 mAb or anti-IL-5 mAbs, respectively, or isotype control Abs (Fixation and Permeabilization Solution Kit, BD Biosciences). For nuclear staining, cells were permeabilized and processed using the Foxp3 / Transcription Factor Staining Buffer Set (eBioscience). Acquisition was performed with a LSRFortessa (BD Biosciences) and data were analyzed using the FlowJo software version vX.0.7 (TreeStar). Cell sorting was performed with BD FACSAria™ III.

Reagents for isolation, culture and stimulation of alveolar macrophages (AMs). AMs from newborn mice were isolated by cell sorting from lung single cell suspensions as described above. In adult mice, AMs were isolated by bronchoalveolar lavage (BAL) followed by cell adhesion. In brief, mice were lethally anesthetized using 100 mg/kg body weight ketamine (Ketaset) and 10 mg/kg body weight xylazine (Rompun, Bayer), administered in sterile PBS i.p. AMs were isolated by inserting a tracheal cannula (Venflon, BD Bioscience) and flushing the lungs 10 times with 1ml NaCl. Isolated cells were counted and allowed to adhere at 37°C for 2h in RPMI containing 10% FCS and 1% penicillin/streptomycin at a concentration of 5x10^{4} cells/well in a 96 well plate. Wells were washed twice with PBS and adherent cells were used for further experiments.

Real time PCR. Total mRNA was isolated using the NucleoSpin RNA XS kit (Macherey-Nagel) or the RNeasy Micro kit (Qiagen) according to the manufacturers' instructions. Real-time PCR was performed using either the SYBR Green Master Mix (Applied Biosystems), or the TaqMan universal PCR mix (Applied Biosystems) and the StepOnePlus™ Real-Time PCR System (Applied Biosystems) or using a ViiA *7 (*Thermofisher*).* Commercially available Taqman probes were used for the expression of mCCL17 (Mm01244826_g1), mYm1 (Mm00657889_mH), mFizz1 (Mm00445109_m1), mArg1 (Mm00475988_m1) and GAPDH (Cat. 4352932E). Designed primers were purchased from Sigma-Aldrich and are listed in the **Supplementary Methods Table 3.** Gene expression was normalized to GAPDH or HPRT and expressed as fold change versus indicated controls.

Lung tissue homogenates and ELISA. Lungs were homogenized in the presence of protease inhibitors and total protein content was quantified by Pierce BCA Protein Assay Kit (Thermo Scientific). The Multiplex bead array (MILLIPLEX MAP Mouse Cytokine/Chemokine Magnetic Bead Panel - Premixed 25 Plex) was performed according to the manufacturer's instructions (Millipore), using MAGPIX multiplexing instrument and MILLIPLEX analysis 5.1 software (Millipore). Mouse IL-33 was measured using the eBioscience ELISA kit, according to the manufacturers' instructions. For protein quantification in *S. pneumoniae* infected lungs, CXCL1 and TNF were measured in lung homogenates using specific ELISA kits from RnD Systems, according to the manufacturers' instructions.

Analysis of alveolarization. H&E stained lung sections of newborn mice at postnatal day 7 were imaged using an Olympus FSX100 automated microscope and a 20X magnification (10 fields per lung). Lung alveolarization was quantified using the automatic image analysis software CellProfiler (Lamprecht et al., 2007) (http://www.cellprofiler.org/). Briefly, image masks for each field were generated by converting the images to grayscale and applying a binary threshold using the threshold function in ImageJ (version 1.49v; http://imagej.nih.gov/ij/). The original images were loaded into CellProfiler and alveoli were identified using the binary images generated in ImageJ (as described above), as the thresholding method using the *IdentifyPrimaryObjects* module. The shape and size features of the identified alveoli were then calculated using the *MeasureObjectSizeShape* module and exported to a spreadsheet format.

Immunofluorescence and analysis. Lungs from *Il33*$^{Cit/+}$ reporter mice were isolated at embryonic day 19 (E19) and postnatal days 1 (P1) and 3 and fixed overnight in 1% formaldehyde–PBS at 4 °C. After extensive washing with PBS, lungs were then incubated overnight in 30% sucrose solution at 4 °C. On the third day, lungs were embedded in 15% sucrose + 7.5% porcine skin gelatin (Sigma) in PBS and flash-frozen in isopentane chilled to −80 °C with liquid nitrogen. Lobes were sectioned to a 20-µm thickness onto Superfrost Plus slides (Thermo Scientific) and stored at −20 °C. Sections were air-dried for 1 h, rehydrated with PBS and then incubated in blocking buffer (3% goat serum (Jackson Immunoresearch) + 0.05% Triton-X in PBS) for 30 min. Sections were then incubated with a polyclonal rabbit anti-pro-surfactant protein-C antibody (Merck Millipore, AB3786) (1:500) in blocking buffer for 1 h. After washing, sections were then incubated with an Alexa Fluor 546-labeled polyclonal goat anti-rabbit antibody (4 µg/ml) (Thermo Fisher Scientific) and DAPI (300 nM) for 30 min in blocking buffer. After additional washes, Prolong Gold (Invitrogen) was added to slides plus a coverslip. Sections were imaged on a laser-scanning microscope (TCS SP8, Leica) with a 20×/ 20x/0.75NA HC PL APO CS2 air objective. Data were processed and analyzed using the open-source software ImageJ (Fiji package) (Schindelin et al., 2012).

Supplemental References:

Lamprecht, M.R., Sabatini, D.M., Carpenter, A.E., 2007. CellProfiler: Free, versatile software for automated biological image analysis. Biotechniques 42, 71–75. doi:10.2144/000112257

Schindelin, J., Arganda-Carreras, I., Frise, E., Kaynig, V., Longair, M., Pietzsch, T., Preibisch, S., Rueden, C., Saalfeld, S., Schmid, B., Tinevez, J.-Y., White, D.J., Hartenstein, V., Eliceiri, K., Tomancak, P., Cardona, A., 2012. Fiji: an open-source platform for biological-image analysis. Nat. Methods 9, 676–682. doi:10.1038/nmeth.2019
