# Supplementary material for: First-Breath-Induced Type 2 Pathways Shape the Lung Immune Environment
Source: Cell Rep. 2017 Feb 21;18(8):1893–905. doi: 10.1016/j.celrep.2017.01.071 (PMC5329122; doi:10.1016/j.celrep.2017.01.071)
Supplement: Document S2. Article plus Supplemental Information [file mmc2.pdf]

Cell Reports

Article

First-Breath-Induced Type 2 Pathways Shape the Lung Immune Environment

Graphical Abstract

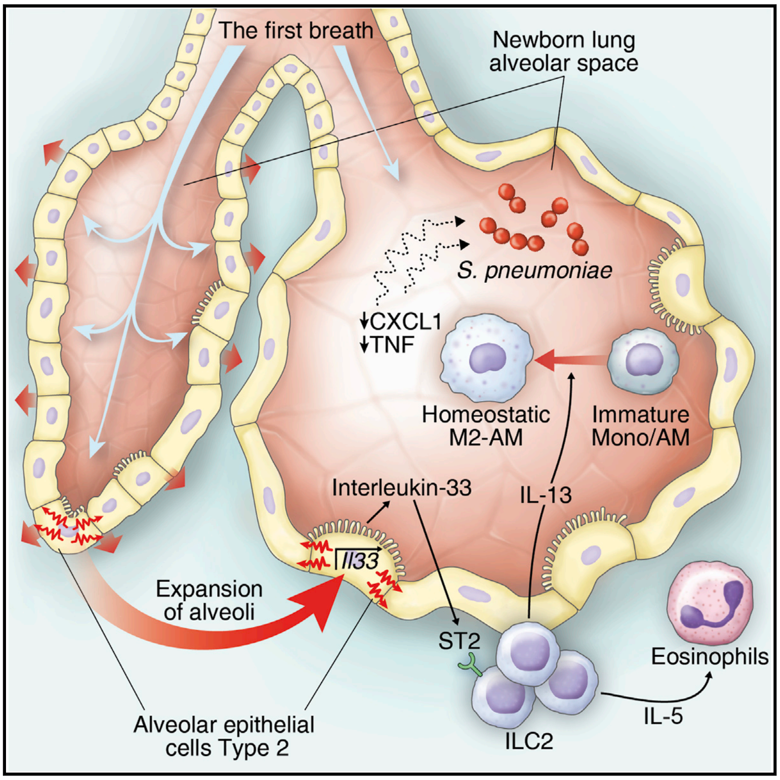

Highlights

- The first breath triggers IL-33 induction by AEC2 in lungs of newborn mice
- IL-33 promotes the perinatal expansion and activation of ST2-expressing ILC2s
- ILC2-derived IL-13 polarizes newborn's AMs into an M2 phenotype
- This homeostatic type 2 pathway delays antibacterial effector responses

Authors

Simona Saluzzo, Anna-Dorothea Gorki, Batika M.J. Rana, ..., Ildiko Mesteri, Andrew N.J. McKenzie, Sylvia Knapp

Correspondence

anm@mrc-lmb.cam.ac.uk (A.N.J.M.),
sylvia.knapp@meduniwien.ac.at (S.K.)

In Brief

The postnatal lung immune environment is largely unexplored. Saluzzo et al. find that birth is associated with the induction of an IL-33/ILC2/IL-13-driven immune response in mice. This type 2 response is preserved throughout life and maintains alveolar macrophages in an M2 phenotype at the expense of a delayed response to bacterial infections.

Saluzzo et al., 2017, Cell Reports *18*, 1893–1905
February 21, 2017 © 2017 The Authors.
http://dx.doi.org/10.1016/j.celrep.2017.01.071

Cell Reports
Article

OPEN ACCESS
CellPress

First-Breath-Induced Type 2 Pathways Shape the Lung Immune Environment

Simona Saluzzo,[1,2,3] Anna-Dorothea Gorki,[1,2] Batika M.J. Rana,[3] Rui Martins,[1,2] Seth Scanlon,[3] Philipp Starkl,[1,2] Karin Lakovits,[1,2] Anastasiya Hladik,[1,2] Ana Korosec,[1,2] Omar Sharif,[1,2] Joanna M. Warszawska,[1,2] Helen Jolin,[3] Ildiko Mesteri,[4] Andrew N.J. McKenzie,[3,*] and Sylvia Knapp[1,2,5,*]

[1]CeMM Research Center for Molecular Medicine of the Austrian Academy of Sciences, Vienna 1090, Austria
[2]Department of Medicine I, Laboratory of Infection Biology, Medical University of Vienna, Vienna 1090, Austria
[3]MRC Laboratory of Molecular Biology, Francis Crick Avenue, Cambridge CB2 0QH, UK
[4]Institute of Pathology Überlingen, Überlingen 88662, Germany
[5]Lead Contact
*Correspondence: anm@mrc-lmb.cam.ac.uk (A.N.J.M.), sylvia.knapp@meduniwien.ac.at (S.K.)
http://dx.doi.org/10.1016/j.celrep.2017.01.071

SUMMARY

From birth onward, the lungs are exposed to the external environment and therefore harbor a complex immunological milieu to protect this organ from damage and infection. We investigated the homeostatic role of the epithelium-derived alarmin interleukin-33 (IL-33) in newborn mice and discovered the immediate upregulation of IL-33 from the first day of life, closely followed by a wave of IL-13-producing type 2 innate lymphoid cells (ILC2s), which coincided with the appearance of alveolar macrophages (AMs) and their early polarization to an IL-13-dependent anti-inflammatory M2 phenotype. ILC2s contributed to lung quiescence in homeostasis by polarizing tissue resident AMs and induced an M2 phenotype in transplanted macrophage progenitors. ILC2s continued to maintain the M2 AM phenotype during adult life at the cost of a delayed response to *Streptococcus pneumoniae* infection in mice. These data highlight the homeostatic role of ILC2s in setting the activation threshold in the lung and underline their implications in anti-bacterial defenses.

INTRODUCTION

The integrity of the alveolar-capillary barrier is essential to ensure sufficient blood oxygen levels, and the mechanisms driving its maintenance, renewal, and protection are flourishing fields of research (Beers and Morrisey, 2011; Chiu and Openshaw, 2015; Hogan et al., 2014; Hussell and Bell, 2014; Kopf et al., 2015; Peng et al., 2015). Lung development begins at embryonic day 9 (E9) in mice and proceeds through stages of branching morphogenesis, giving rise to pre-alveolar spaces at the saccular stage and the differentiation of type 1 and type 2 airway epithelial cells (AEC1s and AEC2s) by E18.5 (Mund et al., 2008; Woik and Kroll, 2015). At birth, alveolar sacs are suddenly exposed to the external environment and subjected to the mechanical forces of spontaneous ventilation (Orr et al., 2006; Wirtz and Dobbs, 2000). It is after the previously sterile lung tissue has been exposed to the outside environment, around postnatal day 4 (P4), when the process of alveologenesis continues with the formation of primary septa (Hogan et al., 2014). These postnatal adaptations are paralleled by the development of the early innate immune environment. Alveolar macrophages (AMs) differentiate on P3 from $CD11b^{hi}F4/80^{int}Ly6C^{hi}$ fetal monocyte progenitors into long-lived, self-renewing cells (Guilliams et al., 2013; Murphy et al., 2008). Since tissue-derived signals were found to govern the gene expression signature of macrophages (Lavin et al., 2014; Okabe and Medzhitov, 2014), the lung cytokine milieu in newborns likely determines the phenotype of AMs during this delicate developmental period. However, the postnatal immunological environment in lungs is largely unexplored, as are the innate immune signals that influence the function of AMs early in development and during homeostasis.

Under different pathological conditions, AMs have the ability to assume either an interferon-γ (IFN-γ) and Toll-like receptor (TLR) ligand-induced inflammatory phenotype (M1) or an interleukin-4 (IL-4)-, IL-13-, or IL-10-induced wound healing and tissue remodeling phenotype (M2) (Gordon and Martinez, 2010). M1 macrophages are potent producers of inflammatory cytokines such as tumor necrosis factor (TNF) and CXCL1 (Guery et al., 2011; Mantovani et al., 2004). CXCL1 is a chemokine that critically determines the early recruitment of neutrophils (De Filippo et al., 2013), thereby exerting a protective role in bacterial lung infections (Schliehe et al., 2015; Warszawska et al., 2013). M2 macrophages, phenotypically defined by the expression of *Retnla* (referred to here as *Fizz1*), *Mrc1*, *Chil3* (referred to here as *Ym1*), and *Arg1* are less efficient in triggering inflammatory responses to bacterial pathogens than M1 macrophages (Warszawska et al., 2013).

IL-33 is an alarmin belonging to the IL-1 family of cytokines best known for its capacity to drive type 2 immune responses (Liew et al., 2010; Schmitz et al., 2005) and is increasingly recognized as an important mediator of homeostasis and tissue tolerance (Molofsky et al., 2015). Upon mechanical strain or cell necrosis (Kakkar et al., 2012; Lamkanfi and Dixit, 2009; Sanada et al., 2007), IL-33 is released from cells, activating the ST2 receptor expressed on several lung cell types, including regulatory T cells (T_{reg}), dendritic cells (DCs), mast cells, group 2 innate

CrossMark

This is an open access article under the CC BY license (http://creativecommons.org/licenses/by/4.0/).

lymphoid cells (ILC2s), natural killer (NK) cells, and AMs (Lu et al., 2015). At steady state, lung resident ILC2s are the most abundant ST2-expressing cells and are found in close proximity to bronchovascular structures (Halim et al., 2014; Nussbaum et al., 2013), where they can be rapidly activated by IL-33 to secrete IL-13, IL-5, IL-6, IL-9, granulocyte-macrophage colony-stimulating factor (GM-CSF), and amphiregulin (Roediger and Weninger, 2015). ILC2s are involved in host protection against parasitic helminths and promotion of airway hyperreactivity in asthma or upon influenza infection and are important for adipose tissue homeostasis (Barlow et al., 2012; Brestoff et al., 2015; Chang et al., 2011; Molofsky et al., 2013; Monticelli et al., 2011; Neill et al., 2010).

Human lungs are highly susceptible to bacterial infections. Pneumonia caused by *Streptococcus pneumoniae* is the primary cause of death by an infectious disease in Western countries (van der Poll and Opal, 2009). Notably, risk factors for developing community-acquired pneumonia are asthma and influenza (Chien et al., 2009; Talbot et al., 2005), which are both characterized by IL-13-induced airway hyperreactivity (Kim et al., 2012; Lambrecht and Hammad, 2015) and the presence of M2 polarized AMs (Chen et al., 2012). Here, we investigated the physiological role of the IL-33/ILC2/IL-13 axis in shaping the pulmonary immune environment from birth to adult life and the consequences of these pathways on the innate defense against *S. pneumoniae*.

RESULTS

Postnatal Lung Inflation Is Associated with the Upregulation of IL-33 by AEC2

With the first breath, a number of profound changes occur in the newborn's lung. We hypothesized that the sudden inflation of the previously liquid-filled lungs may cause considerable mechanical stress and potential tissue injury, which could result in IL-33 induction (Kakkar et al., 2012). We discovered a substantial increment in pulmonary IL-33 on P1 compared to E19 at both the protein (Figure 1A) and mRNA levels (Figure 1B). To investigate if an abrupt exposure to negative pressure, occurring upon spontaneous breathing in the alveolar space, might cause the induction of *Il33*, we placed the lungs of E19 *Il33*$^{Cit/+}$ reporter (Hardman et al., 2013) and WT mice in a vacuum chamber (Figure S1A) and discovered a significant induction of Citrine^{+} viable cells (Figures 1C, 1D, S1B, and S1C) and IL-33 protein (Figure 1E) in lungs 6 hr post-exposure to negative pressure as compared to ambient atmospheric pressure.

To study the cellular origin of pulmonary IL-33 over time, we analyzed lungs of *Il33*$^{Cit/+}$ reporter mice by flow cytometry. We observed a strong upregulation of *Il33* among the CD45^{-} cell fraction starting on P1 (Figures 1F, 1G, S1D, and S1E). Approximately 60% of CD45^{-} citrine^{+} cells were further classified as EpCam^{+}CD31^{-} cells (Figures 1H and 1I). Immunohistochemistry revealed that AEC2 (surfactant protein C^{+}) was the most abundant cell population upregulating *Il33* in the first few days after birth (Figure 1J). Postnatally infiltrating CD45^{+} cells (Figure 1F, top, and Figure S1F) did not show substantial *Il33* expression (Figure S1G), except for few citrine^{+} cells in the fetal macrophage fraction (CD45^{+}F4/80^{+}CD11b^{+}CD11c^{-} SiglecF^{-}) (Figures S1H and S1I). In summary, we determined that postnatal lung inflation or exposure to abrupt changes in pressure was associated with the immediate induction of IL-33.

IL-33 Shapes the Neonatal Lung Environment

To understand IL-33-dependent effects on the immune environment in neonatal lungs, we first analyzed a panel of pulmonary cytokines and chemokines at P7 in wild-type (WT) and IL-33-deficient (*Il33*$^{Cit/Cit}$) mice. *Il33*$^{Cit/Cit}$ mice showed reduced expression of the type 2 cytokines IL-5 and IL-9 and of inflammatory mediators like IL-6, IFN-γ, IL-1α, IL-1β, CCL5, and CXCL10 (Figures 2A and S2A). Since ST2^{+} ILC2s are major producers of IL-5 and Il-9 and considered the primary targets of IL-33 in the lung (Halim et al., 2014; Kearley et al., 2015), we analyzed newborn lungs for the presence of ILC2s (Lin^{-} CD127^{+}ST2^{+}ICOS^{+}). We detected few ILC2s at E19 but markedly increased numbers by P7 that stabilized by week 6 (Figures 2B and S2B). We found IL-33 to be contributory in populating lungs with ILC2s, as illustrated by reduced ILC2 numbers in *Il33*$^{Cit/Cit}$ (Figure 2C) and ST2-deficient (*Il1rl1*$^{-/-}$) mice at P7 (Figure 2D). In accordance with the ability of ILC2s to regulate eosinophil homeostasis via IL-5 secretion (Nussbaum et al., 2013), eosinophils populated the lungs a few days after ILC2s (Figures 2E and S2C), with clear reductions in the absence of IL-33 (Figure 2F) or ST2 (Figure 2G). IL-5+ ILC2s expanded locally in the lungs (Figures S2E–S2G), whereas eosinophil numbers increased systemically (Figure 2H). The numbers of AMs, polymorphonuclear (PMNs) cells, B cells, and T cells were not changed in *Il33*$^{Cit/Cit}$ mice (Figures 2F, 2G, and S2D). Importantly, the postnatal alveolarization process (Hogan et al., 2014) was not affected by the absence of IL-33 (Figure S2H).

Collectively, these data indicate a critical role for IL-33 in shaping the immune cell infiltrate in the neonatal lung by promoting the appearance of ILC2s and eosinophils. This early period, in which the lung immunological environment is being established, may have subsequent effects on adult lung homeostasis and host defense.

AM Development in Neonatal Lungs Coincides with ILC2 Activation

We next examined the activation state of postnatally expanded ILC2s in lungs using *Il13* tdTomato (*Il13*$^{Tom/+}$) reporter mice (Barlow et al., 2012). IL-13-expressing ILC2s began to expand at P3, peaked at 70% on P10, and started to decline by P14 (Figures 3A, 3B, and S3A–S3C). Perinatal IL-13 expression was restricted to Lin^{-} cells (Figure S3D) and depended on the presence of IL-33 (Figure S3E). The expansion of activated ILC2s coincided with the emergence of AMs (Figures 3C, 3D, and S3F).

Considering the critical role of IL-13 in driving the alternative activation of macrophages, we tested to what degree this postnatal wave of ILC2-derived IL-13 might contribute to the immediate polarization of newly differentiated AMs (Gordon and Martinez, 2010). We discovered reduced expression levels of the M2 markers *Ym1*, *Arg1*, and *Fizz1* in *Il13*$^{-/-}$ and *Il1rl1*$^{-/-}$ AMs (Figures 3E and S3G). Further, we found elevated spontaneous expression levels of *Cxcl1* and *Tnf* in *Il13*$^{-/-}$ as compared to WT AMs on P7 (Figures 3E and 3F). Remarkably, the amount of CXCL1 released by WT AMs declined with age, whereas

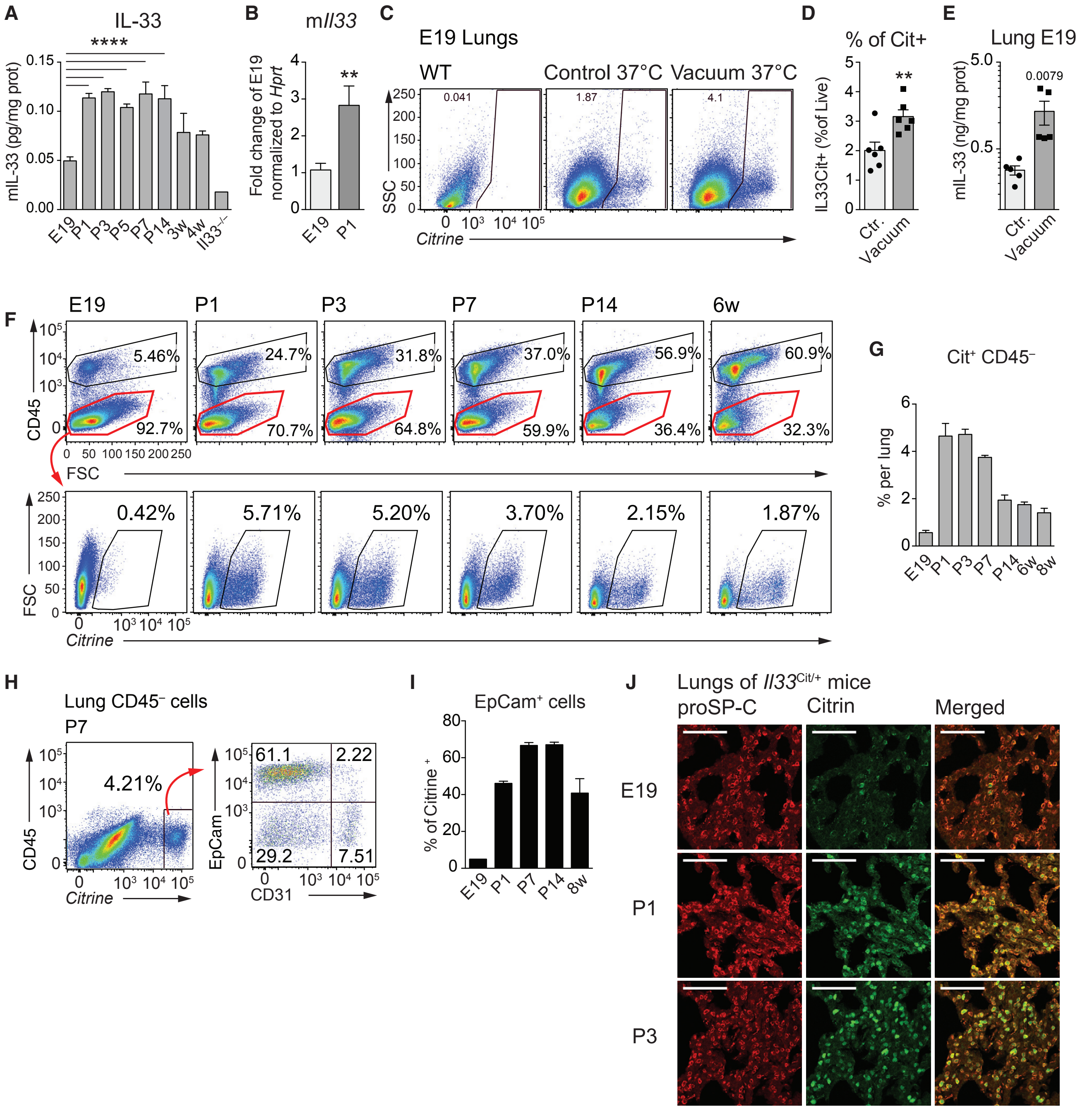

Figure 1. Type 2 Alveolar Epithelial Cells Induce IL-33 at Birth

(A) Whole-lung IL-33 quantification by ELISA at E19; postnatal days 1, 3, 5, 7, and 14 (P1–P14); and 3 and 4 weeks (3–4w) after birth.

(B) qRT-PCR of pulmonary *Il33* expression in WT mice at E19 and P1.

(C) FACS analysis of viable citrine$^+$ cells from *Il33*$^{Cit/+}$ mice at E19 exposed to vacuum or atmospheric pressure (control) for 6 hr.

(D) Quantification of (C).

(E) Whole-lung IL-33 quantification by ELISA of WT lungs at E19 exposed to vacuum or atmospheric pressure (control) for 6 hr.

(F) FACS analysis of lung CD45 and citrine expression in *Il33*$^{Cit/+}$ reporter mice at the indicated time points (gates are set using WT as controls).

(G) Percentage of Cit$^+$CD45$^-$ cells among lung cells, gated as in (F).

(H) Flow cytometry of viable CD45$^-$ lung cells from *Il33*$^{Cit/+}$ mice at P7, stained for EpCam and CD31.

(I) Quantification of the *Cit*$^+$ proportion of EpCAM$^+$ cells between E19 and 8 weeks of age.

(J) Micrographs of lung sections at E19, P1, and P3 from *Il33*$^{Cit/+}$ reporter mice. Red, surfactant protein C (SP-C); green, IL-33-driven citrine. Scale bars represent 75 μm.

Data are representative of two independent experiments with three to five mice per time point, and graph bars represent mean ± SEM. **$p < 0.01$ and ****$p < 0.0001$.

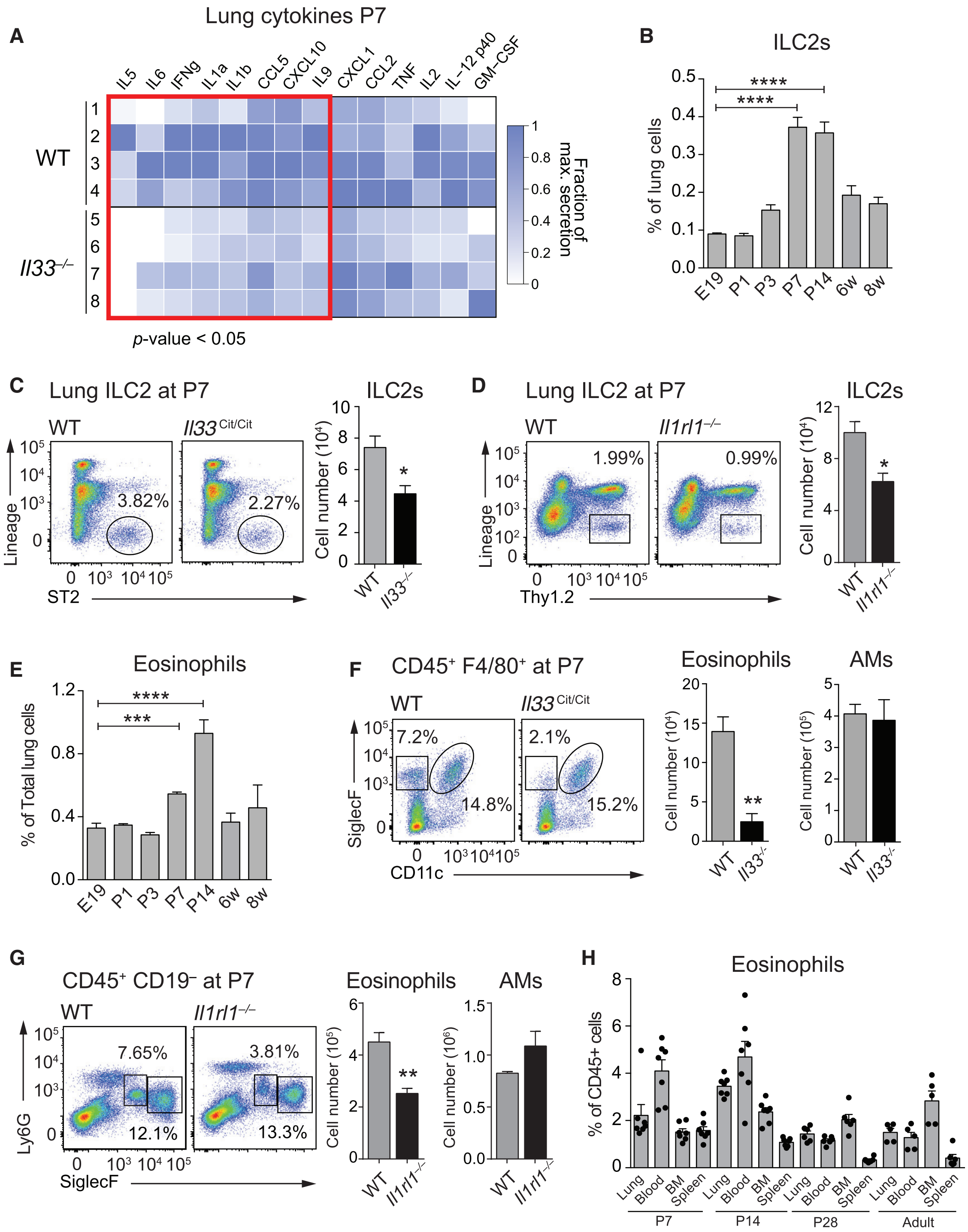

Figure 2. IL-33 Drives a Type 2 Immune Environment in Lungs of Newborns

(A) Heatmap representation of cytokine levels in whole lung homogenates comparing WT and *Il33*$^{Cit/Cit}$ mice at P7. Original values (see Figure S2A) were rescaled between zero and the maximum value detected for each cytokine and are presented as the fraction of maximum secretion.

(B) Percentage of lung ILC2s (Lin$^-$ ST2$^+$ Thy1.2$^+$ CD25$^+$ ICOS$^+$) analyzed by FACS at the indicated time points.

(C) FACS analysis of lung ILC2s (Lin$^-$ ST2$^+$) in WT and *Il33*$^{Cit/Cit}$ mice at P7, further gated for CD25$^+$ and ICOS$^+$ and quantified (right).

(D) FACS analysis of lung ILC2s (Lin$^-$ Thy1.2$^+$) in WT and *Il1rl1*$^{-/-}$ mice at P7, further gated for CD25$^+$ and ICOS$^+$ and quantified (right).

(E) Percentage of lung eosinophils (F4/80$^+$ CD11b$^+$SiglecF$^+$CD11c$^-$) analyzed by FACS at the indicated time points.

(F and G) FACS analysis of lung eosinophils (CD11b$^+$SiglecF$^+$CD11c$^-$) and AMs (CD11b$^-$SiglecF$^+$CD11c$^+$) at P7 in WT and *Il33*$^{Cit/Cit}$ mice (F) and WT and *Il1rl1*$^{-/-}$ mice (G).

(legend continued on next page)

AMs from *Il13*$^{-/-}$ mice continued to produce high levels of CXCL1 until P21 (Figure 3G). These data demonstrate that postnatal AMs exhibit an M1 phenotype and that IL-13 promotes the deactivation and M2 polarization of AMs over time.

IL-13 Maintains Adult Resident AMs in an M2 State

AMs are long-lived cells with local self-renewal capacity, which are, like other macrophages, strongly influenced by the environment they inhabit (Guilliams et al., 2013; Lavin et al., 2014; Murphy et al., 2008). We reasoned that the need for an unremittingly quiescent lung environment throughout life would favor an M2 AM phenotype and discovered that pulmonary IL-13 continued to affect the M2 polarization of AMs in adult mice (Figure 4A). Adult *Il13*$^{-/-}$ AMs stimulated with the respiratory pathogen *S. pneumoniae* or the TLR2 ligand lipoteichoic acid (LTA) consistently induced higher levels of CXCL1 than WT AMs (Figures 4B, 4C, and S4A). WT monocytes adoptively transferred to the lungs of WT or *Il13*$^{-/-}$ mice differentiated toward a SiglecF^{+} AM phenotype within 2 weeks (Figure S4B) and upregulated M2 markers in WT, but not *Il13*$^{-/-}$, recipient animals (Figures 4D and S4C). These results confirm that a tissue-derived source of IL-13 is required to polarize and maintain AMs in an M2 state in adult mice. Of note, IL-33 itself was not sufficient to shape the polarization and activity of AMs from adult mice, as *Il1rl*$^{-/-}$ AMs did not differ from WT AMs in their expression of M2 markers or response to *S. pneumoniae* (Figures S4D–S4F). In summary, endogenous IL-13 contributes to the M2 phenotype of resident and monocyte-derived AMs in healthy adult mice and is required to suppress potentially excessive inflammation.

Pulmonary IL-13 Is Detrimental upon Pneumococcal Infection

We hypothesized that the IL-13-driven M2 polarization of AMs might impact on innate defenses against *S. pneumoniae*. Upon infection of *Il13*$^{-/-}$ and WT mice with *S. pneumoniae*, we observed a more pronounced early (6 hr) influx of neutrophils in bronchoalveolar lavage fluid (BALF) and lung (Figures 4E and 4F) and enhanced amounts of lung CXCL1 (Figure 4G) in *Il13*$^{-/-}$ mice. This augmented early inflammatory response in the absence of *Il13* translated into an improved bacterial clearance from lungs 48 hr post-infection and completely prevented the systemic spread of bacteria (Figures 4H and 4I). In accordance with the reduced bacterial burden, we found decreased CXCL1 levels (Figure 4J), lower numbers of infiltrating monocytes (Figure S5A), and less pronounced lung infiltrates at 48 hr post-infection in *Il13*$^{-/-}$ as compared to WT animals (Figure 4K). To assess the broader relevance of these findings, we investigated the contribution of pulmonary IL-13 to host defense against *Staphylococcus aureus* as well as upon induction of lipopolysaccharide (LPS)-induced acute lung injury. Similar to our observations in pneumococcal pneumonia, we discovered an augmented early inflammatory response to LPS (Figures S5B and S5C) and an improved clearance of *S. aureus* associated with a reduced disease-associated temperature drop in *Il13*$^{-/-}$ animals (Figures S5D and S5E). Together, these results support the notion that pulmonary IL-13 shapes the immune environment in the lung, which upon infection delays the induction of innate defenses against pathogens.

We could not detect any baseline differences in immune cells involved in the defense against bacteria (NK, T, B, or PMN cells and monocytes; Figure S5F; data not shown) or in ILC2 levels (Figure S5G), except for an increased number of eosinophils in *Il13*$^{-/-}$ mice (Figure S5H). To exclude the possibility that eosinophils contributed to the phenotype, we repeated the infection studies in *Il5*$^{-/-}$ mice, which have severely reduced pulmonary eosinophilia (Figure S5I), and could not identify any differences in bacterial counts (Figure S5J) or the inflammatory response elicited by AMs in vitro (Figure S5K).

A short-term in vivo exposure to rmIL-13 was sufficient to "re-polarize" resident AMs from IL-13-deficient mice toward an M2 phenotype (Figure 5A) and reduce CXCL1 releases induced by *S. pneumoniae* (Figures 5B and 5C). Finally, the intranasal administration of rmIL-13 to *Il13*$^{-/-}$ mice impaired bacterial clearance in lungs and blood, with bacterial counts being comparable to WT controls (Figures 5D and 5E). In summary, these data demonstrate that the lung immune environment at homeostasis is profoundly shaped by IL-13 at the expense of impaired anti-bacterial defenses.

Resident ILC2s Are the Sole Source of IL-13 in Healthy Adult Lungs

To determine the potential contribution of ILC2s to the AM phenotype, we evaluated the activity of lung ILC2s in adult mice using *Il13*$^{Tom/+}$ reporter mice and intracellular cytokine staining. We found that ~7%–8% of ILC2s in the lungs of naive adult mice (~3×10^{3} cells) produced IL-13 (Figures 6A–6C). Notably, we excluded Th2 cells, eosinophils, mast cells, macrophages, NK cells, natural killer T (NKT) cells, and invariant natural killer T (iNKT) cells, which have all been shown to produce IL-13 in different lung pathological conditions (Kim et al., 2008; Price et al., 2010; Rijavec et al., 2011) as the source of IL-13 at steady state in healthy adult lungs (Figures 6D and S6A). In fact, ILC2s were the only cells expressing *Il13* in healthy adult lungs at homeostasis (Figure 6D), a finding we confirmed by intracellular staining for IL-13 (Figure S6B).

Constitutive IL-13 production by lung resident ILC2s did not depend on T or B cells in adult mice (Figures S6C–S6G). However, homeostatic IL-13 production depended on ST2 (Figure 6E) and less so on IL-25, another cytokine capable of inducing IL-13 production by ILC2s via IL17rb (Roediger and Weninger, 2015) (Figure S6H). The absolute number of lung ILC2s did not change in the absence of ST2 or IL17rb (Figure S6I). In summary, lung resident ILC2s are a constant and unique source of pulmonary IL-13 in healthy adult lungs at steady state.

To test if IL-13-producing ILC2s alone were sufficient to determine the responsiveness of AMs to *S. pneumoniae*, we

(H) Lung, blood, bone marrow, and spleen cells were analyzed by FACS for eosinophils (CD11b^{+}SiglecF^{+}F480^{+}CD11c^{-}) in P7, P14, P28, and adult (6–8 weeks) *Il5*$^{Cer/+}$ mice.

Data are representative of one (A and H) or two (B–G) independent experiments with four mice per group. Graph bars represent mean ± SEM. *$p < 0.05$, **$p < 0.01$, ***$p < 0.001$, and ****$p < 0.0001$. For flow cytometry, all cells were pre-gated on viable, single, CD45^{+}. E, embryonic; p, postnatal; w, week.

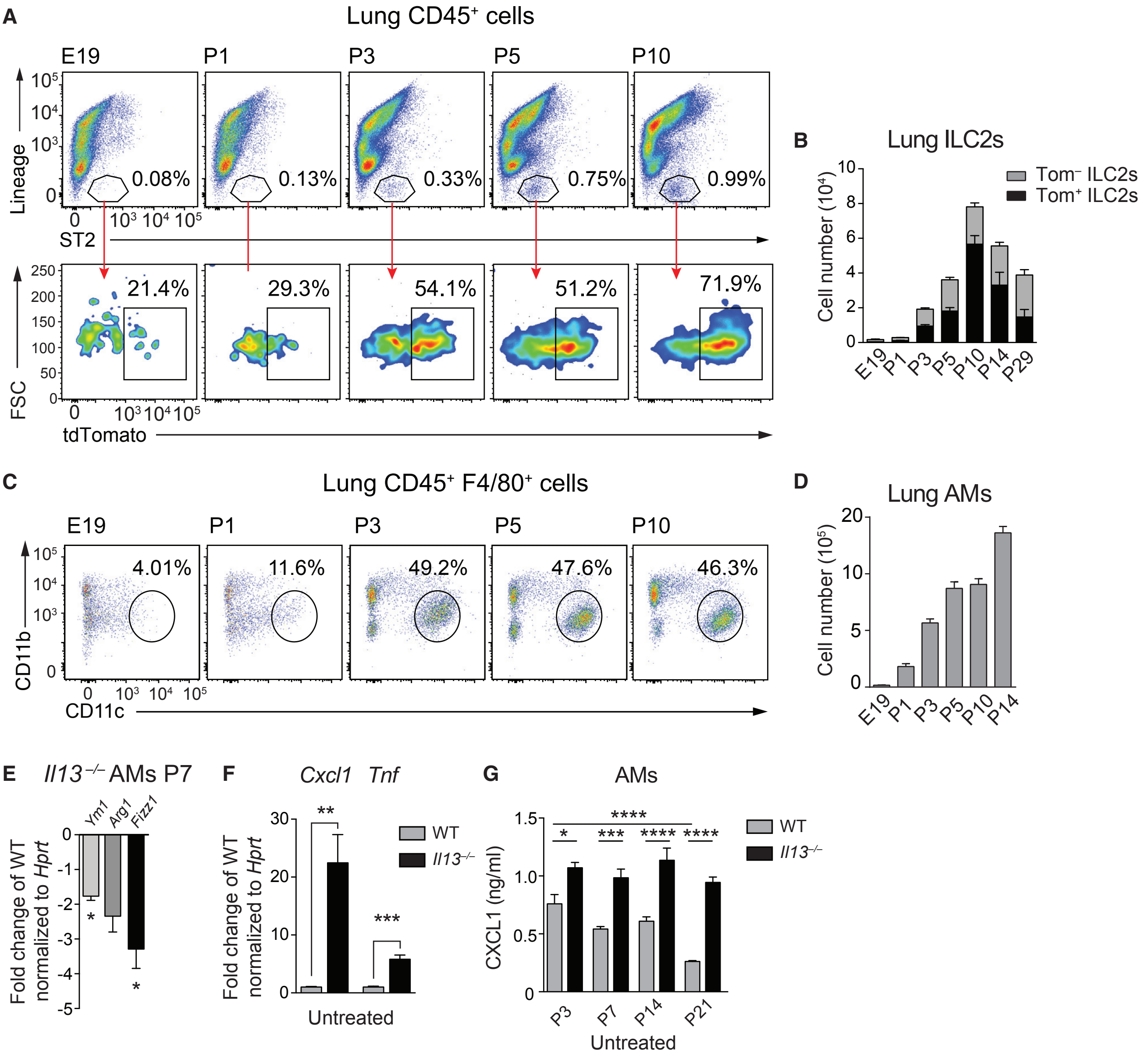

Figure 3. Lung ILC2s Expansion and Activation Coincides with AM Differentiation and M2 Polarization

(A) Representative FACS profiles of expanding lung ILC2s (Lin^- $ST2^+$) (top) and proportion of *Tom*$^+$ cells (bottom) in *Il13*$^{Tom/+}$ mice between E19 and P10.

(B) Quantification of absolute numbers of lung Lin^- $ST2^+$ $Thy1.2^+$ $Tom^{+/-}$ cells at the indicated time points.

(C) FACS plots illustrating percentages of AMs ($F4/80^+CD11b^-CD11c^+$) at the indicated time points.

(D) Absolute numbers of AMs gated as in (C) between E19 and P14.

(E) AMs ($F4/80^+CD11b^-CD11c^+SiglecF^+$) were sorted on P7 from WT and *Il13*$^{Tom/Tom}$ mice and M2 markers were assessed by RT-PCR.

(F) AMs from WT and *Il13*$^{Tom/Tom}$ (IL-13 deficient) mice on P7 were isolated as in (E) and cultured for 6 hr, and *Cxcl1* and *Tnf* gene induction was assessed by RT-PCR. Values were normalized to *Hprt* and are expressed as fold change versus WT.

(G) AMs from WT and *Il13*$^{Tom/Tom}$ (IL-13 deficient) mice on P3, P7, P14, and P21 were isolated as in (E) and cultured for 6 hr, and spontaneous CXCL1 secretion was quantified by ELISA.

Data are representative of three (A–D) or two (E–G) independent experiments with three or four mice per group. Values were normalized to *Hprt* and are expressed as fold change versus the indicated control. Bars represent mean ± SEM; *$p < 0.05$, **$p < 0.01$, ***$p < 0.001$, and ****$p < 0.0001$.

adoptively transferred IL-33-expanded lung Tom$^+$ ILC2s to WT and *Il13*$^{-/-}$ mice (Figure 6F). We observed a significant reduction of *S.-pneumoniae*-induced CXCL1 secretion by AMs isolated from *Il13*$^{-/-}$ recipients that received Tom$^+$ ILC2s (Figure 6G). Of note, adoptively transferred ILC2s were also able to reduce the responsiveness of WT AM to *S. pneumoniae* in vitro.

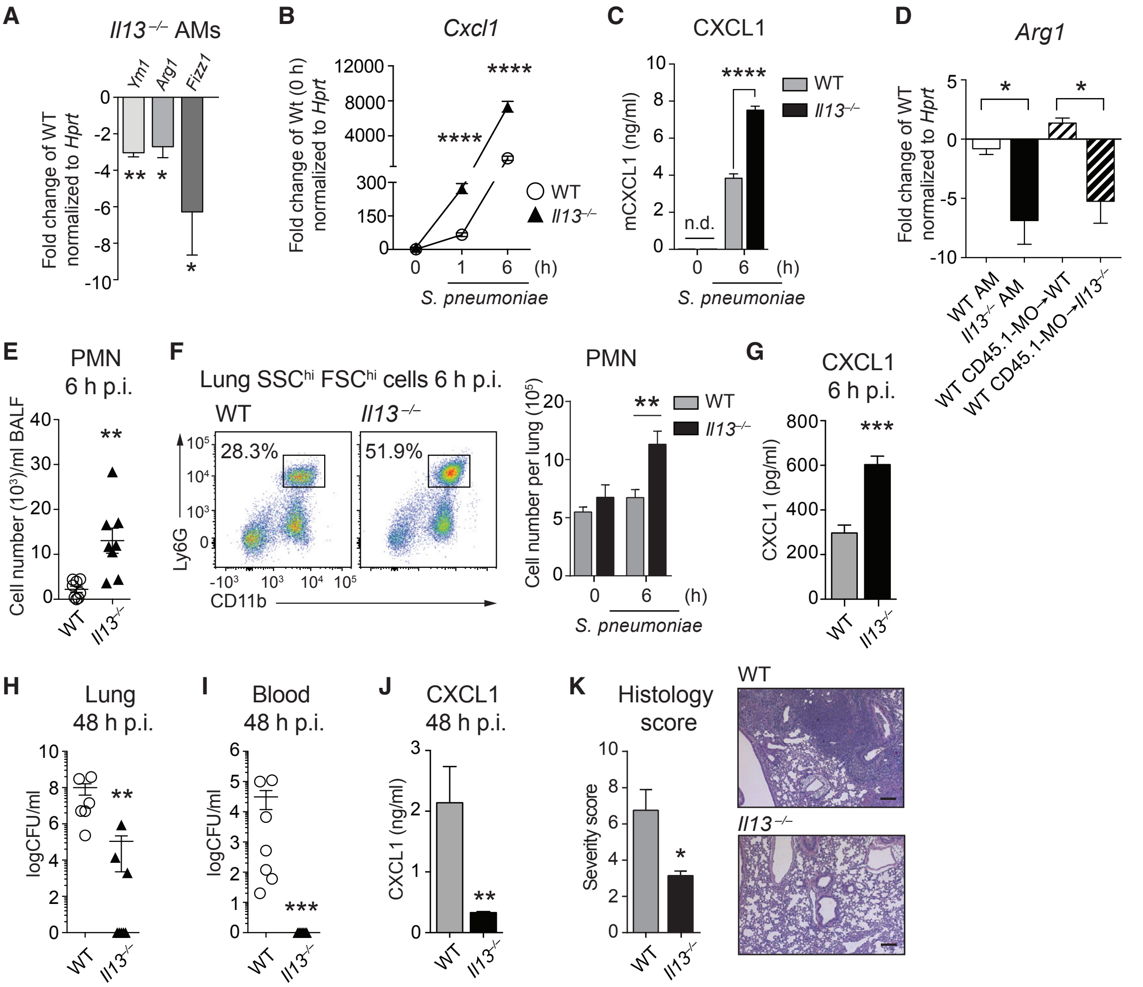

Figure 4. AMs from IL-13-Deficient Mice Present a Pro-inflammatory Phenotype and Improved Defenses against *S. pneumoniae*

(A) AMs from adult WT and *Il13*$^{-/-}$ mice were isolated by bronchoalveolar lavage and analyzed for expression of M2 polarization markers by RT-PCR.

(B and C) AMs isolated as in (A) were in vitro stimulated with *S. pneumoniae* (MOI 100). The induction of *Cxcl1* was quantified by RT-PCR (B), and supernatant protein levels were determined by ELISA (C).

(D) CD45.1 WT monocytes were intra-tracheally transferred to WT and *Il13*$^{-/-}$ CD45.2 recipients, and bronchoalveolar cells were harvested by lavage 2 weeks later. FACS-sorted recipient AMs and monocyte-derived AMs were analyzed for expression of M2 polarization markers by RT-PCR.

(E–G) WT and *Il13*$^{-/-}$ mice were i.n. infected with *S. pneumoniae* and sacrificed after 6 hr. PMN numbers in BALF were assessed on cytospins (E) and in lungs by FACS analysis (CD45^{+}SSC^{hi}FSC^{hi}CD11b^{+} Ly6G^{+}) (F). Lung CXCL1 was quantified by ELISA (G).

(H–K) WT and *Il13*$^{-/-}$ mice were i.n. infected with *S. pneumoniae* and sacrificed after 48 hr. CFU counts in lung homogenates (H) and blood (I). Lung CXCL1 was quantified by ELISA (J). H&E-stained lung sections were scored by a pathologist (see Experimental Procedures) (K, left). Representative H&E lung sections (K, right). Scale bars represent 180 μm.

Data are representative of at least three independent experiments with four (A–C) and seven or eight (E–K) mice per group. Data in (D) are from a single experiment with six mice per group. PCR values were normalized to *Hprt* and expressed as fold change versus indicated control. Mean ± SEM are depicted; *$p < 0.05$, **$p < 0.01$, ***$p < 0.001$, and ****$p < 0.0001$. BAL, bronchoalveolar lavage; CFU, colony-forming units; p.i., post-infection; PMN, polymorphonuclear cells.

Collectively, these data show that pulmonary ILC2-derived IL-13 maintains lung resident AMs in an M2 state in healthy adult mice.

ILC2s Maintain the M2 Polarization of AMs Early in Development and in Adult Lungs

We then asked if the congenital absence of ILC2s would mirror the phenotype observed in *Il13*$^{-/-}$ mice. AMs extracted from newborn (P7) and adult *Il7r*^{Cre}*Rora*$^{sg/fl}$ mice, congenitally deficient in lung resident ILC2s (Oliphant et al., 2014) (Figure 7A), showed a reduced expression of M2 markers (Figure 7B, 7C and S7A) and increased *Cxcl1* and *Tnf* expression when stimulated with *S. pneumoniae* (Figure 7D). Infection of *Il7r*^{Cre}*Rora*$^{sg/fl}$ mice with *S. pneumoniae* resulted in increased neutrophil influx and higher lung CXCL1 levels 6 hr post-infection (Figures 7E, 7F, and S7B). This translated into an improved bacterial clearance with reduced systemic dissemination of pneumococci (Figures 7G and 7H), lower pulmonary CXCL1 levels (Figure 7I), and less severe lung infiltrates in *Il7r*^{Cre}*Rora*$^{sg/fl}$

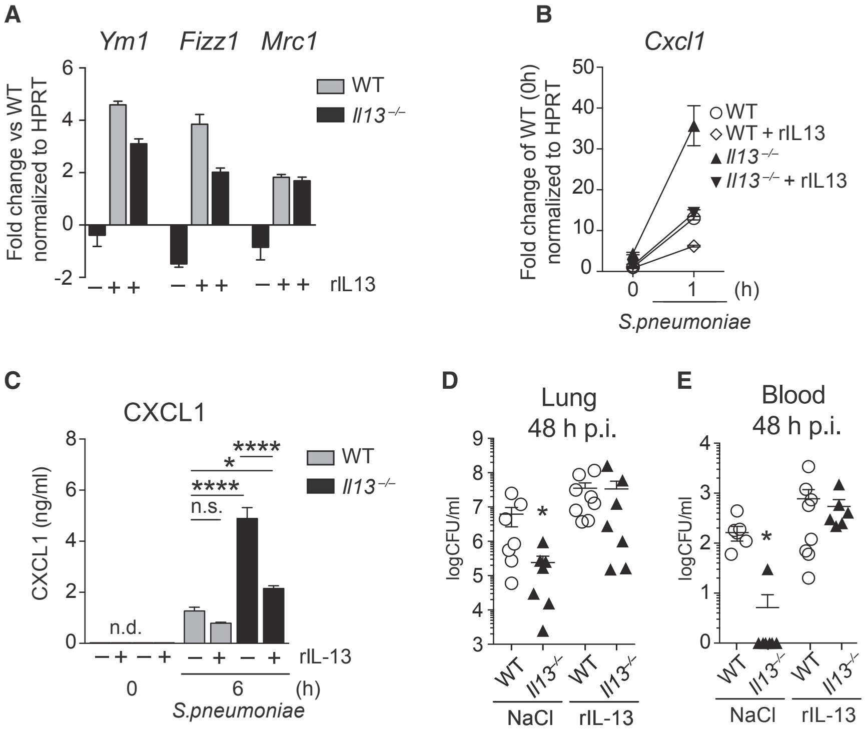

Figure 5. Intranasal rmIL-13 Treatment Reversed the Inflammatory Phenotype of AMs in *Il13*$^{-/-}$ Mice and the Responses to *S. pneumoniae*

(A–C) WT and *Il13*$^{-/-}$ mice were treated daily with rmIL-13 (6 ng in 50 μL NaCl) i.n., and AMs were isolated by bronchoalveolar lavage on day 3. M2 markers were assessed by RT-PCR (A). Cultured AMs were stimulated with *S. pneumoniae* (MOI 100), and fold induction of *Cxcl1* was measured by RT-PCR (B). AMs were treated as in (B), and CXCL1 protein was quantified by ELISA (C).

(D and E) WT and *Il13*$^{-/-}$ mice were treated with mrIL-13 as in (A)–(C), infected i.n. with *S. pneumoniae* on day 3, and sacrificed after 48 hr. CFU counts in lung homogenates (D) and blood (E) are shown.

Data in (A)–(C) are representative of two independent experiments with four mice per group. Data in (D) and (E) are from a single experiment with eight mice per group. Mean ± SEM are depicted; *p < 0.05 and ****p < 0.0001. BALF, bronchoalveolar lavage fluid; CFU, colony forming units; i.n., intranasal; p.i., post-infection.

mice 48 hr after infection (Figure 7J). We concluded that the congenital absence of ILC2s impacted on the M1 versus M2 polarization of AMs in neonatal and adult mice, with implications on the ability to fight bacterial lung infections.

We then asked to which degree ILC2s might contribute environmental signals to shape the functionality of bone-marrow-derived AMs (Lavin et al., 2014) and generated bone marrow chimeras using ILC2-deficient *Rora*$^{sg/sg}$ mice as donors (Wong et al., 2012) (Figures 7K and 7L). AMs isolated from WT/*Rora*$^{sg/sg}$ chimeras expressed lower levels of the M2 markers *Arg1* and *Fizz1* (Figure 7M) and higher levels of *Cxcl1* and *Tnf* in response to *S. pneumoniae* (Figure 7N). In vivo, WT/*Rora*$^{sg/sg}$ chimeras exhibited an augmented early inflammatory response upon pneumococcal infection (Figures 7O, 7P, and S7C). Collectively, lung ILC2s convey important cues that maintain quiescence by shaping the functional state of lung macrophages at homeostasis.

DISCUSSION

With the first breath, lungs are suddenly exposed to the external environment, therefore requiring regulatory forces in place to avoid continuous inflammatory reactions to environmental stimuli. Here, we show a perinatal wave of IL-33-mediated expansion and activation of ILC2s, resulting in an IL-13-driven polarization of newly differentiating AMs to an M2 phenotype. This exerts important homeostatic functions that contribute to a quiescent lung environment shortly after birth and throughout adult life.

AEC2 are the main source of IL-33, as shown earlier (Hardman et al., 2013; Pichery et al., 2012) and further confirmed by a recent study of developing AEC2 in embryonic lungs (Treutlein et al., 2014). Even though the mode of homeostatic IL-33 release remains to be elucidated, the mechanical stress induced by physiological ventilation possibly contributes to pulmonary IL-33 (Martin and Martin, 2016), and we discovered that exposing E19 lungs to negative pressure was sufficient to induce IL-33. Moreover, the release of bioactive IL-33 from living cells upon encounter of environmental allergens, extracellular ATP, or mechanical stress has been reported (Chen et al., 2015; Kakkar et al., 2012; Kouzaki et al., 2011; Sanada et al., 2007).

IL-33 and ILC2s are increasingly recognized as fundamental regulators of tissue homeostasis (Molofsky et al., 2015; von Moltke and Locksley, 2014). As such, recent reports described an IL-33-driven, ILC2-dependent mechanism for adipose tissue homeostasis, which involves the presence of eosinophils and M2 macrophages (Lee et al., 2015; Qiu et al., 2014). Excitingly, perinatal IL-33 induction was recently found to license adipocytes for uncoupled respiration and thermoregulation after birth (Odegaard et al., 2016).

The IL-33- and ILC2-dependent physiological type 2 milieu that we describe might play a role in the reportedly exaggerated airway hyperreactivity upon house dust mite exposure in newborns and strengthens the concept of a “window of immune development” (Gollwitzer et al., 2014). In fact, while this article was under revision, a report demonstrated a casual link between perinatal IL-33 induction and asthma (de Kleer et al., 2016). Here, we propose a unique and homeostatic role for ILC2s in shaping the lung immune environment in early life, as the appearance of activated ILC2s around P3 gradually de-activated AMs.

While type 2 responses, as seen upon helminth infections, are known to impair defenses against mycobacteria (Monin et al., 2015; Salgame et al., 2013), we now report that even homeostatic type 2 conditions impact on lung immunity, illustrated by

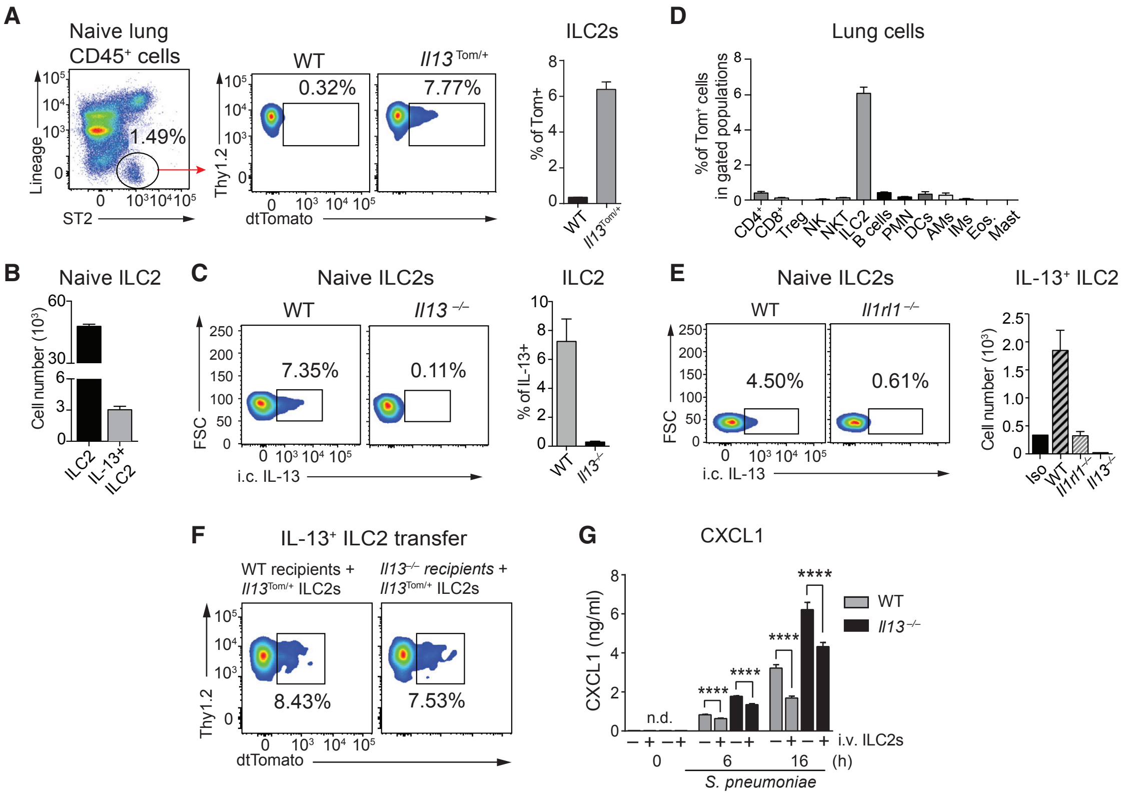

Figure 6. ILC2 Are the Only Cells Producing IL-13 in the Lung at Homeostasis

(A) IL-13 expression in ILC2s (Lin$^-$ ST2$^+$ ICOS$^+$ Thy1.2$^+$ CD25$^+$) assessed by flow cytometry in adult, naive *Il13$^{Tom/+}$* mice. Representative plots and percentage of tdTomato$^+$ ILC2s are shown.

(B and C) ILC2s and IL-13$^+$ ILC2s were quantified by flow cytometry and intracellular staining for IL-13 in naive WT lungs. (B) Absolute numbers of total lung ILC2s (Lin$^-$ ST2$^+$ ICOS$^+$ Thy1.2$^+$ CD25$^+$) and IL-13$^+$ ILC2s. (C) Representative plots of IL-13$^+$ ILC2s gated as in (A) and percentage of IL-13-producing ILC2s (right).

(D) Lung cell populations were tested by FACS for IL-13 expression in healthy adult *Il13$^{Tom/+}$* mice. Gating strategies are shown in Table S2.

(E) IL-13 production by lung ILC2s in WT, *Il1rl1$^{-/-}$*, and *Il13$^{-/-}$* assessed by intracellular staining using flow cytometry (Iso, isotype control); representative plots and absolute numbers are depicted.

(F and G) ILC2s were first expanded in lungs of *Il13$^{Tom/+}$* mice via i.n. administration of rmIL-33 (0.5 μg/50 μL for 5 days), and then sorted Tom$^+$ ILC2s were transferred intravenous to WT and *Il13$^{-/-}$* mice. (F) Representative FACS plots showing the homing of *Il13$^{Tom/+}$* ILC2s in lungs 5 days after adoptive transfer. (G) AMs were isolated by bronchoalveolar lavage from WT and *Il13$^{-/-}$* recipients 5 days after adoptive transfer and in vitro stimulated with *S. pneumoniae* (MOI 100), and CXCL1 release was assessed by ELISA in supernatants.

Data are representative of three (A–C), two (E–G), and one (D) independent experiments with four mice per group. Mean ± SEM are depicted; ****$p < 0.0001$. BAL, bronchoalveolar lavage; i.c., intracellular; i.v., intravenous; FSC, forward scatter.

reduced lung inflammation upon LPS challenge and a delayed clearance of medically important lung pathogens such as *S. pneumoniae*.

Our experiments in mice congenitally deficient in ILC2s corroborated the concept that ILC2s affected the AM phenotype from birth until adult life. Analysis of bone marrow chimeras using *Rora$^{sg/sg}$* mice further demonstrated that pulmonary ILC2s provided essential, tissue-specific signals to even polarize bone marrow precursors that arrive in lungs. Supporting our notion that ILC2s contribute to the in vivo phenotype of AMs, a recent publication identified tissue-specific transcriptional signatures of resident macrophages, and found AMs to be characterized by two IL-13- and IL-5-inducible genes, namely *Ym1* (*Chi3l3*) and *Car4*, respectively (Lavin et al., 2014).

In conclusion, we show that IL-33-driven ILC2 activation dominates the lung milieu early after birth by inducing a type-2 immune environment. Lung resident ILC2s are major contributors to the phenotype and function of tissue resident AMs at homeostasis, favoring a quiescent immune environment. While this effect might prove beneficial at steady state and upon sterile lung injury, it comes at the expense of a delayed response to the common lung pathogen *S. pneumoniae.*

EXPERIMENTAL PROCEDURES

Mice

Il13$^{-/-}$ (McKenzie et al., 1998), *Il13 $^{tdTomato/+}$* (Barlow et al., 2012) (referred as *Il13$^{Tom/+}$*), *Il1rl1$^{-/-}$* (Townsend et al., 2000), *Il5$^{-/-}$* (Kopf et al., 1996), *Rag2$^{-/-}$* (Shinkai et al., 1992), *Il7r^{Cre}* (Schlenner et al., 2010), *Rora$^{+/fl}$* (Oliphant et al., 2014), and Staggerer *Rora$^{sg/+}$* mice (Jackson Laboratories) were on a C57BL/6 background. We obtained *Il7r^{Cre}Rora$^{sg/fl}$* mice (experimental) or *Il7r^{Cre}Rora$^{+/fl}$* littermate controls by crossing *Il7r^{Cre}* with *Rora$^{+/fl}$* and *Rora$^{sg/+}$*. *Il33$^{Cit/+}$* (Hardman et al., 2013) mice and *Il5$^{Cer/+}$* mice (Saunders et al., 2016)

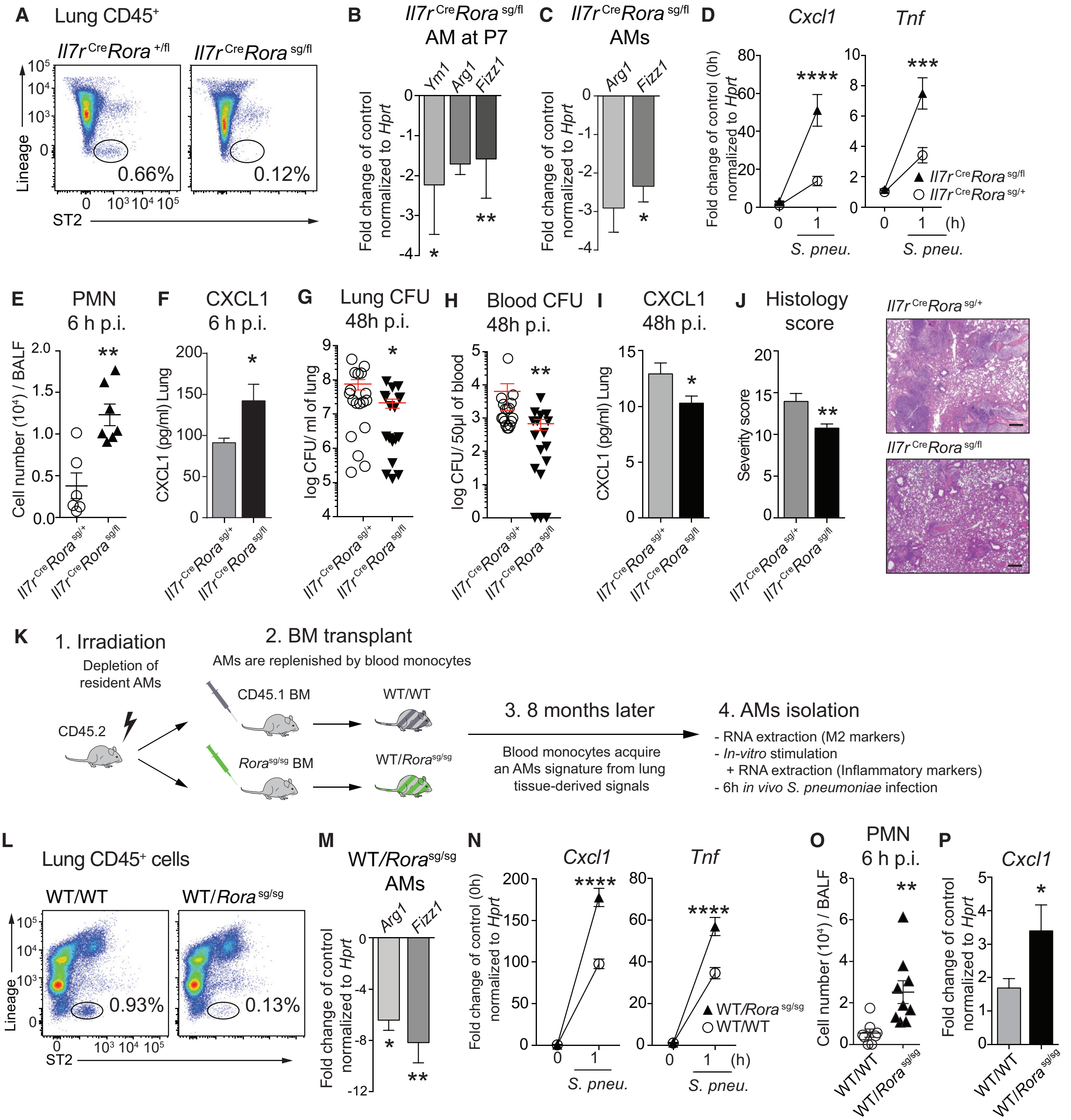

Figure 7. Lung Resident ILC2s Polarize Tissue Resident AMs toward an M2 Phenotype and Dampen Early Inflammatory Responses against Bacteria

(A) Flow cytometry plots of ILC2s in naive *Il7r*^{Cre}*Rora*$^{sg/fl}$ mice and *Il7r*^{Cre}*Rora*$^{+/fl}$ controls.

(B) AMs isolated by flow cytometry from healthy *Il7r*^{Cre}*Rora*$^{sg/fl}$ mice and controls at P7 and M2 markers evaluated by RT-PCR.

(C and D) AMs isolated by bronchoalveolar lavage from healthy adult *Il7r*^{Cre}*Rora*$^{sg/fl}$ mice and controls. (C) M2 markers evaluated by RT-PCR. (D) Primary AMs stimulated for 1 hr with *S. pneumoniae* (MOI 100) to assess the induction of *Cxcl1* and *Tnf*.

(E–J) *Il7r*^{Cre}*Rora*$^{sg/fl}$ mice and controls were i.n. infected with *S. pneumoniae* (10^5 CFUs) and sacrificed after 6 hr (E and F) or 48 hr (G–J). (E) PMN influx on BALF cytospins. (F) CXCL1 induction in whole-lung homogenate. (G and H) CFUs in lung (G) and in blood (H). (I) CXCL1 induction in whole-lung homogenate. (J) H&E-stained lung sections were scored by a pathologist (see Experimental Procedures) (J, left). Representative H&E lung sections (J, right). Scale bars represent 180 μm.

(legend continued on next page)

were on a BALB/c background. Mice were bred in a specific pathogen-free (SPF) facility and all mice were matched for age, gender and background in individual experiments. All animal experiments were approved by the Austrian Federal Ministry of Sciences and Research (BMWFW-66.009/0122-II/3b/2013) and the UK Home Office.

Isolation, Culture, and Stimulation of AMs

AMs from newborn mice were isolated by cell sorting using an FACSAria II (BD Biosciences) by gating on viable CD45^{+}F4/80^{+}CD11b^{low}CD11c^{+} SiglecF^{+} Ly6C^{-} cells. In adult mice, AMs were isolated by bronchoalveolar lavage followed by cell adhesion. Purity of isolated AM with both methods was consistently >95%. AMs were stimulated in RPMI containing 3% fetal calf serum (FCS) with heat-inactivated *S. pneumoniae* at a MOI 100 or *S. aureus* LTA (10 μg/mL). In Figures 3E–3G, 4A–4D, 5A–5C, 7B–7D and 7M, and 7N, cells were pooled from three or four mice per group and analyzed in technical quadruplicates.

Cytokine Administration

Recombinant mouse IL-13 and IL-33 were purchased from BioLegend. Anesthetized mice were treated daily with rmIL-13 (6 ng/50 μL NaCl for 3 consecutive days) or rmIL-33 (0.5 μg/50 μL NaCl for 5 consecutive days). Mice were sacrificed 1 hr after the last administration.

Adoptive Transfer of ILC2s

Lung ILC2s were FACS purified as defined by lineage^{-} (CD3a, CD4, CD8a, CD19, CD11c, CD11b, Gr1, FcεR1, CD49b), Thy1.2^{+} ST2^{+} ICOS^{+} and Tom^{+} from *Il13*$^{Tom/+}$ mice that had been treated intranasally (i.n.) with rmIL-33 for 5 days. Cells were transferred intravenously to *Il13*$^{-/-}$ or WT mice recipients (1×10^{5} cells per mouse) and assessed for localization in lungs 5 days later.

Generation of Bone Marrow Chimeras

6-week-old CD45.2 *Rora*$^{sg/sg}$ or WT littermates served as bone marrow donors. CD45.1 recipients were irradiated (9 Gy) and reconstituted on the same day with 2×10^{6} bone marrow cells per recipient by intravenous injection. Mice were analyzed for reconstitution and absence of lung resident ILC2s after 8 months.

Murine Pneumonia Model

Mice were infected i.n. with 10^{5} CFUs *S. pneumoniae* serotype 3 (ATCC 6303) as described (Sharif et al., 2014; Warszawska et al., 2013), or with 5×10^{7} CFUs *S. aureus* (USA300). Acute lung injury was induced by administration of 100 ng LPS i.n. (*E. coli* O55:B5). BALF was collected, cells were counted with an automated cell counter (Z2 Coulter Counter, Beckman), and Giemsa-stained cytospin preparations were used for differential cell counts. Lung tissues were homogenized in sterile saline using a Precellys 24TM (Peqlab), and lung colony-forming units (CFUs) were determined by 10-fold serial dilutions of homogenates on blood agar plates. An aliquot of lung homogenates was incubated in RA1 buffer (Macherey-Nagel) containing 10% of beta-mercaptoethanol (Calbiochem) and stored at $-80°$ for RNA extraction. The remaining lung homogenates were incubated in Greenberger lysis buffer as described previously (Sharif et al., 2014), and supernatants were stored at $-20°$C until cytokines were assayed.

Pneumonia Severity Score

Paraffin-embedded lung sections were stained with H&E and scored by a trained pathologist who was blinded to experimental groups. The final pneumonia score was the sum of the following parameters: severity of pleuritis, interstitial inflammation, edema, and thrombi formation were scored as 0 = absent, 1 = mild, 2 = moderately severe, 3 = severe; bronchitis was scored as 1 if present; endotheliitis was scored as 0 = absent, 2 = present, 3 = present with endothelial wall necrosis; the existence of a lobar confluent infiltrate was scored as 1, and a score of 0.5 was added for every infiltrate covering 10% of the lung area.

Statistical Analysis

Data are expressed as mean ± SEM. Statistical significance in two-group comparisons was assessed with an unpaired Student's t test. When indicated, a Mann-Whitney *U* test was used for analysis of nonparametric data. For multivariable comparisons we performed a one-way ANOVA followed by Sidak's multiple comparison test. Results were analyzed with Graph Pad Prism software version 6, and a $p < 0.05$ was regarded as statistically significant.

SUPPLEMENTAL INFORMATION

Supplemental Information includes Supplemental Experimental Procedures, seven figures, and three tables and can be found with this article online at http://dx.doi.org/10.1016/j.celrep.2017.01.071.

AUTHOR CONTRIBUTIONS

S. Saluzzo and S.K. conceived the study. A.N.J.M. hosted S. Saluzzo, contributed to experimental design, and provided critical reagents. S. Saluzzo, A.-D.G., B.M.J.R., R.M., S. Scanlon, P.S., K.L., A.H., O.S., A.K., J.M.W., and H.J. performed the experiments or contributed to experimental design, reagents, and analysis. I.M. scored histological slides. S. Scanlon performed the immunofluorescence experiments. R.M. analyzed the newborns alveolarization. A.-D.G. and B.M.J.R. contributed equally to this work. S. Saluzzo and S.K. wrote the manuscript with contributions from A.N.J.M., B.M.J.R., O.S., and S. Scanlon. A.N.J.M and S.K. are joint senior authors.

ACKNOWLEDGMENTS

We thank Hans-Reimer Rodewald for providing us with *Il7Rα*^{Cre} mice. We thank the staff of the animal facility of the Medical University of Vienna (AT) and of the Ares facility in Cambridge (UK) for their technical assistance. We thank Timotheus Y.F. Halim for precious advice. This work was supported by the Austrian Science Funds (FWF) within the doctoral program Cell Communication in Health and Disease (W1205) and within the ERA-Infect framework (I1620) (to S.K.), the Vienna Science and Technology Fund (WWTF and LS11-008) (to S.K.), and grants from the MRC (U105178805) and Wellcome Trust (100963/Z/13/Z) (to A.N.J.M.).

Received: June 3, 2016
Revised: December 27, 2016
Accepted: January 26, 2017
Published: February 21, 2017

REFERENCES

Barlow, J.L., Bellosi, A., Hardman, C.S., Drynan, L.F., Wong, S.H., Cruickshank, J.P., and McKenzie, A.N.J. (2012). Innate IL-13-producing nuocytes arise during allergic lung inflammation and contribute to airways hyperreactivity. J. Allergy Clin. Immunol. *129*, 191–198.e4.

(K–N) CD45.2 recipients were lethally irradiated and transplanted with WT or *Rora*$^{sg/sg}$ bone marrow and sacrificed 8 months later. (K) Experimental setup. (L) Representative FACS plots of ILC2s in healthy WT/WT and WT/*Rora*$^{sg/sg}$ bone marrow chimeras. (M) AMs isolated via bronchoalveolar lavage and assessed for M2 markers by RT-PCR or (N) stimulated with *S. pneumoniae* (MOI 100) to evaluate *Cxcl1* and *Tnf* induction.
(O and P) WT/WT and WT/*Rora*$^{sg/sg}$ chimeras were infected with 10^{5} CFUs *S. pneumoniae* and sacrificed after 6 hr to assess (O) PMN influx and (P) *Cxcl1* induction in lung tissue.
Data are representative of at least two independent experiments with four (A–D, M, and N) and seven or eight (E–J, O, and P) mice per group. Data in (G) and (H) are pooled from two independent experiments. Mean ± SEM are depicted; *$p \leq 0.05$, **$p < 0.01$, ***$p < 0.001$, and ****$p < 0.0001$. BALF, bronchoalveolar lavage fluid; CFU, colony-forming units; p.i., post-infection; PMN, polymorphonuclear cells.

Beers, M.F., and Morrisey, E.E. (2011). The three R's of lung health and disease: repair, remodeling, and regeneration. J. Clin. Invest. *121*, 2065–2073.

Brestoff, J.R., Kim, B.S., Saenz, S.A., Stine, R.R., Monticelli, L.A., Sonnenberg, G.F., Thome, J.J., Farber, D.L., Lutfy, K., Seale, P., and Artis, D. (2015). Group 2 innate lymphoid cells promote beiging of white adipose tissue and limit obesity. Nature *519*, 242–246.

Chang, Y.-J., Kim, H.Y., Albacker, L.A., Baumgarth, N., McKenzie, A.N., Smith, D.E., Dekruyff, R.H., and Umetsu, D.T. (2011). Innate lymphoid cells mediate influenza-induced airway hyper-reactivity independently of adaptive immunity. Nat. Immunol. *12*, 631–638.

Chen, W.H., Toapanta, F.R., Shirey, K.A., Zhang, L., Giannelou, A., Page, C., Frieman, M.B., Vogel, S.N., and Cross, A.S. (2012). Potential role for alternatively activated macrophages in the secondary bacterial infection during recovery from influenza. Immunol. Lett. *141*, 227–234.

Chen, W.-Y., Hong, J., Gannon, J., Kakkar, R., and Lee, R.T. (2015). Myocardial pressure overload induces systemic inflammation through endothelial cell IL-33. Proc. Natl. Acad. Sci. USA *112*, 7249–7254.

Chien, Y.-W., Klugman, K.P., and Morens, D.M. (2009). Bacterial pathogens and death during the 1918 influenza pandemic. N. Engl. J. Med. *361*, 2582–2583.

Chiu, C., and Openshaw, P.J. (2015). Antiviral B cell and T cell immunity in the lungs. Nat. Immunol. *16*, 18–26.

De Filippo, K., Dudeck, A., Hasenberg, M., Nye, E., van Rooijen, N., Hartmann, K., Gunzer, M., Roers, A., and Hogg, N. (2013). Mast cell and macrophage chemokines CXCL1/CXCL2 control the early stage of neutrophil recruitment during tissue inflammation. Blood *121*, 4930–4937.

de Kleer, I.M., Kool, M., de Bruijn, M.J.W., Willart, M., van Moorleghem, J., Schuijs, M.J., Plantinga, M., Beyaert, R., Hams, E., Fallon, P.G., et al. (2016). Perinatal activation of the interleukin-33 pathway promotes type 2 immunity in the developing lung. Immunity *45*, 1285–1298.

Gollwitzer, E.S., Saglani, S., Trompette, A., Yadava, K., Sherburn, R., McCoy, K.D., Nicod, L.P., Lloyd, C.M., and Marsland, B.J. (2014). Lung microbiota promotes tolerance to allergens in neonates via PD-L1. Nat. Med. *20*, 642–647.

Gordon, S., and Martinez, F.O. (2010). Alternative activation of macrophages: mechanism and functions. Immunity *32*, 593–604.

Guery, L., Benikhlef, N., Gautier, T., Paul, C., Jego, G., Dufour, E., Jacquel, A., Cally, R., Manoury, B., Vanden Berghe, T., et al. (2011). Fine-tuning nucleophosmin in macrophage differentiation and activation. Blood *118*, 4694–4704.

Guilliams, M., De Kleer, I., Henri, S., Post, S., Vanhoutte, L., De Prijck, S., Deswarte, K., Malissen, B., Hammad, H., and Lambrecht, B.N. (2013). Alveolar macrophages develop from fetal monocytes that differentiate into long-lived cells in the first week of life via GM-CSF. J. Exp. Med. *210*, 1977–1992.

Halim, T.Y.F., Steer, C.A., Mathä, L., Gold, M.J., Martinez-Gonzalez, I., McNagny, K.M., McKenzie, A.N.J., and Takei, F. (2014). Group 2 innate lymphoid cells are critical for the initiation of adaptive T helper 2 cell-mediated allergic lung inflammation. Immunity *40*, 425–435.

Hardman, C.S., Panova, V., and McKenzie, A.N.J. (2013). IL-33 citrine reporter mice reveal the temporal and spatial expression of IL-33 during allergic lung inflammation. Eur. J. Immunol. *43*, 488–498.

Hogan, B.L.M., Barkauskas, C.E., Chapman, H.A., Epstein, J.A., Jain, R., Hsia, C.C.W., Niklason, L., Calle, E., Le, A., Randell, S.H., et al. (2014). Repair and regeneration of the respiratory system: complexity, plasticity, and mechanisms of lung stem cell function. Cell Stem Cell *15*, 123–138.

Hussell, T., and Bell, T.J. (2014). Alveolar macrophages: plasticity in a tissue-specific context. Nat. Rev. Immunol. *14*, 81–93.

Kakkar, R., Hei, H., Dobner, S., and Lee, R.T. (2012). Interleukin 33 as a mechanically responsive cytokine secreted by living cells. J. Biol. Chem. *287*, 6941–6948.

Kearley, J., Silver, J.S., Sanden, C., Liu, Z., Berlin, A.A., White, N., Mori, M., Pham, T.-H., Ward, C.K., Criner, G.J., et al. (2015). Cigarette smoke silences innate lymphoid cell function and facilitates an exacerbated type I interleukin-33-dependent response to infection. Immunity *42*, 566–579.

Kim, E.Y., Battaile, J.T., Patel, A.C., You, Y., Agapov, E., Grayson, M.H., Benoit, L.A., Byers, D.E., Alevy, Y., Tucker, J., et al. (2008). Persistent activation of an innate immune response translates respiratory viral infection into chronic lung disease. Nat. Med. *14*, 633–640.

Kim, H.Y., Chang, Y.J., Subramanian, S., Lee, H.H., Albacker, L.A., Matangkasombut, P., Savage, P.B., McKenzie, A.N.J., Smith, D.E., Rottman, J.B., et al. (2012). Innate lymphoid cells responding to IL-33 mediate airway hyperreactivity independently of adaptive immunity. J. Allergy Clin. Immunol. *129*, 216–227.e6.

Kopf, M., Brombacher, F., Hodgkin, P.D., Ramsay, A.J., Milbourne, E.A., Dai, W.J., Ovington, K.S., Behm, C.A., Köhler, G., Young, I.G., and Matthaei, K.I. (1996). IL-5-deficient mice have a developmental defect in CD5+ B-1 cells and lack eosinophilia but have normal antibody and cytotoxic T cell responses. Immunity *4*, 15–24.

Kopf, M., Schneider, C., and Nobs, S.P. (2015). The development and function of lung-resident macrophages and dendritic cells. Nat. Immunol. *16*, 36–44.

Kouzaki, H., Iijima, K., Kobayashi, T., O'Grady, S.M., and Kita, H. (2011). The danger signal, extracellular ATP, is a sensor for an airborne allergen and triggers IL-33 release and innate Th2-type responses. J. Immunol. *186*, 4375–4387.

Lambrecht, B.N., and Hammad, H. (2015). The immunology of asthma. Nat. Immunol. *16*, 45–56.

Lamkanfi, M., and Dixit, V.M. (2009). IL-33 raises alarm. Immunity *31*, 5–7.

Lavin, Y., Winter, D., Blecher-Gonen, R., David, E., Keren-Shaul, H., Merad, M., Jung, S., and Amit, I. (2014). Tissue-resident macrophage enhancer landscapes are shaped by the local microenvironment. Cell *159*, 1312–1326.

Lee, M.W., Odegaard, J.I., Mukundan, L., Qiu, Y., Molofsky, A.B., Nussbaum, J.C., Yun, K., Locksley, R.M., and Chawla, A. (2015). Activated type 2 innate lymphoid cells regulate beige fat biogenesis. Cell *160*, 74–87.

Liew, F.Y., Pitman, N.I., and McInnes, I.B. (2010). Disease-associated functions of IL-33: the new kid in the IL-1 family. Nat. Rev. Immunol. *10*, 103–110.

Lu, J., Kang, J., Zhang, C., and Zhang, X. (2015). The role of IL-33/ST2L signals in the immune cells. Immunol. Lett. *164*, 11–17.

Mantovani, A., Sica, A., Sozzani, S., Allavena, P., Vecchi, A., and Locati, M. (2004). The chemokine system in diverse forms of macrophage activation and polarization. Trends Immunol. *25*, 677–686.

Martin, N.T., and Martin, M.U. (2016). Interleukin 33 is a guardian of barriers and a local alarmin. Nat. Immunol. *17*, 122–131.

McKenzie, G.J., Emson, C.L., Bell, S.E., Anderson, S., Fallon, P., Zurawski, G., Murray, R., Grencis, R., and McKenzie, A.N.J. (1998). Impaired development of Th2 cells in IL-13-deficient mice. Immunity *9*, 423–432.

Molofsky, A.B., Nussbaum, J.C., Liang, H.-E., Van Dyken, S.J., Cheng, L.E., Mohapatra, A., Chawla, A., and Locksley, R.M. (2013). Innate lymphoid type 2 cells sustain visceral adipose tissue eosinophils and alternatively activated macrophages. J. Exp. Med. *210*, 535–549.

Molofsky, A.B., Savage, A.K., and Locksley, R.M. (2015). Interleukin-33 in tissue homeostasis, injury, and inflammation. Immunity *42*, 1005–1019.

Monin, L., Griffiths, K.L., Lam, W.Y., Gopal, R., Kang, D.D., Ahmed, M., Rajamanickam, A., Cruz-Lagunas, A., Zúñiga, J., Babu, S., et al. (2015). Helminth-induced arginase-1 exacerbates lung inflammation and disease severity in tuberculosis. J. Clin. Invest. *125*, 4699–4713.

Monticelli, L.A., Sonnenberg, G.F., Abt, M.C., Alenghat, T., Ziegler, C.G., Doering, T.A., Angelosanto, J.M., Laidlaw, B.J., Yang, C.Y., Sathaliyawala, T., et al. (2011). Innate lymphoid cells promote lung-tissue homeostasis after infection with influenza virus. Nat. Immunol. *12*, 1045–1054.

Mund, S.I., Stampanoni, M., and Schittny, J.C. (2008). Developmental alveolarization of the mouse lung. Dev. Dyn. *237*, 2108–2116.

Murphy, J., Summer, R., Wilson, A.A., Kotton, D.N., and Fine, A. (2008). The prolonged life-span of alveolar macrophages. Am. J. Respir. Cell Mol. Biol. *38*, 380–385.

Neill, D.R., Wong, S.H., Bellosi, A., Flynn, R.J., Daly, M., Langford, T.K., Bucks, C., Kane, C.M., Fallon, P.G., Pannell, R., et al. (2010). Nuocytes represent a

new innate effector leukocyte that mediates type-2 immunity. Nature *464*, 1367–1370.

Nussbaum, J.C., Van Dyken, S.J., von Moltke, J., Cheng, L.E., Mohapatra, A., Molofsky, A.B., Thornton, E.E., Krummel, M.F., Chawla, A., Liang, H.-E., and Locksley, R.M. (2013). Type 2 innate lymphoid cells control eosinophil homeostasis. Nature *502*, 245–248.

Odegaard, J.I., Lee, M.-W., Sogawa, Y., Bertholet, A.M., Locksley, R.M., Weinberg, D.E., Kirichok, Y., Deo, R.C., and Chawla, A. (2016). Perinatal licensing of thermogenesis by IL-33 and ST2. Cell *166*, 841–854.

Okabe, Y., and Medzhitov, R. (2014). Tissue-specific signals control reversible program of localization and functional polarization of macrophages. Cell *157*, 832–844.

Oliphant, C.J., Hwang, Y.Y., Walker, J.A., Salimi, M., Wong, S.H., Brewer, J.M., Englezakis, A., Barlow, J.L., Hams, E., Scanlon, S.T., et al. (2014). MHCII-mediated dialog between group 2 innate lymphoid cells and CD4(+) T cells potentiates type 2 immunity and promotes parasitic helminth expulsion. Immunity *41*, 283–295.

Orr, A.W., Helmke, B.P., Blackman, B.R., and Schwartz, M.A. (2006). Mechanisms of mechanotransduction. Dev. Cell *10*, 11–20.

Peng, T., Frank, D.B., Kadzik, R.S., Morley, M.P., Rathi, K.S., Wang, T., Zhou, S., Cheng, L., Lu, M.M., and Morrisey, E.E. (2015). Hedgehog actively maintains adult lung quiescence and regulates repair and regeneration. Nature *526*, 578–582.

Pichery, M., Mirey, E., Mercier, P., Lefrancais, E., Dujardin, A., Ortega, N., and Girard, J.-P. (2012). Endogenous IL-33 is highly expressed in mouse epithelial barrier tissues, lymphoid organs, brain, embryos, and inflamed tissues: in situ analysis using a novel Il-33-LacZ gene trap reporter strain. J. Immunol. *188*, 3488–3495.

Price, A.E., Liang, H.-E., Sullivan, B.M., Reinhardt, R.L., Eisley, C.J., Erle, D.J., and Locksley, R.M. (2010). Systemically dispersed innate IL-13-expressing cells in type 2 immunity. Proc. Natl. Acad. Sci. USA *107*, 11489–11494.

Qiu, Y., Nguyen, K.D., Odegaard, J.I., Cui, X., Tian, X., Locksley, R.M., Palmiter, R.D., and Chawla, A. (2014). Eosinophils and type 2 cytokine signaling in macrophages orchestrate development of functional beige fat. Cell *157*, 1292–1308.

Rijavec, M., Volarevic, S., Osolnik, K., Kosnik, M., and Korosec, P. (2011). Natural killer T cells in pulmonary disorders. Respir. Med. *105* (*Suppl 1*), S20–S25.

Roediger, B., and Weninger, W. (2015). Group 2 innate lymphoid cells in the regulation of immune responses. Adv. Immunol. *125*, 111–154.

Salgame, P., Yap, G.S., and Gause, W.C. (2013). Effect of helminth-induced immunity on infections with microbial pathogens. Nat. Immunol. *14*, 1118–1126.

Sanada, S., Hakuno, D., Higgins, L.J., Schreiter, E.R., McKenzie, A.N.J., and Lee, R.T. (2007). IL-33 and ST2 comprise a critical biomechanically induced and cardioprotective signaling system. J. Clin. Invest. *117*, 1538–1549.

Saunders, S.P., Moran, T., Floudas, A., Wurlod, F., Kaszlikowska, A., Salimi, M., Quinn, E.M., Oliphant, C.J., Núñez, G., McManus, R., et al. (2016). Spontaneous atopic dermatitis is mediated by innate immunity, with the secondary lung inflammation of the atopic march requiring adaptive immunity. J. Allergy Clin. Immunol. *137*, 482–491.

Schlenner, S.M., Madan, V., Busch, K., Tietz, A., Läufle, C., Costa, C., Blum, C., Fehling, H.J., and Rodewald, H.R. (2010). Fate mapping reveals separate origins of T cells and myeloid lineages in the thymus. Immunity *32*, 426–436.

Schliehe, C., Flynn, E.K., Vilagos, B., Richson, U., Swaminathan, S., Bosnjak, B., Bauer, L., Kandasamy, R.K., Griesshammer, I.M., Kosack, L., et al. (2015). The methyltransferase Setdb2 mediates virus-induced susceptibility to bacterial superinfection. Nat. Immunol. *16*, 67–74.

Schmitz, J., Owyang, A., Oldham, E., Song, Y., Murphy, E., McClanahan, T.K., Zurawski, G., Moshrefi, M., Qin, J., Li, X., et al. (2005). IL-33, an interleukin-1-like cytokine that signals via the IL-1 receptor-related protein ST2 and induces T helper type 2-associated cytokines. Immunity *23*, 479–490.

Sharif, O., Gawish, R., Warszawska, J.M., Martins, R., Lakovits, K., Hladik, A., Doninger, B., Brunner, J., Korosec, A., Schwarzenbacher, R.E., et al. (2014). The triggering receptor expressed on myeloid cells 2 inhibits complement component 1q effector mechanisms and exerts detrimental effects during pneumococcal pneumonia. PLoS Pathog. *10*, e1004167.

Shinkai, Y., Rathbun, G., Lam, K.P., Oltz, E.M., Stewart, V., Mendelsohn, M., Charron, J., Datta, M., Young, F., Stall, A.M., et al. (1992). RAG-2-deficient mice lack mature lymphocytes owing to inability to initiate V(D)J rearrangement. Cell *68*, 855–867.

Talbot, T.R., Hartert, T.V., Mitchel, E., Halasa, N.B., Arbogast, P.G., Poehling, K.A., Schaffner, W., Craig, A.S., and Griffin, M.R. (2005). Asthma as a risk factor for invasive pneumococcal disease. N. Engl. J. Med. *352*, 2082–2090.

Townsend, M.J., Fallon, P.G., Matthews, D.J., Jolin, H.E., and McKenzie, A.N. (2000). T1/ST2-deficient mice demonstrate the importance of T1/ST2 in developing primary T helper cell type 2 responses. J. Exp. Med. *191*, 1069–1076.

Treutlein, B., Brownfield, D.G., Wu, A.R., Neff, N.F., Mantalas, G.L., Espinoza, F.H., Desai, T.J., Krasnow, M.A., and Quake, S.R. (2014). Reconstructing lineage hierarchies of the distal lung epithelium using single-cell RNA-seq. Nature *509*, 371–375.

van der Poll, T., and Opal, S.M. (2009). Pathogenesis, treatment, and prevention of pneumococcal pneumonia. Lancet *374*, 1543–1556.

von Moltke, J., and Locksley, R.M. (2014). I-L-C-2 it: type 2 immunity and group 2 innate lymphoid cells in homeostasis. Curr. Opin. Immunol. *31*, 58–65.

Warszawska, J.M., Gawish, R., Sharif, O., Sigel, S., Doninger, B., Lakovits, K., Mesteri, I., Nairz, M., Boon, L., Spiel, A., et al. (2013). Lipocalin 2 deactivates macrophages and worsens pneumococcal pneumonia outcomes. J. Clin. Invest. *123*, 3363–3372.

Wirtz, H.R., and Dobbs, L.G. (2000). The effects of mechanical forces on lung functions. Respir. Physiol. *119*, 1–17.

Woik, N., and Kroll, J. (2015). Regulation of lung development and regeneration by the vascular system. Cell. Mol. Life Sci. *72*, 2709–2718.

Wong, S.H., Walker, J.A., Jolin, H.E., Drynan, L.F., Hams, E., Camelo, A., Barlow, J.L., Neill, D.R., Panova, V., Koch, U., et al. (2012). Transcription factor RORα is critical for nuocyte development. Nat. Immunol. *13*, 229–236.

Cell Reports, Volume *18*

Supplemental Information

First-Breath-Induced Type 2 Pathways

Shape the Lung Immune Environment

Simona Saluzzo, Anna-Dorothea Gorki, Batika M.J. Rana, Rui Martins, Seth Scanlon, Philipp Starkl, Karin Lakovits, Anastasiya Hladik, Ana Korosec, Omar Sharif, Joanna M. Warszawska, Helen Jolin, Ildiko Mesteri, Andrew N.J. McKenzie, and Sylvia Knapp

Supplemental Figures and Legends

Figure S1

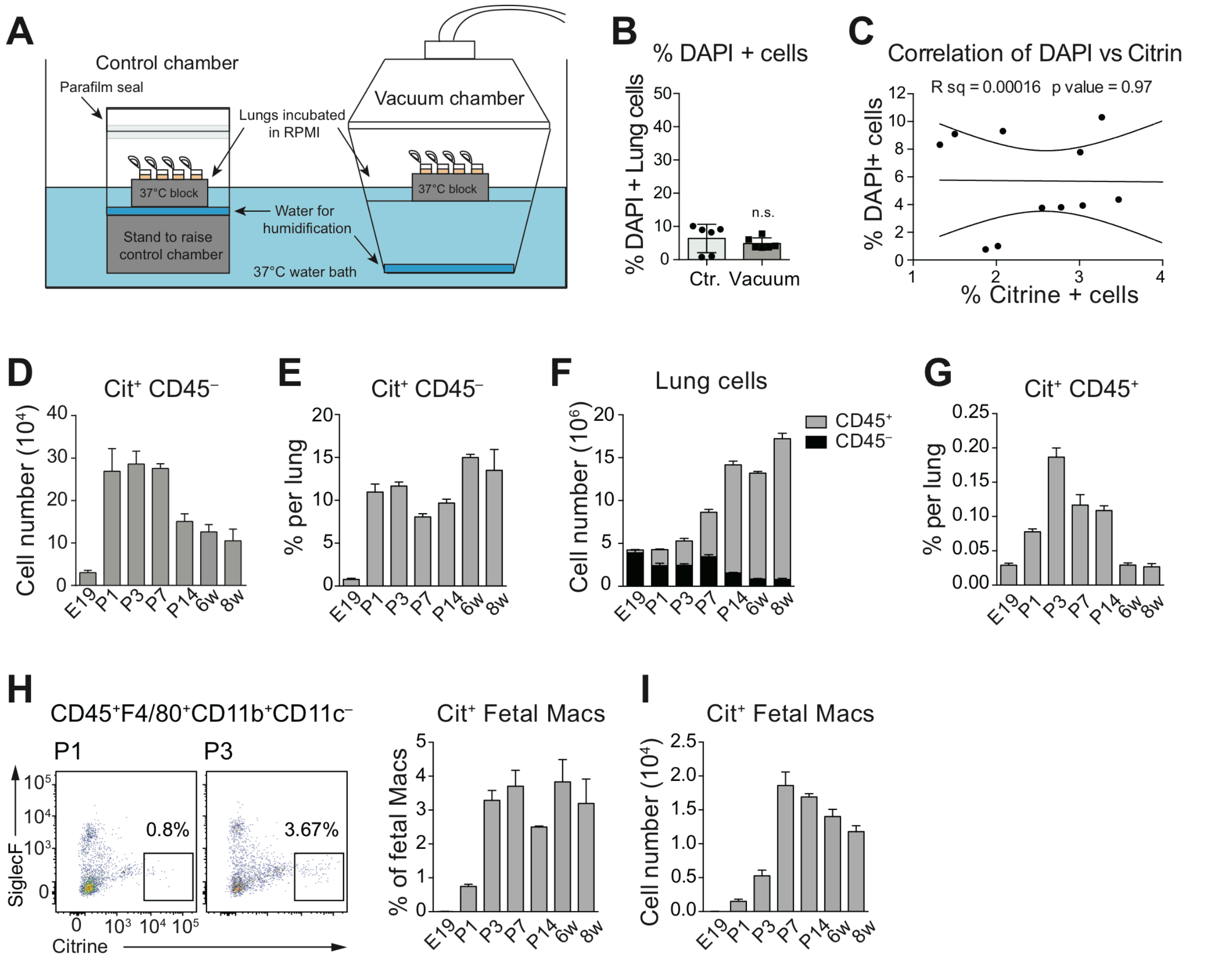

Figure S1. Integrated analysis of Citrine expression in lung cells of newborns and upon negative pressure. Related to Figure 1.

(A) Experimental set-up. Embryonic lungs (P19) were placed at 37°C and subjected to a negative pressure for 6h (see experimental procedures) (referring to Figure 1C, D, E).

(B) Viability of lung cells assessed by DAPI staining (referring to Figure 1C, D, E).

(C) Linear regression analysis of viability and IL-33 expression (Citrin) of embryonic lung cells subjected to negative pressure (vacuum) for 6h (referring to Figure 1C, D, E).

(D) Absolute numbers of Cit^+ $CD45^-$ lung cells (referring to Figure 1F lower panel and 1G).

(E) Proportion of $CD45^-$ cells that up-regulate Citrine at indicated time points (referring to Figure 1F lower panel)

(F) Relative contribution of $CD45^+$ and $CD45^-$ cells to total lung cell numbers from E19 to 8 weeks of age (referring to Figure 1F upper panel).

(G) Quantification of Citrine expression among CD45+ cells in newborn mice.

(H) Left: blots showing expression of Citrine among fetal macrophages gated as $CD45^+$ $F4/80^+$ $CD11b^+$ $CD11c^-$ $SiglecF^-$. Right: quantification of Citrine expression among fetal macrophages.

(I) Absolute numbers of lung fetal macrophages gated as in (H) at indicated time points.

Graph bars represent mean ± SEM. Data are representative of two independent experiments with 3-4 mice per time point.

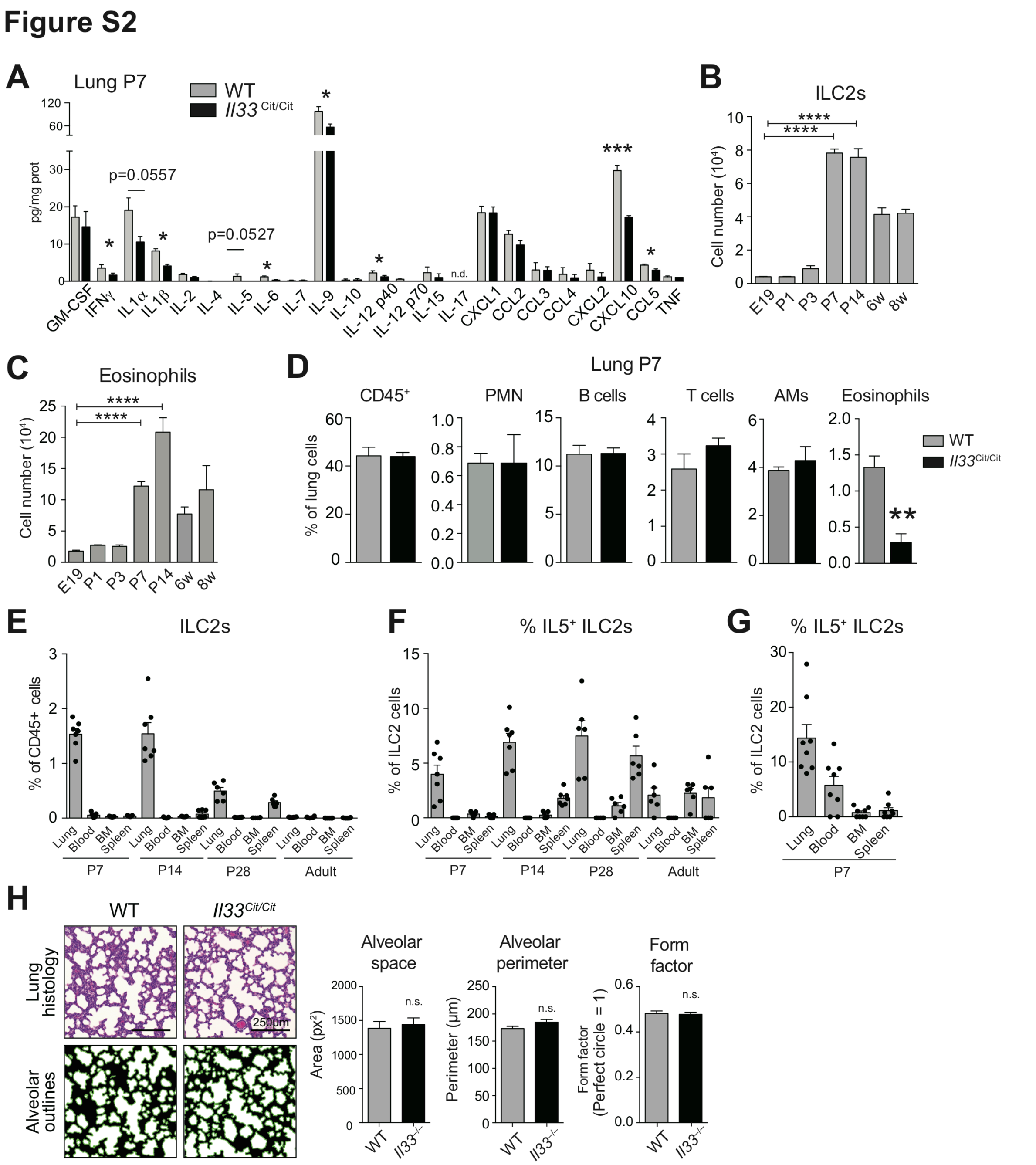

Figure S2. Integrated analysis of *Il33*$^{Cit/Cit}$ mice on P7, postnatal eosinophil expansion and perinatal alveolarization. Related to Figure 2.

(A) Absolute values of multiplex ELISA (referring to Figure 2A).

(B) Absolute numbers of lung ILC2s between E19 and week 8, (referring to Figure 2B).

(C) Absolute numbers of lung eosinophils between E19 and week 8, (referring to Figure 2E).

(D) Analysis of lung immune cell populations in WT and *Il33*$^{Cit/Cit}$ mice, depicted is the percentage of total lung cells, (referring to Figure 2F). PMN = polymorphonuclear cells gated as CD45^{+} Ly6G^{+}; B cells = CD45^{+} CD19^{+}; T cells = CD45^{+} CD3^{+}; AMs = CD11b^{-} SiglecF^{+} CD11c^{+}; eosinophils = CD11b^{+} SiglecF^{+} CD11c^{-}.

(E, F) Proportion of ILC2s (among CD45^{+}cells) and IL5-expressing ILC2s (*Il5*$^{Cer/+}$ mice) in indicated body compartments between P7 and adulthood.

(G) Quantification of IL5 production by ILC2s assessed by i.c. cytokine stain, (referring to Figure 2H).

(H) Lung alveolarization was quantified on H&E stained lung sections from WT and *Il33*$^{Cit/Cit}$ mice at P7 using the automatic image analysis software CellProfiler. For details see Supplemental experimental procedures. Graph bars represent mean ± SEM. *p < 0.05, ***p<0.001, ****p < 0.0001. Data are representative of two independent experiments with 4 mice per group and/or time point.

Figure S3

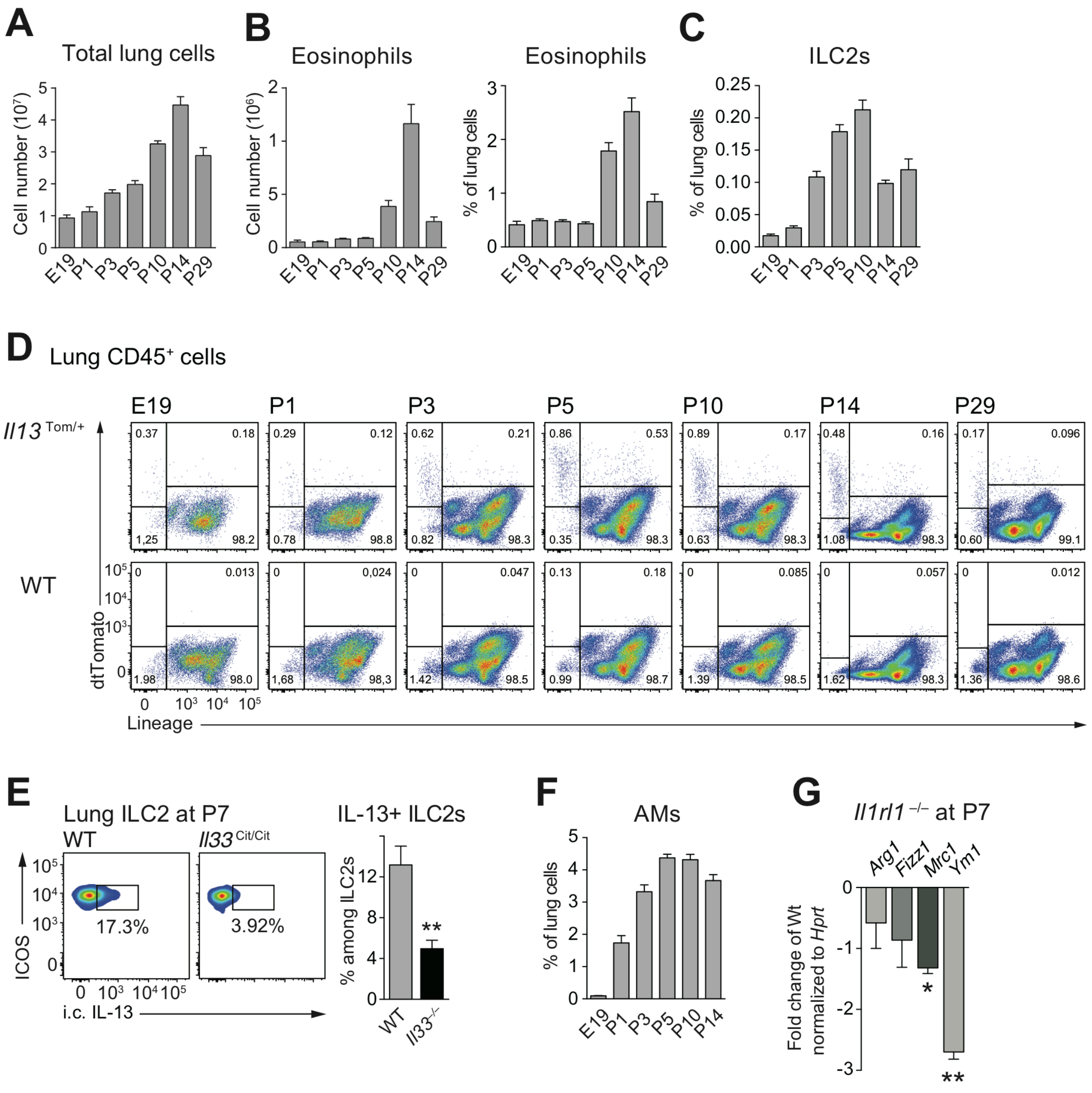

Figure S3. Lin⁻cells are the only IL-13 expressing cells in the first two weeks after birth. Related to Figure 3.

(A-C) Quantification of lung ILC2s and eosinophils at the indicated time points in *Il13*$^{Tom/+}$ mice. Of note, *Il13*$^{Tom/+}$ mice, which are on a C57BL/6 background, show the same cell influx dynamic as *Il33*$^{Cit/+}$ mice, which are on a Balb/c background, (referring to Figure 3A-D). (A) Absolute number of total lung cells. (B) Absolute numbers and percentages of eosinophils gated as in Figure 2E. (C) Percentage of ILC2s, gated as in Figure 2B.
(D) FACS of lung cells gated for Lineage (CD3, CD4, CD8, CD19, F4/80, CD11b, CD11c, FceRI, NK1.1, Ly6C/G) and *Il13*^{dtTomato} expression at indicated time points.
(E) IL-13 expression in lung ILC2s at P7 quantified by i.c. cytokine stain.
(F) Percentage of AMs gated as in Figure 3C, (referring to Figure 3C-D).
(G) AMs (F4/80$^+$CD11b$^-$CD11c$^+$SiglecF$^+$) were sorted on P7 from WT and *Il1rl1*$^{-/-}$ mice and M2 markers were assessed by RT-PCR.
Data are representative of two (A-F) and one (G) independent experiments with 3-4 mice per group. Bars represent mean ± SEM; *p < 0.05; **p < 0.01.

Figure S4

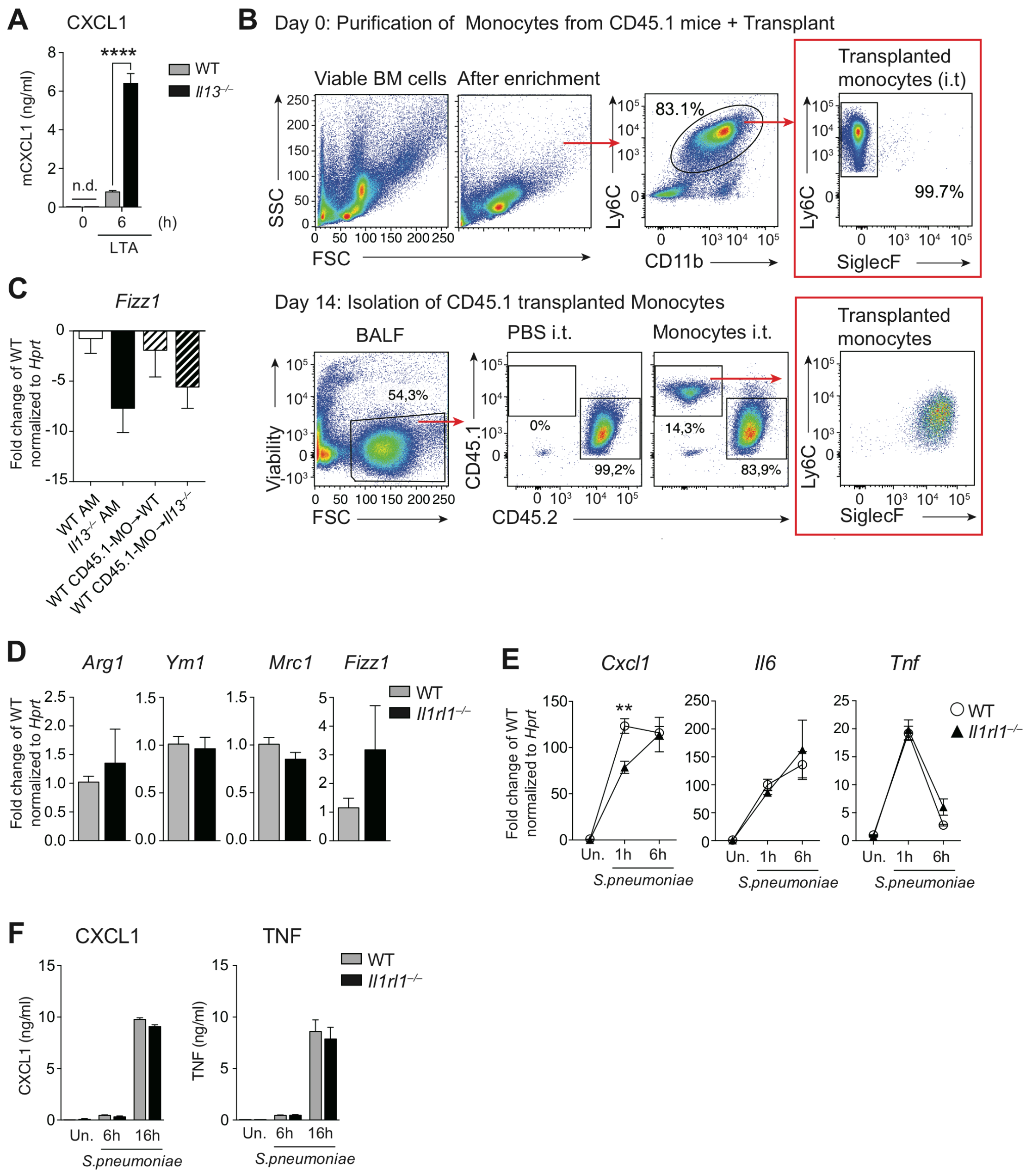

Figure S4. BM derived monocytes assume an AMs phenotype 2 weeks upon intratracheal transplant. *Il1rl1* alone plays no significant role in AM polarization in adult mice. Related to Fig 4.

(A) AMs from WT and *Il13*$^{-/-}$ mice were isolated by BAL and *in vitro* stimulated with LTA (10µg/ml). CXCL1 was quantified in supernatants, (refers to Figure 4B-C).

(B) FACS plots representing bone marrow derived monocytes isolated from WT CD45.1 mice and transferred i.t. into WT or *Il13*$^{-/-}$ mice. Two weeks later transferred monocytes showed upregulation of SiglecF, (refers to Fig 4D).

(C) *Fizz1* expression was quantified in FACS-sorted AMs upon monocytes transplant, (refers to Fig 4D.

(D, E) AMs isolated via BAL from WT and *Il1rl1*$^{-/-}$mice analyzed for M2 markers by RT-PCR (D), or *in vitro* stimulated with *S. pneumoniae* (MOI 100) and analyzed for cytokine expression by RT-PCR (E). Values were normalized to HPRT and are expressed as fold-change versus WT at t = 0h.

(F) AMs were isolated as in (D), and stimulated *in vitro* with *S. pneumoniae* (MOI 100) and CXCL1 and TNF protein levels were quantified in supernatants by ELISA.

Data are representative of at least two independent experiments with four (A and D-F) and 6-7 (B, C) mice per group and/or time point. Mean ± SEM are depicted; **p < 0.01, ****p < 0.0001. I.t. = intra-tracheally; MOI = multiplicity of infection; BAL = bronchoalveolar lavage.

Figure S5

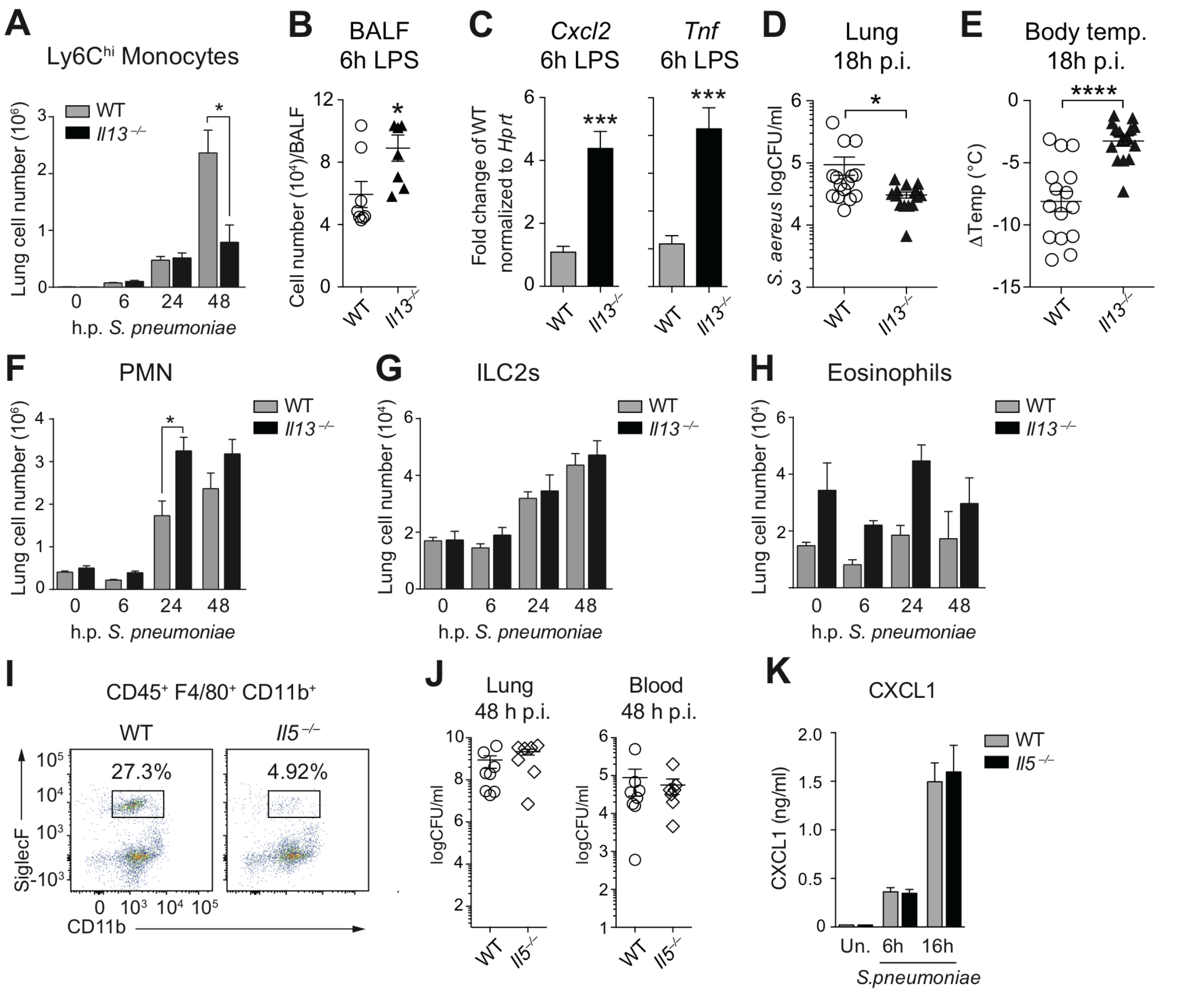

Figure S5. Broader role of IL-13 in the inflammatory response to LPS and *S. aureus*. Eosinophils do not impact on AM polarization, or responses to *S. pneumoniae*. Related to Fig 4.

(A) Numbers of lung Ly6C^{hi} monocytes (F4/80^{+} CD11b^{+} Ly6C^{hi}) during *S. pneumonia* infection, (refers to Figure 4E-K).

(B-C) Mice were treated i.n. with LPS (100ng/50μl) and harvested 6h later to assess cell influx in BALF (B) and cytokine expression in BALF (C).

(D-E) Mice were infected i.n. with *S. aureus* and assessed 18h later for body temperature (E), and harvested to quantify lung CFUs (D) .

(F-H) Absolute numbers of (F) PMN (SSC^{hi} FSC^{hi} CD11b^{+} Ly6G^{+}), (G) ILC2s (Lin^{-} ST2^{+} ICOS^{+} CD25^{+} Thy1.2^{+}), (H) eosinophils (CD11b^{+} F4/80^{+} SSC^{hi} CD11c^{-} SiglecF^{+}) during the course of *S. pneumoniae* infection; (refers to Figure 4E-K).

(I) FACS plots illustrating lung eosinophil numbers in healthy WT and *Il5*$^{-/-}$ mice.

(J) WT and *Il5*$^{-/-}$ mice were i.n. infected with 10^5 CFU *S. pneumoniae* and sacrificed after 48h. CFU counts were assessed in lung homogenates (left) and blood (right).

(K) BAL isolated AMs from WT and *Il5*$^{-/-}$ mice were *in vitro* stimulated with *S. pneumoniae* (MOI 100). CXCL1 was quantified in supernatants.

Graph bars represent mean ± SEM. *$p < 0.05$, ***$p < 0.001$, ****$p < 0.0001$. Data are representative of two independent experiments with 4 (A, I and K) and at least 8 (B-H, and J) mice per group and/or time point.

Figure S6

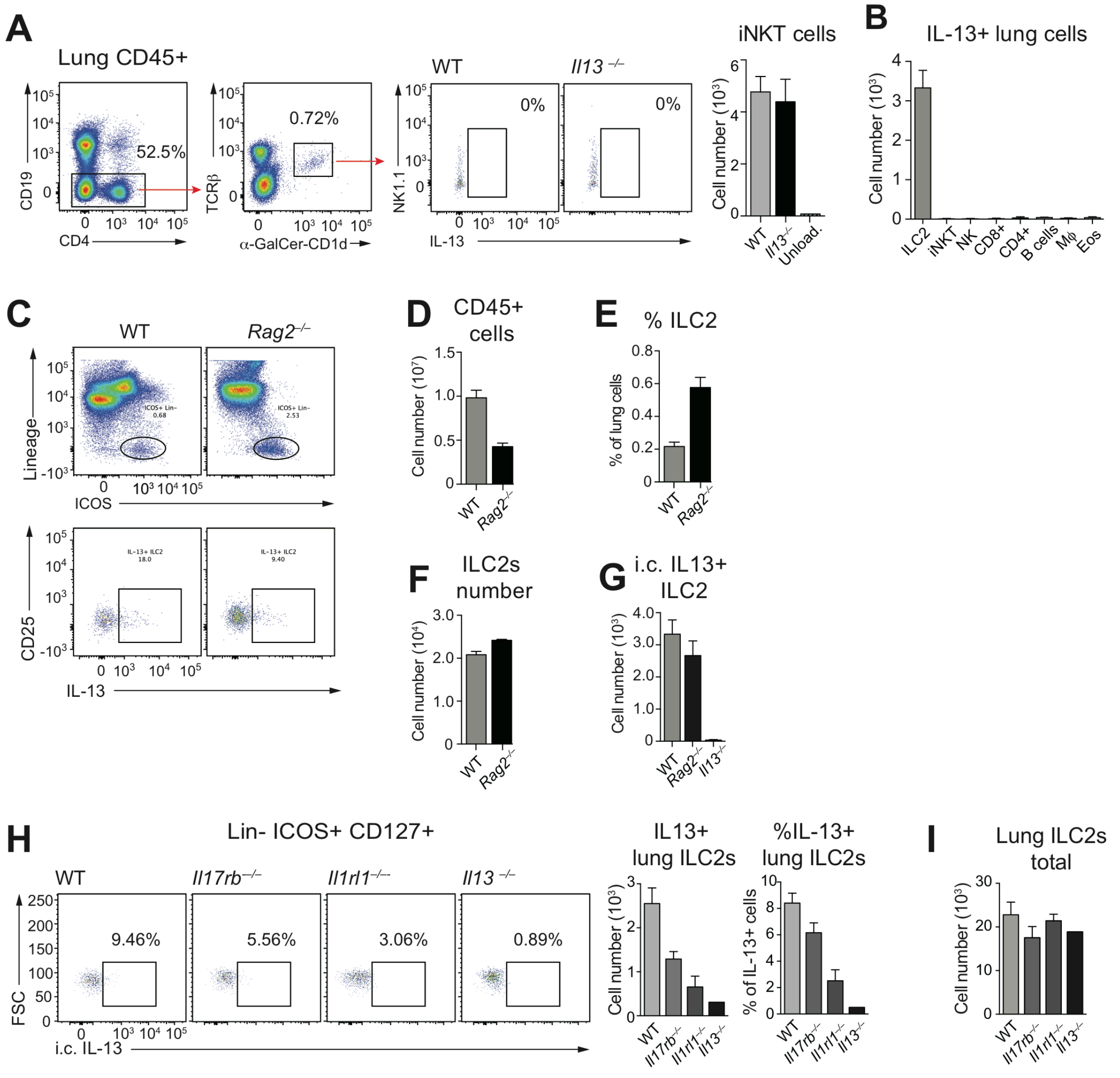

Figure S6. iNKT cells are not a source of IL-13 at homeostasis. Baseline ILC2-derived IL-13 expression does not depend or on B and T cells, but partially on *Il17rb*. Related to Figure 6.

(A) FACS gating strategy for iNKT cells (CD19^{-} CD4$^{+/-}$ TCRβ^{+} α-GalCer-CD1d dimer^{+} NK1.1^{+}). Unloaded CD1d dimer was used as negative control. Right panel: absolute number of iNKT cells in naive adult lungs. IL-13 production was assessed by i.c. staining.

(B) Absolute number of IL-13 secreting lung cells at homeostasis (cells gated as in Figure 6D). IL-13 production was assessed by i.c. staining. iNKT cells were gated as in Figure S6A.

(C-G) ILC2s were assessed for intracellular IL-13 production in naive WT and *Rag2*$^{-/-}$ mice at homeostasis. (C) Representative plots of ILC2s gating strategy. (D) Absolute lung cell number. (E) Percentage of ILC2s out of total lung cells. (F) Absolute number of ILC2s. (G) Absolute number of IL-13 expressing ILC2s.

(H) Representative FACS plots of ILC2s (Lin^{-} ST2^{+} ICOS^{+} Thy1.2^{+} CD25^{+}) assessed for IL-13 expression via i.c. staining in indicated mouse strains at homeostasis. Right: Absolute number and percentage of IL-13 secreting ILC2s.

(I) Absolute number of lung ILC2s at steady state in indicated mouse strains.

Single cells were pre-gated for viability and CD45+ expression. Graph bars represent mean ± SEM. Data are representative of at least two independent experiments with 4 mice per group. i.c. = intracellular.

Figure S7

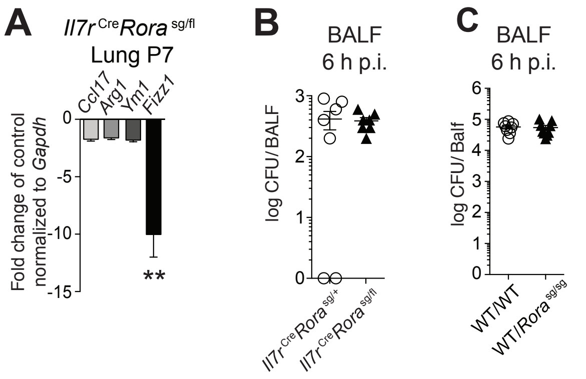

Figure S7. Reduced M2 marker expression in lungs of neonatal ILC2-deficient mice. Related to Fig 7.
(A) M2 markers were assessed by RT-PCR in whole lung homogenates of naive *Il7r*^{Cre}*Rora*$^{sg/fl}$ mice as compared to *Il7r*^{Cre}*Rora*$^{+/fl}$ controls on postnatal day 7 (P7), (refers to Figure 7B).
(B, C) BALF CFU in indicated mouse strains 6h post i.n. infection with *S. pneumoniae*, (refers to Figure 7E-J, and Figure 7O-P, respectively).
Graph bars represent mean ± SEM. **p < 0.01. Data are representative of at least two independent experiments with 4 mice per group (A) or 7-8 mice per group (B-C).

TABLES

Table S1. List of anti-mouse antibodies used in the study. Related to Figure 1-4 and 6-7.

Ab	Fluorochrome	Clone ID	Manufacturer	Isotype
B220	PerCP/Cy5.5	RA3-6B2	eBioscience	Rat IgG2a
CD45	V500	30-F11	BD Biosciences	Rat IgG2b
CD45	BV510	30-F11	Biolegend	Rat IgG2b
CD45.1	Pacific Blue	A20	Biolegend	Mouse IgG2a
CD45.2	PE	104	Biolegend	Mouse IgG2a
CD3	FITC, eFluor450	17A2	Biolegend	Rat IgG2b
CD3	PE/Cy7	145-2C11	Biolegend	Hamster IgG
CD4	FITC, PE/Cy7	GK1.5	eBioscience	Rat IgG2a
CD4	AF700, PE/Cy7, eFluor450	RM4-5	eBioscience	Rat IgG2a
CD8a	FITC, eFluor450, PE/Cy7	53-6.7	Biolegend or eBioscience	Rat IgG2a
CD11b	APC, FITC, AF700, PercP/Cy5.5, PE/Cy7, Pacific Blue	M1/70	Biolegend or eBioscience	Rat IgG2b
CD11c	FITC	HL3	BD Biosciences	Hamster IgG1
CD11c	AF700, PE/Cy7, BV421, AF647	N418	Biolegend or eBioscience	Hamster IgG
CD19	FITC, PE/Cy7	eBio1D3	eBioscience	Rat IgG2a
CD19	PE/Cy7, BV421, AF700	6D5	Biolegend	Rat IgG2a
CD31	PE/Cy7	390	eBioscience	Rat IgG2a
CD49b	FITC, Pacific Blue, PE/Cy7	DX5	Biolegend or eBioscience	Rat IgM
CD127	APC, PE/Cy7	A7R34	Biolegend	Rat IgG2a
CD138	APC	281-2	Biolegend	Rat IgG2a
c-Kit	AF700	ACK2	eBioscience	Rat IgG2b
EpCam	PE/Cy7	G8.8	Biolegend	Rat IgG2a
F4/80	BV421, BV785, FITC, PE/Cy7, APC, PercP/Cy5.5	BM8	Biolegend or eBioscience	Rat IgG2a
FceRIa	FITC, Pacific Blue, PE/Cy7, PE	MAR-1	Biolegend or eBioscience	Hamster IgG
Foxp3	APC	FJK-16s	eBioscience	Rat IgG2a
Gr-1	FITC, BV421, PE/Cy7	RB6-8C5	Biolegend or eBioscience	Rat IgG2b
ICOS	APC, PE, PE/Cy7, BV421	C398.4A	Biolegend or eBioscience	Hamster IgG
IL5	APC	TRFK5	Biolegend	Rat IgG1
IL13	PE	eBio13A	eBioscience	Rat IgG1

Ly6C	BV605	HK 1.4	Biolegend	Rat IgG2c
Ly6G	PE, PE/Cy7	1A8	Biolegend	Rat IgG2a
MHCII	FITC	2G9	BD Biosciences	Rat IgG2a
MHCII	Pacific Blue, BV510	M5/114.15.2	Biolegend	Rat IgG2b
NK1.1	eFluor450	PK136	eBioscience	Rat IgG2a
RORγt	PE	AFKJS-9	eBioscience	Rat IgG2a
Siglec-F	APC, AF647	E50-2440	BD Biosciences	Rat IgG2a
Sca-1	APC	D7	Biolegend	Rat IgG2a
ST2	Biotin, PerCp/Cy5.5, FITC	DJ8	MD Bioproducts	Rat IgG1
ST2	eFluor710	RMST2-2	eBioscience	Rat IgG2a
Ter-119	BV421, PE/Cy7	Ter-119	Biolegend	Rat IgG2b
Thy1.2	eFluor450	53-2.1	eBioscience	Rat IgG2a

Table S2. Gating strategy for lung cells in the study. All cells were gated on viable CD45+ cells. Related to Figure 1-4 and 6-7.

**Cell population**	**Gating strategy**
B cells	CD19+ MHCII+
CD4+ T cells	CD3+/CD4+ CD8-
CD8+ T cells	CD3+/CD4- CD8+
T regs	CD3+ CD4+ /CD25+ Foxp3+
ILC2	Lin-(CD3-CD4-CD5-CD8-CD19-CD49b-FceRIa-CD11b-Cd11c-F4/80-Gr1-) Thy1.2+ICOS+ST2+CD25+
NK cells	CD3- /NK1.1+ CD49b+
NKT cells	CD3+ /NK1.1+CD49b+
DCs	CD8-/F4/80-CD11c^{hi} /CD11b^{lo} MHCII^{hi}
AMs	F4/80+ CD11c+ /CD11b^{lo} SiglecF+
PMN	CD19-F4/80- Ly6G+
Eosinophils	CD19-F4/80+ CD11b+ CD11c- SiglecF+ SSC^{hi}
Mast cells	CD19- CD11b+ cKit+ FceRIa+

Table S3. List of primers used in the study. Related to Figure 1, 3-5 and 7.

**Gene target**	**NM name**	**Fragment size**	**Sequence**
mArg1	NM_007482	158bp	F: CAGTGTGGTGCTGGGTGGAG<br>R: ACACAGGTTGCCCATGCAGA
mYm1	NM_009892.1	191bp	F: TCTGGGTACAAGATCCCTGAACTG<br>R: GCTGCTCCATGGTCCTTCCA

mMrc1	NM_008625	116bp	F: TCTGGGCCATGAGGCTTCTC<br>R: CACGCAGCGCTTGTGATCTT
mFizz1	NM_020509	197bp	F: TCCAGCTGATGGTCCCAGTG<br>R: AAAGCCACAAGCACACCCAGT
mIL33	NM_001164724.1	174bp	F: CCCTGGTCCCGCCTTGCAAAA<br>R: AGTTCTCTTCATGCTTGGTACCCGA
mCXCL1	NM_008176	235bp	F: GACCATGGCTGGGATTCACC<br>R: TCAGAAGCCAGCGTTCACCA
mTNF	NM_013693	200bp	F: GAACTGGCAGAAGAGGCACT<br>R: GGTCTGGGCCATAGAACTGA
mHPRT	NM_013556	96bp	F: GTTAAGCAGTACAGCCCCAAAATG<br>R: AAATCCAACAAAGTCTGGCCTGTA

Supplemental Experimental Procedures:

Vacuum-induced lung stress. Lungs were harvested, stored on ice and transferred to either a pre-heated 37°C humidified control chamber or a pre-heated humidified vacuum chamber (Vacuubrand model MZ2C) (see Fig S1A). The vacuum was initiated and both groups were incubated for 6hs. Lungs were then dissociated using scissors and digested in collagenase 1 (750U/ml Invitrogen) and DNase1 (0.31mg/ml Roche) in RPMI (Life technologies) for 45 mins at 37°C. Tissues were passed through a 70µM filter using PBS with 2% FCS and processed for flow cytometry.

Lung flow cytometry and cell sorting. Lung single cell suspensions were prepared by incubating finely minced lung tissue for 1h at 37°C in RPMI containing 5% FCS, collagenase I (ThermoFischer), and DNase I (Sigma-Aldrich), homogenized with a glass homogenizer and then passed through a 70µm strainer. Cells were incubated for 5 min on ice in red blood cell lysis buffer (Sigma-Aldrich), washed and finally passed through a 40µm cell strainer. Single cell suspensions were counted with a hemocytometer and 2x10^{6} cells/stain were incubated with anti-mouse Fc receptor blocking antibody CD16/CD32 (eBioscience) and stained with a mix of fluorochrome labeled antibodies (**Supplementary Methods Table 1**). The alpha-GalCer/CD1d loaded dimer and unloaded control were provided by the NIH tetramer core facility. DAPI or fixable viability dye (eBioscience 65-0865) was added to the surface antibody mix to allow dead cells exclusion by flow cytometry. For gating strategies see **Supplementary Methods Table 2**. For intra-cellular (i.c.) staining, lung cell suspensions were further purified using Percoll (Sigma), incubated with PMA (60ng/ml), ionomycin (500ng/ml) and 1x protein transport inhibitor (eBioscience), or with brefeldin A (GolgiPlug™, BD Biosciences) for 4h at 37°C. Cells were then stained with surface antibodies, fixed and permeabilized before addition of anti mouse IL-13 mAb or anti-IL-5 mAbs, respectively, or isotype control Abs (Fixation and Permeabilization Solution Kit, BD Biosciences). For nuclear staining, cells were permeabilized and processed using the Foxp3 / Transcription Factor Staining Buffer Set (eBioscience). Acquisition was performed with a LSRFortessa (BD Biosciences) and data were analyzed using the FlowJo software version vX.0.7 (TreeStar). Cell sorting was performed with BD FACSAria™ III.

Reagents for isolation, culture and stimulation of alveolar macrophages (AMs). AMs from newborn mice were isolated by cell sorting from lung single cell suspensions as described above. In adult mice, AMs were isolated by bronchoalveolar lavage (BAL) followed by cell adhesion. In brief, mice were lethally anesthetized using 100 mg/kg body weight ketamine (Ketaset) and 10 mg/kg body weight xylazine (Rompun, Bayer), administered in sterile PBS i.p. AMs were isolated by inserting a tracheal cannula (Venflon, BD Bioscience) and flushing the lungs 10 times with 1ml NaCl. Isolated cells were counted and allowed to adhere at 37°C for 2h in RPMI containing 10% FCS and 1% penicillin/streptomycin at a concentration of 5x10^{4} cells/well in a 96 well plate. Wells were washed twice with PBS and adherent cells were used for further experiments.

Real time PCR. Total mRNA was isolated using the NucleoSpin RNA XS kit (Macherey-Nagel) or the RNeasy Micro kit (Qiagen) according to the manufacturers' instructions. Real-time PCR was performed using either the SYBR Green Master Mix (Applied Biosystems), or the TaqMan universal PCR mix (Applied Biosystems) and the StepOnePlus™ Real-Time PCR System (Applied Biosystems) or using a ViiA *7 (*Thermofisher*).* Commercially available Taqman probes were used for the expression of mCCL17 (Mm01244826_g1), mYm1 (Mm00657889_mH), mFizz1 (Mm00445109_m1), mArg1 (Mm00475988_m1) and GAPDH (Cat. 4352932E). Designed primers were purchased from Sigma-Aldrich and are listed in the **Supplementary Methods Table 3.** Gene expression was normalized to GAPDH or HPRT and expressed as fold change versus indicated controls.

Lung tissue homogenates and ELISA. Lungs were homogenized in the presence of protease inhibitors and total protein content was quantified by Pierce BCA Protein Assay Kit (Thermo Scientific). The Multiplex bead array (MILLIPLEX MAP Mouse Cytokine/Chemokine Magnetic Bead Panel - Premixed 25 Plex) was performed according to the manufacturer's instructions (Millipore), using MAGPIX multiplexing instrument and MILLIPLEX analysis 5.1 software (Millipore). Mouse IL-33 was measured using the eBioscience ELISA kit, according to the manufacturers' instructions. For protein quantification in *S. pneumoniae* infected lungs, CXCL1 and TNF were measured in lung homogenates using specific ELISA kits from RnD Systems, according to the manufacturers' instructions.

Analysis of alveolarization. H&E stained lung sections of newborn mice at postnatal day 7 were imaged using an Olympus FSX100 automated microscope and a 20X magnification (10 fields per lung). Lung alveolarization was quantified using the automatic image analysis software CellProfiler (Lamprecht et al., 2007) (http://www.cellprofiler.org/). Briefly, image masks for each field were generated by converting the images to grayscale and applying a binary threshold using the threshold function in ImageJ (version 1.49v; http://imagej.nih.gov/ij/). The original images were loaded into CellProfiler and alveoli were identified using the binary images generated in ImageJ (as described above), as the thresholding method using the *IdentifyPrimaryObjects* module. The shape and size features of the identified alveoli were then calculated using the *MeasureObjectSizeShape* module and exported to a spreadsheet format.

Immunofluorescence and analysis. Lungs from *Il33*$^{Cit/+}$ reporter mice were isolated at embryonic day 19 (E19) and postnatal days 1 (P1) and 3 and fixed overnight in 1% formaldehyde–PBS at 4 °C. After extensive washing with PBS, lungs were then incubated overnight in 30% sucrose solution at 4 °C. On the third day, lungs were embedded in 15% sucrose + 7.5% porcine skin gelatin (Sigma) in PBS and flash-frozen in isopentane chilled to −80 °C with liquid nitrogen. Lobes were sectioned to a 20-μm thickness onto Superfrost Plus slides (Thermo Scientific) and stored at −20 °C. Sections were air-dried for 1 h, rehydrated with PBS and then incubated in blocking buffer (3% goat serum (Jackson Immunoresearch) + 0.05% Triton-X in PBS) for 30 min. Sections were then incubated with a polyclonal rabbit anti-pro-surfactant protein-C antibody (Merck Millipore, AB3786) (1:500) in blocking buffer for 1 h. After washing, sections were then incubated with an Alexa Fluor 546-labeled polyclonal goat anti-rabbit antibody (4 μg/ml) (Thermo Fisher Scientific) and DAPI (300 nM) for 30 min in blocking buffer. After additional washes, Prolong Gold (Invitrogen) was added to slides plus a coverslip. Sections were imaged on a laser-scanning microscope (TCS SP8, Leica) with a 20×/ 20x/0.75NA HC PL APO CS2 air objective. Data were processed and analyzed using the open-source software ImageJ (Fiji package) (Schindelin et al., 2012).

Supplemental References:

Lamprecht, M.R., Sabatini, D.M., Carpenter, A.E., 2007. CellProfiler: Free, versatile software for automated biological image analysis. Biotechniques 42, 71–75. doi:10.2144/000112257

Schindelin, J., Arganda-Carreras, I., Frise, E., Kaynig, V., Longair, M., Pietzsch, T., Preibisch, S., Rueden, C., Saalfeld, S., Schmid, B., Tinevez, J.-Y., White, D.J., Hartenstein, V., Eliceiri, K., Tomancak, P., Cardona, A., 2012. Fiji: an open-source platform for biological-image analysis. Nat. Methods 9, 676–682. doi:10.1038/nmeth.2019
